# A High-Performance and Cost-Effective Field Programmable Gate Array-Based Motor Drive Emulator

**DOI:** 10.3390/mi14101864

**Published:** 2023-09-28

**Authors:** Julio Hernandez, Jose de Jesus Rangel-Magdaleno, Roberto Morales-Caporal

**Affiliations:** 1 Grupo de Sistemas Digitales (DSG), Coordinación de Electrónica, Instituto Nacional de Astrofísica óptica y Electrónica, Luis Enrique Erro # 1, Puebla 72840, Mexico; julio.hernandez@inaoep.mx; 2 División de Posgrado e Investigación, Instituto Tecnológico de Apizaco, Av Instituto Tecnológico s/n, Apizaco 90300, Mexico

**Keywords:** hardware emulator, FPGA, PMSM, motor-drive

## Abstract

This work presents a hardware-based digital emulator capable of digitally driving a permanent magnet synchronous machine electronic setup. The aim of this work is to present a high-performance, cost-effective, and portable complementary solution when new paradigms of electronic drive design are generated, such as machine early failure detection, fault-tolerant drive, and high-performance control strategy implementations. In order to achieve the high performance required by the digital emulator, the electronic drive models (permanent-magnet synchronous machine, voltage-source inverter, motor-control strategy) are digitally described in Verilog hardware description language and implemented on a field programmable gate array (FPGA) digital platform using two approaches: parallel and sequential methods. The results obtained show the effectiveness of the digital emulator design, and the resources used by the solution presented can be implemented on a low-cost digital platform that reveals a cost-effective operation of the solution presented.

## 1. Introduction

Due to the high performance, high power density, and time-to-market control strategies designed for it, permanent magnet synchronous machines (PMSM) are widely used nowadays. These machines are implemented in several industrial applications, such as robotics, electric traction, power generation, etc. [1].

Recently, new paradigms of electronic drive design have been developed in areas such as machine early failure detection, fault-tolerant drive, and high-performance control strategy implementations. For example, an extensive analysis of the fault conditions in induction machines is presented in [2,3] for broken rotor bars and bearing faults, respectively. These fault conditions are examined and modeled to present experimental results that show the benefits of early fault detection applied to the induction machine when several kinds of fault detection algorithms are implemented to detect the fault condition. Note that these algorithms require several sessions for data acquisition due to the amount of information needed to detect the fault condition. Also, in [4], a real-time fault detection for PMSM is presented. Here, the control strategy works along with the fault detection algorithm. A combination of digital signal processor (DSP) and FPGA is used to implement the control system where the DSP is responsible for the control strategy while the FPGA is used to implement a fast Fourier transform (FFT) to detect the spurious harmonics related to a fault applied; in this case, a connector fault is simulated, adding a 10 Ohm resistor in the machine phase. This work presents experimental results where the fault is detected when a predefined threshold for a particular harmonic is detected. In future work, different fault conditions are evaluated, such as inter-turn short-circuits.

These new electric machine drive paradigms increase the validation process efforts due to the adverse scenarios or extreme boundary conditions needed to verify the system’s performance. These new paradigms present the opportunity to change the design methodology, where electronic platforms can be used to create digital models of the target system, exit it by regular and extreme conditions in a safe manner, and use this information to validate the system designed, efficiently, in the real world. In this case, the simulator and emulator platforms are used to overcome the validation drawbacks mentioned when the electronic drive design is generated.

The software-based simulator and hardware-based emulator concepts are presented in [5]. The software-based simulator works with the main characteristics of the target system dynamics, allowing for the generation of a significant fast design process. On the other hand, the hardware-based emulator has the advantage of working with the actual hardware where the critical system properties can be described and implemented, giving inherent robustness and reliable characteristics in the system-level implementation.

A software-based simulator or hardware-based emulator methodology must be implemented according to the target system characteristics and the critical system information to be validated. Several works present solutions for simulator/emulator systems focused on electric machine drives. In [6], a simulator/emulator system is presented for induction machine fault analysis using software-in-the-loop (SIL). The SIL methodology is implemented on a dedicated computer where the induction machine model and fault detection algorithms are programmed and simulated in software for extended analysis. In [7], the optimal sampling time of a model-based predictive control strategy in an induction machine is presented using processor-in-the-loop (PIL). The PIL methodology allows one to program the target system in software and implement it in a DSP.

However, this kind of simulator/emulator system is implemented on dedicated digital platforms, making them susceptible to hardware and software support obsolescence.

In [8], an extensive study of the hardware-in-the-loop (HIL) platforms for electric machine drives is presented. The conceptual design to generate an HIL is described, and a detailed flow model-based design is analyzed. The HIL is a methodology that allows for the emulation of the entire control systems as well as the individual design stages. This generates a reduction in system execution interval and financial resources. This work also makes a review of several hardware platforms as computer-dedicated systems where specialized hardware and software are the fundamental parts of the HIL and the emulator realization.

A hardware emulator has the advantage of being also used in the physical validation as well as the system-level implementation. Several works have generated this kind of FPGA-based emulator focused on electric machine drives as follows.

A real-time emulator FPGA-based implementation of an induction machine is presented. The model is described in very high-speed integrated circuit hardware description language (VHDL) and implemented in an Intel Cyclone II EP2C70F672C6N [9]. In order to demonstrate its effectiveness, this design is generated with different fixed-point representations, giving an error accumulation analysis and the methodology to determine the sampling time and the integration method for the machine model.

A HIL evaluation of a PMSM drive is generated using XSG-Matlab/Simulink as platform description and implemented in a Xilinx Zedboard-Zynq development kit [5]. Here, the field-oriented control strategy is implemented along the PMSM digital model.

A real-time HIL implementation for a PMSM is generated using Matlab/Simulink and Altera DSP builder as design interface and implemented in Intel Cyclone III + Texas Instruments TMS320F28335 [10]. The aim of this work is to present the entire electronic drive model, including the PMSM model and the voltage source inverter (VSI) model in the FPGA.

A real-time HIL implementation for a PMSM is generated, operating in healthy conditions and with stator winding inter-turn faults using the Labview interface and implemented in the NI sb-RIO platform based on Xilinx Zynq-7010 SoC [11]. The fault injection is generated with a finite element approach model.

An HIL implementation for a brushless DC machine (BLDC) is generated using Typhoon HIL Schematic Editor version as the design interface and implemented on a dedicated Typhoon platform [12]. This work presents an implementation of cascaded linear controllers tuned by genetic algorithms applied to a BLDC in traction application.

An FPGA-based emulator has the portability characteristic, i.e., it can be implemented on several kinds of FPGA chip, with enough resources to handle the emulator design, no matter the fabrication technology or the fabrication company. However, the digital implementations of the related works reviewed avoid the portability characteristic due to the specialized software and hardware platforms used. In this work, an electric machine drive emulator is designed; a digital PMSM model, digital power electronics model, and novel control strategy are developed and described in Verilog hardware description language (HDL) using two different design methods: parallel and sequential. The entire system is implemented and validated in an FPGA-based platform obtaining satisfactory results in the electronics drive design scenario. The main contribution of this work is to present an FPGA-based electric machine drive emulator with high-performance, cost-effective, and portability characteristics.

## 2. Methodology

### 2.1. Power Stage

**Figure 1:**
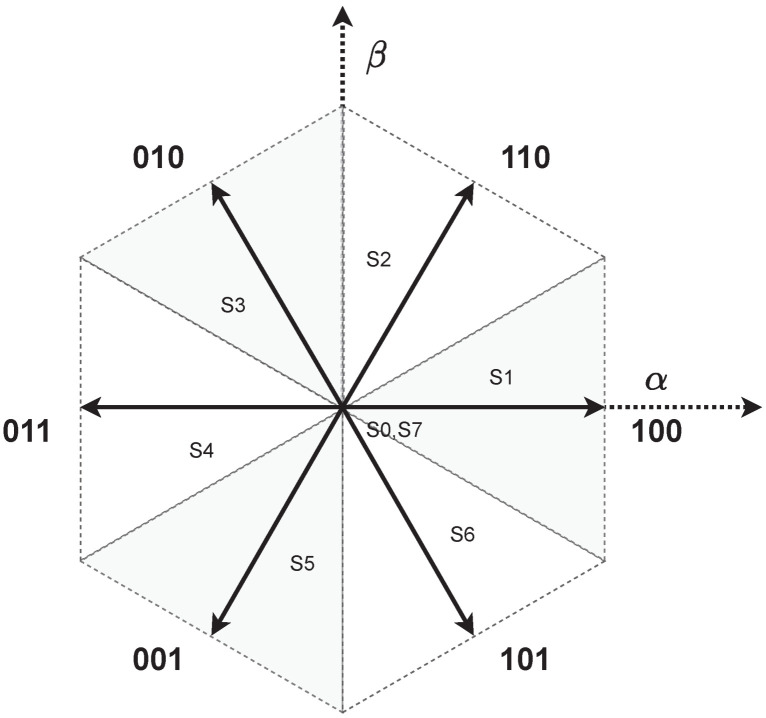
VSI voltage phasor: AVSV (S1, S2, S3, S4, S5, S6) and ZVSV (S0, S7).

**Figure 2:**
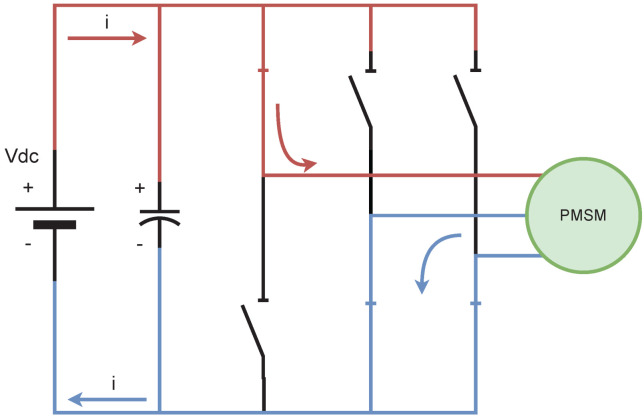
VSI voltage phasor operating principle when S1 is applied.

1. PMSM. The permanent magnet synchronous machine is a variant of a three-phase synchronous machine where the rotor magnetic field is applied by permanent magnets placed around the rotor shaft of the machine. The machine dynamics is described in (1)–(9) as follows. $u_{d,q}$, $i_{d,q}$ and $\psi_{d,q}$ are the voltage phasor components, current phasor components, and flux phasor components of the machine, respectively. $R_{s}$, $L_{s}$, $\psi_{f}$, and $P_{p}$ are the machine parameters: stator resistance, stator inductance, permanent magnet flux, and number of pole pairs, respectively. The $\omega_{m}$, $\omega_{e}$, $M_{e}$, $M_{l}$, and *J* are the mechanical and electrical rotor speed, electromagnetic and load torque, and the moment of the inertia, respectively.
  (1) $$\begin{array}{c} u_{d}=i_{d}R_{s}+\frac{d\psi_{d}}{dt}-\omega_{e}\psi_{q} \end{array}$$
  (2) $$\begin{array}{c} u_{q}=i_{q}R_{s}+\frac{d\psi_{q}}{dt}+\omega_{e}\psi_{d} \end{array}$$
  (3) $$\begin{array}{c} \psi_{d}=L_{s}i_{d}+\psi_{f} \end{array}$$
  (4) $$\begin{array}{c} \psi_{q}=L_{s}i_{q} \end{array}$$
  (5) $$\begin{array}{c} \frac{di_{d}}{dt}=\frac{1}{Ls}\left(u_{d}-i_{d}R_{s}+\omega_{e}\psi_{q}\right) \end{array}$$
  (6) $$\begin{array}{c} \frac{di_{q}}{dt}=\frac{1}{Ls}(u_{q}-i_{q}R_{s}-\omega_{e}\left(L_{s}\psi_{d}+\psi_{f}\right) \end{array}$$
  (7) $$\begin{array}{c} M_{e}=\frac{3}{2}P_{p}\left(\psi_{d}i_{q}-\psi_{q}i_{d}\right) \end{array}$$
  (8) $$\begin{array}{c} \frac{d\omega_{m}}{dt}=\frac{1}{J}\left(M_{e}-M_{l}-B\omega_{m}\right) \end{array}$$
  (9) $$\begin{array}{c} \omega_{e}=P_{p}\omega_{m} \end{array}$$
2. VSI and PSU. The voltage source inverter is the device needed to connect the power supply unit (PSU) and the electric machine. In this case, a three-phase and two-level configuration is implemented in the emulator design, which is generated with three parallel half-bridge discrete power devices. The VSI generates six active voltage phasors (AVSV) and two zero-voltage phasors (ZVSV); see Figure 1. According to the phasor selected, the machine is powered, as can be observed in Figure 2 when the voltage phasor S1 is selected. The voltage phasor selection is commanded by a digital platform according to the modulation generated by the control strategy. Table 1 shows the behavior of the voltage phasors in the VSI.

### 2.2. PWM Generator

**Figure 3:**
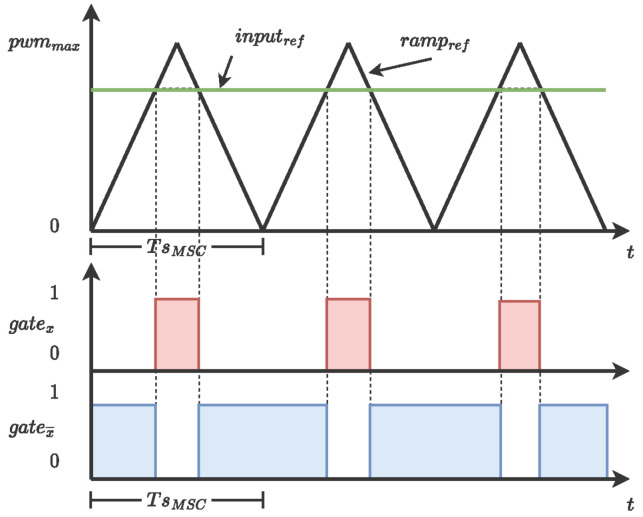
PWM signal operating principle.

**Figure 4:**
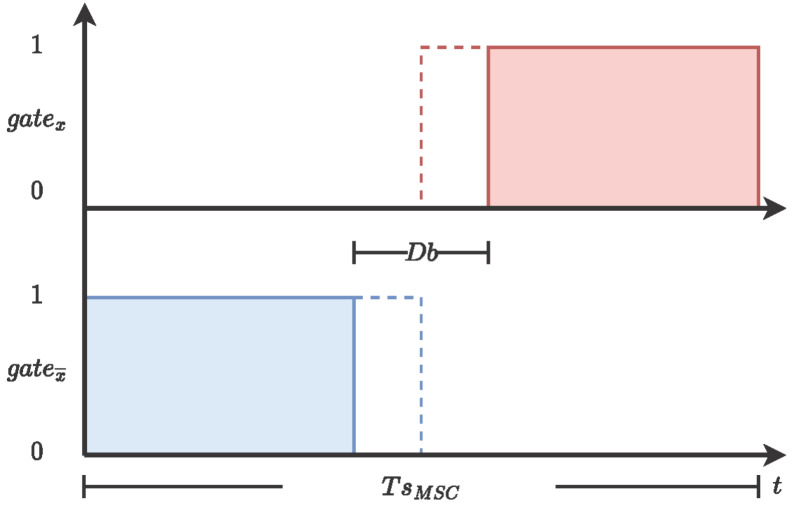
Dead-band generation.

The pulse width modulation generator is the block responsible for sending the gate combination to the VSI. There are six signals, one bit each, to send to the three half bridges in the VSI: the top section $\left(gate_{a,b,c}\right)$ and the bottom $\left(gate_{\bar{a,b,c}}\right)$ section. Figure 3 shows the conceptual design of the PWM generator. In order to obtain the signal modulation, the input signal is compared with a fixed-frequency reference ramp. If the input signal is greater than the reference ramp, the output bit $\left(gate_{x}\right)$ is zero $1^{\prime }b0$; otherwise, the output bit is one $1^{\prime }b1$, while the complementary signal $\left(gate_{\bar{x}}\right)$ has the opposite behavior. Furthermore, in order to avoid the cross-conduction scenario in the VSI, the PWM generator block implements a dead-band $\left(D_{b}\right)$, where a predefined interval is removed from top and bottom signals to assert the VSI performance; see Figure 4.

### 2.3. Encoder and Quadrature Decoder

**Figure 5:**
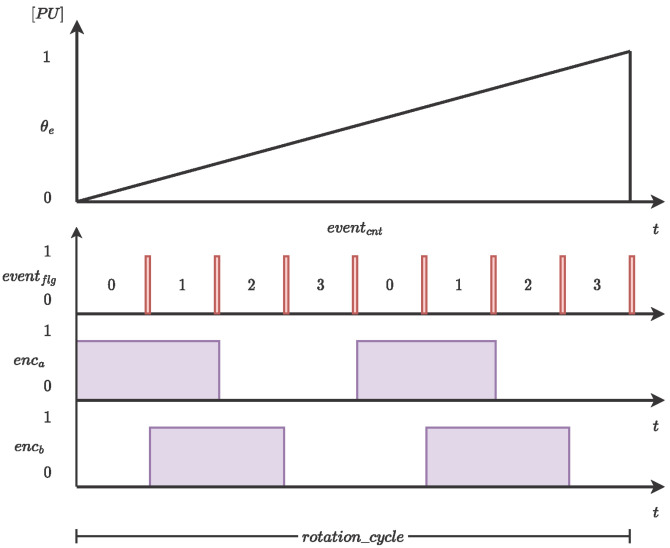
Encoder operational principle. The example shows eight slits per revolution.

The encoder (ENC) block is part of the PMSM design, and it is responsible for sending the information of the rotor mechanical position encoded in two bits $\left(enc_{a,b}\right)$ with a ninety-degree phase. Figure 5 shows the conceptual design of the ENC generator. First, the rotor mechanical position signal is adjusted at a per-unit approach (p.u.). Second, the mechanical rotor position per unit can be rearranged using a digital mask to obtain an even flag; the mask amplitude generates the number of slits in the encoder. The number of events is qualified and used to determine the block output as shown in Table 2 and Table 3 for rotor when clockwise and counterclockwise, respectively.

The quadrature decoder (QEP) block is responsible for decoding the information from the ENC block and reconstructing the rotor’s mechanical position. First, for each input channel $\left(enc_{a,b}\left(k\right)\right)$, the current input signals are compared with the previous ones $\left(enc_{a,b}\left(k-1\right)\right)$, respectively. The combination obtained increments/decrements are counter with the information of the rotor position signal reconstructed, as mentioned in Table 4. According to the direction information and the counter being greater/lower than a saturation reference, the counter is preset at zero or two pi, respectively.

### 2.4. Angular Speed Control

The angular speed control is a set of three blocks: speed determination (SPD), speed ramp generator (RAMP), and speed linear controller (PI).

**Figure 6:**
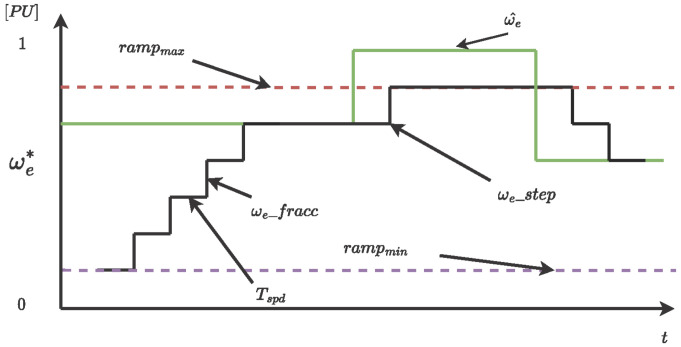
Speed reference ramp operating principle.

**Figure 7:**
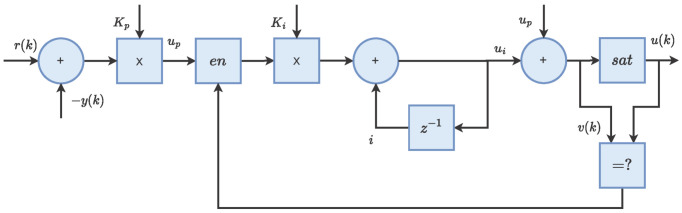
PI linear controller operating principle.

1. SPD. The SPD block is responsible for determining the feedback electrical speed ($\omega_{e}$) using the electrical rotor position $\left(\theta_{e}\right)$. First, this block calculates the difference of electrical rotor position at (k) and (k−1) instant. Using the fixed step sampling time of the linear-speed controller $\left(T_{spd}\right)$, a first-order low-22pass filter is implemented to determine the actual mechanical speed, as can be seen in (10)–(12), where $f_{b}$ is the PMSM base frequency, $\tau_{c}$ is the low-pass filter constant $\left(\tau_{c}=2\pi f_{c}^{-1}\right)$, and $f_{c}$ is the cut-off frequency of the filter.
  (10) $$\begin{array}{c} \omega_{e}\left(k\right)=K_{1}\left(\theta_{e}\left(k\right)-\theta_{e}\left(k-1\right)\right) \end{array}$$
  (11) $$\begin{array}{c} \hat{\omega_{e}\left(k\right)}=K_{2}\hat{\omega_{e}\left(k-1\right)}+K_{3}\omega_{e}\left(k\right) \end{array}$$
  (12) $$\begin{array}{c} K_{1}=\frac{1}{f_{b}T_{spd}}\\ K_{2}=\frac{\tau_{c}}{\tau_{c}+T_{spd}}\\ K_{3}=1-K_{2} \end{array}$$
2. RAMP. The RAMP block is responsible for determining the current speed reference to be applied to the linear controller. Starting from zero value, if the input speed requested is higher/lower than internal value, the system starts an increment/decrement in the target speed value at ramp fixed steps $\left(T_{spd}\right)$ as well as SPD block; the increment amplitude $\left(\omega_{e}\_fracc\right)$ is a constant value entered by the user, and ramp max limit $\left(ramp_{max}\right)$ and ramp min limit $\left(ramp_{min}\right)$ are the highest and lowest value allowed, respectively. The block behavior is presented in (13) and (14) and Figure 6.
  (13) $$\omega_{e}\_step=\omega_{e}\_step+\omega_{e}\_fracc$$
  (14) $$\omega_{e}^{*}=\left\{\begin{array}{cc} ramp_{max}: & \omega_{e}\_step>U_{max}\\ ramp_{min}: & \omega_{e}\_step<U_{min}\\ \omega_{e}\_step: & U_{min}<\omega_{e}\_step<U_{max} \end{array}\right.$$
3. PI. The speed linear controller is a proportional and integral controller (PI) in a series architecture with an anti-winding-up method. This speed controller is part of the motor control strategy discussed in Section 2.5. First, the proportional term $\left(u_{p}\left(k\right)\right)$ is calculated with the difference of the reference input (r(k)) and the feedback input (y(k)) and proportional gain $K_{p}$. Second, the integral term $\left(u_{i}\left(k\right)\right)$ is calculated using the previous integral information $\left(u_{i}\left(k-1\right)\right)$, $u_{p}\left(k\right)$ and the integral gain $K_{i}$. Finally, the auxiliary output v(k) is calculated from the system terms, and the block output (u(k)) is obtained from the saturation verification of v(k); the anti-winding-up signal w(k) allows one to remove the integral calculation if the output saturation is present; see in (15) to (19). Figure 7 shows the operational principle of the speed PI linear controller. Note that integrator sampling time is embedded in the (Ki) gain, which is the same as the other speed subblock $\left(T_{spd}\right)$.
  (15) $$\begin{array}{c} u_{p}\left(k\right)=K_{p}\left(r\left(k\right)-y\left(k\right)\right) \end{array}$$
  (16) $$\begin{array}{c} u_{i}\left(k\right)=u_{i}\left(k-1\right)+K_{i}u_{p}\left(k\right) \end{array}$$
  (17) $$\begin{array}{c} v\left(k\right)=u_{p}\left(k\right)+u_{i}\left(k\right) \end{array}$$
  (18) $$u\left(k\right)=\left\{\begin{array}{cc} U_{max}: & v\left(k\right)>U_{max}\\ U_{min}: & v\left(k\right)<U_{min}\\ v\left(k\right): & U_{min}<v\left(k\right)<U_{max} \end{array}\right.$$
  (19) $$w\left(k\right)=\left\{\begin{array}{cc} disable: & v\left(k\right)\neq u\left(k\right)\\ enable: & v\left(k\right)=u\left(k\right) \end{array}\right.$$

### 2.5. Control Strategy

As mentioned before, the SPC control strategy presented in [13] is used to drive the emulator system. This block is named as a motor control strategy (MSC) block in the following sections. The PCPCC is a model-based predictive control that is performed in the rotatory frame. The whole AVSV(k+1) and ZVSV are evaluated to determine the one to be applied in the next operating interval (k+1); for this reason, this strategy works in a fixed sampling time, obtaining activation interval $\left(t_{on}\right)$ values for each AVSV(k+1). The one that presents the minimum error in the $i_{d,q}$ reference is applied. With this information, the optimal voltage phasor is identified and used to calculate the duty ratio $\left(D_{ratio}\right)$ as (20) and (21).

(20) $$\begin{array}{c} D_{ratio}=\frac{|i_{d,q}^{*}-i_{d,q}\left(k+2\right)|}{C} \end{array}$$

(21) $$\begin{array}{c} C=\frac{|V_{dc}|}{L_{s}}T_{s} \end{array}$$

The PDTC is a model-based predictive control that is performed in the stationary frame. The AVSV and a ZVSV are selected according to the cost-function evaluation for the future torque required $\left(M_{e}\left(k+2\right)\right)$. As well as PCPCC, this strategy is performed in a fixed sampling time with the advance of implementing the optimal voltage phasor method to evaluate only two possible voltage phasors with two activation intervals $\left(pAVSV\left[2\right]@T_{on}\left[2\right]\right)$. In consequence, the motor control execution interval is reduced.

**Figure 8:**
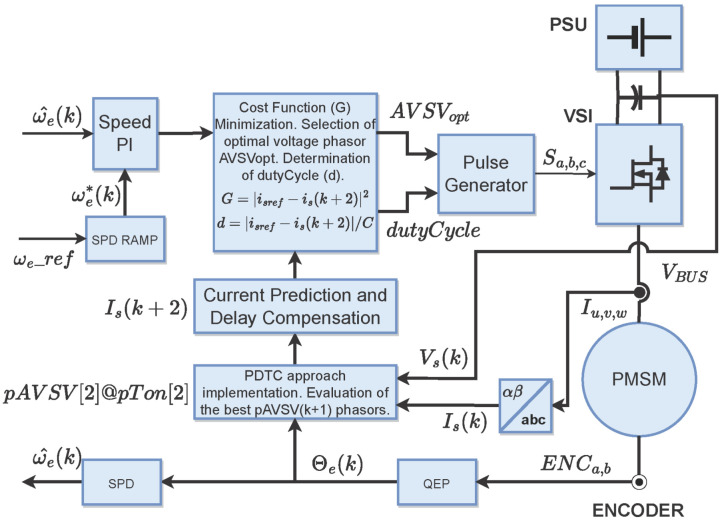
SPC block diagram.

The SPC implements the optimal phasor voltage method based on the $\psi_{\alpha ,\beta }\left(k+1\right)$; the best two AVSV are identified and used to determine the $t_{on}$ to be applied. The cost function minimization is obtained from the $i_{dq}\left(k+2\right)$ calculation. As a consequence, this approach decreases the operating time interval, keeping the performance of the PCPCC. The SPC block diagram is shown in Figure 8.

The control algorithm is described as follows:

1. Calculate the present and future sine and cosine of the electric rotor position, $sin(\theta_{e}\left[k\right])$,$cos(\theta_{e}\left[k\right])$,$sin(\theta_{e}\left[k+1\right])$,$cos(\theta_{e}\left[k+1\right])$.
2. Calculate the present alpha and beta current phasor components using the three-phase to stationary frame transformation, $I_{\alpha ,\beta }\left[k\right]$.
3. Calculate the present direct and quadrature current phasor components using the stationary to dynamic frame transformation, $I_{d,q}\left[k\right]$.
4. Calculate the present electric rotor angle position using the encoder signal, $\theta_{e}\left[k\right]$.
5. Calculate the present electric rotor angular speed using the electric rotor angle position, $\hat{\omega_{e}}\left[k\right]$.
6. Calculate the reference for the electric rotor angular speed using the present value set by the user, $\omega_{e}^{*}\left[k\right]$.
7. If $T_{speed}$ is performed, calculate the electric rotor speed command using the linear PI controller, $I_{d,q}^{*}$. Otherwise, keep the present command.
8. Determine the present alpha and beta voltage phasor components using the present AVSV applied, $V_{\alpha ,\beta }\left[k\right]$.
9. Calculate the present direct and quadrature voltage phasor components using the present alpha and beta voltage and the present sine and cosine of the electric rotor position, $V_{d,q}\left[k\right]$.
10. Calculate the future direct and quadrature current phasor components using the forward-Euler approach, $I_{d,q}\left[k+1\right]$; see [14,15].
11. Calculate the future alpha beta flux components using the forward-Euler approach, $\psi_{\alpha ,\beta }\left[k+1\right]$; see [1].
12. Using the future alpha beta flux components, calculate the future angle position, $\psi_{ang}$[k+1]; see [1].
13. Using the future flux angle position, determine the future flux sector, $\psi_{sector}\left[k+1\right]$; see [1].
14. Using the future angle position and the future quadrature current phasor component error, $I_{e}\left[k+1\right]$ (see [14,15]), determine the best two possible voltage phasors, $pAVSV_{\left(2\right)}\left[k+1\right]$, [1].
15. Determine the future alpha beta voltage phasor components using the information of the two best voltage phasors obtained previously, $V_{\alpha ,\beta }\left[k+1\right]$; see [1].
16. Calculate the future direct and quadrature voltage phasor components using the future alpha beta voltage phasors and the future sine cosine electric position, $V_{d,q}\left[k+1\right]$; see [14,15].
17. Calculate the future, [k+2], quadrature current phasor component using the forward-Euler approach, $I_{e}\left[k+2\right]$; see [14,15].
18. Determine the voltage phasor to be used in the next iteration and its activation duty ratio to be applied using the cost function, $AVSV\left[k+1\right]@t_{on}$; see [16].
19. Apply the next voltage phasor and its duty ratio to be performed in the pulse generator.
20. Wait for the next iteration.

### 2.6. Digital Design

**Figure 9:**
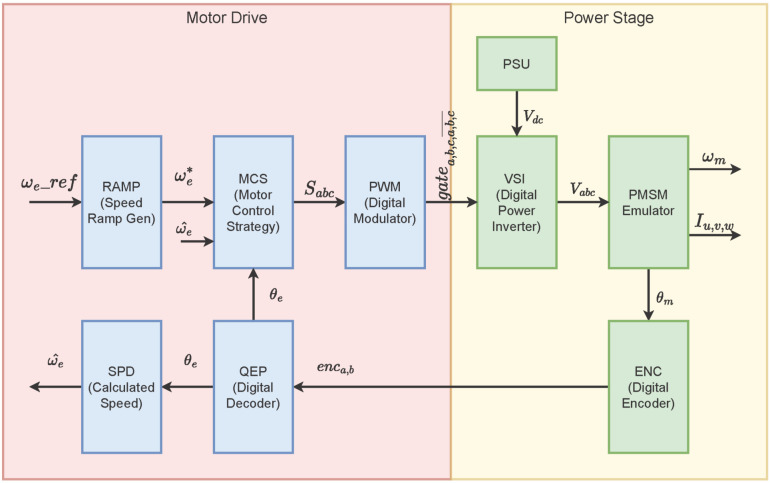
General block diagram of the digital emulator proposed.

As mentioned before, a digital emulator can be generated at several hardware platforms depending on the system characteristics, such as available resources precision level, system complexity, execution interval, etc. In this case, an FPGA-based solution is considered due to the amount of operations required to be implemented. The block diagram of the entire emulator proposal is shown in Figure 9. Each block represents part of the modules mentioned in previous sections to generate the electric machine drive for a PMSM.

As mentioned in [9], the integration method must be selected according to the execution interval. As long as the execution interval is highest, the integration method is complex. In this case, the emulator is designed using the forward-Euler method due to its inherent simplicity. This method accumulates the information of the last integration signal (y(t−1)) and the actual input signal u(n) to obtain the signal integration (y(t)) (22).

(22) $$\begin{array}{c} y(t)=y(t-1)+[t(n)-t(n-1)]u(n) \end{array}$$

**Figure 10:**
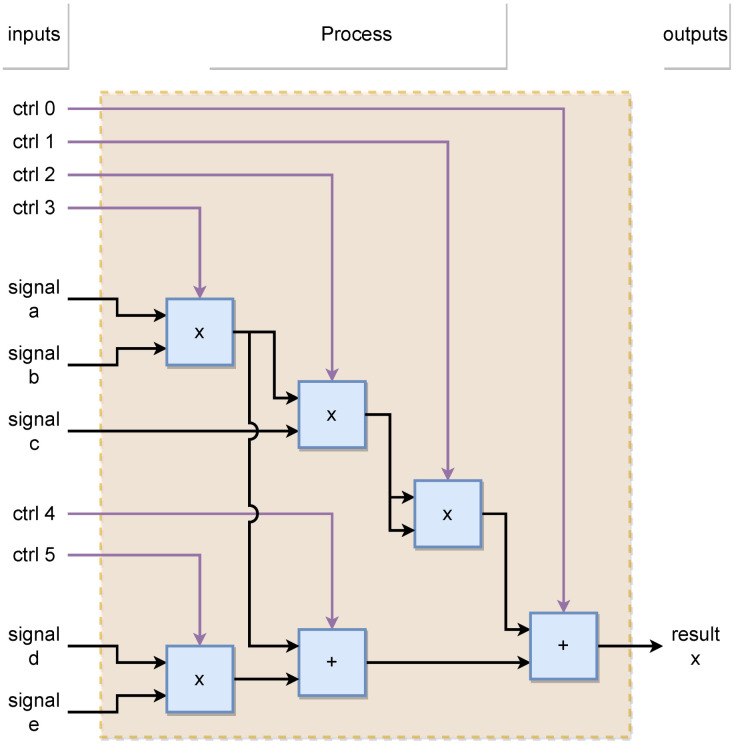
Design architecture in parallel method.

**Figure 11:**
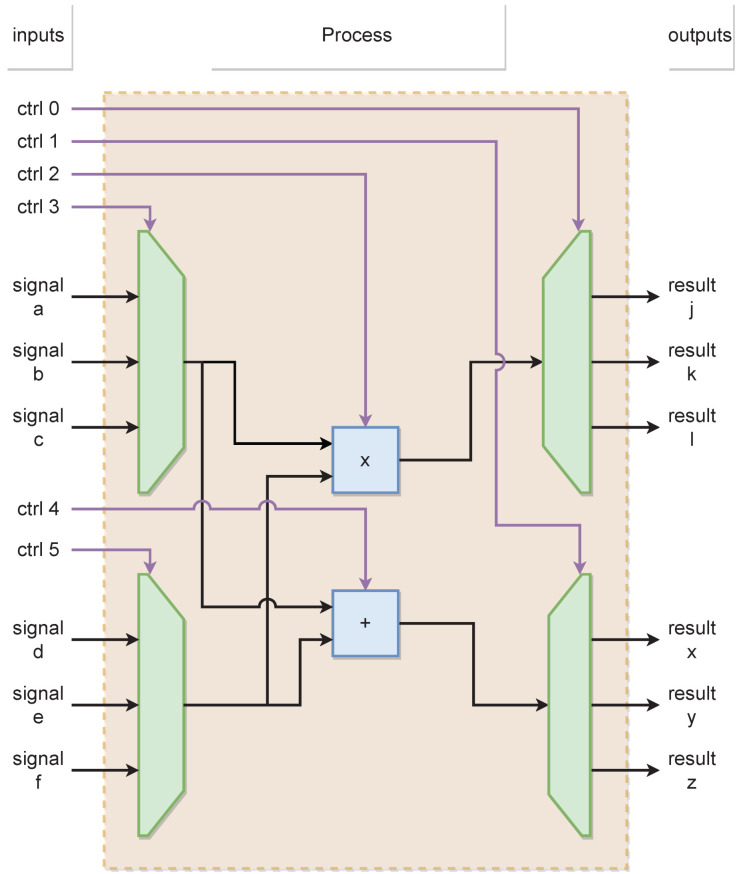
Design architecture at sequential method.

However, this requires the minimum possible execution interval for the PMSM model. For this reason, two design methods are presented (parallel and sequential) to mitigate the error accumulation due to the low-order integration method selected. Figure 10 and Figure 11 show the behavior of the parallel and sequential methods used to describe the system models in the emulator design, respectively.

The main advantage of the parallel method is the availability to perform operations in the same clock cycle, giving the process result in an instantaneous mode; when this method is applied on the FPGA platform, dedicated hardware elements are configured on the chip. If a large amount of resources is required, the system’s maximum frequency is reduced to keep the operation signal integrity due to the distance between the elements on the edge of the design.

On the other hand, the sequential method saves resources due to the input and output signal multiplexing with respect to the adders or embedded multipliers in the FPGA platform; however, this method generates an increment in the number of resources required, a look-up table (LUT), and a memory cell (flip-flop), used to multiplex the input and output of the operation blocks. For this reason, the maximum number of multiplexing lines and the total execution interval must be validated in the final implementation.

## 3. Results

### 3.1. Designed Emulator

**Figure 12:**
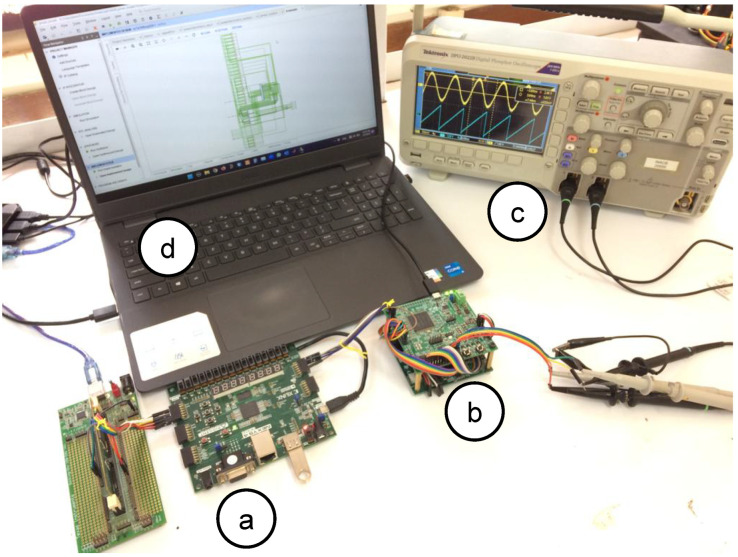
Experimental setup, (**a**) main digital platform, (**b**) DAC board, and (**c**) oscilloscope (**d**) host-PC.

The entire digital emulator is described in Verilog-HDL (digital PMSM model, digital power electronics model, and SPC control strategy). As mentioned before, the forward-Euler integration method is evaluated due to its inherent simplicity. The digital PMSM model parameters are shown in Table 5. The following results are shown in p.u. according to model parameter values. In order to obtain significant information about the solution designed, electronic drive devices (such as a PWM generator with dead-band implementation, a digital VSI model connected to the PSU, and quadrature encoder/decoder for PMSM rotor position detection) are considered as an interface between the digital PMSM model and the digital control strategy. The solution is implemented in the evaluation board NEXYS 4DDR with a Xilinx Artix-7 XC7A100T-CSG324C FPGA at 100 MHz clock source. The system variables are using a 32-bit resolution and a Q24 format for the decimal part. A 12-bit digital-to-analog converter (DAC) is used to visualize the digital PMSM model behavior on an oscilloscope. The Figure 12 shows the system setup connection.

**Figure 13:**
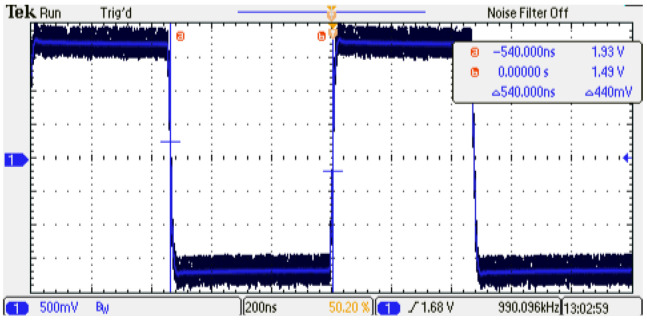
Execution interval of the digital PMSM model designed $Texec_{PMSM}=540$ ns, parallel method.

**Figure 14:**
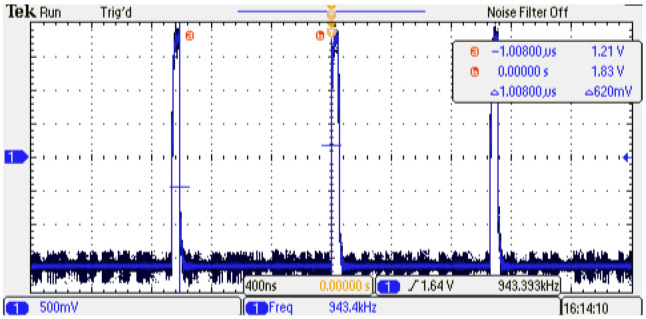
Execution interval of the digital PMSM model designed, $Texec_{PMSM}=1$ μs, sequential method.

In order to determine the minimum execution interval of the digital PMSM model $\left(Texec_{PMSM}\right)$, the parallel description method is used first. Figure 13 shows a $Texec_{PMSM}=540$ ns. On the other hand, when the sequential method is implemented, a $Texec_{PMSM}=1$ μs is observed; this is due to the increment in clock cycles needed to perform the whole operations in the digital PMSM implementation; Figure 14 shows the execution interval increment in the sequential method.

**Figure 15:**
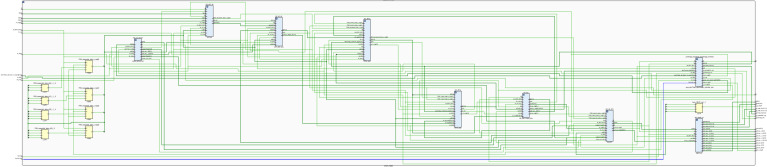
The RTL schematic of the digital PMSM model designed in the sequential method.

Figure 15 shows the register transfer level (RTL) of the digital PMSM designed. This RTL contains the digital implementations of the PMSM equations shown in (1)–(9). As can be observed, the blocks and connections represent the operations needed to implement the PMSM model. The input signals $V_{a,b,c}$, trg_load, and trg_load_reg are used to power the PMSM, determine the load torque value, and trigger the load torque in the PMSM, respectively. The rest of the input signals are used in the implementation structure. The output signals dac_data_ch0 and dac_data_ch1 are used to drive the DAC device with the $I_{a}\_p.u.$ and $\omega_{e}\_p.u.$ signals to be observed in the oscilloscope. The rest of the outputs are used in the internal MSC block and the ENC block, as mentioned in Figure 9.

**Figure 16:**
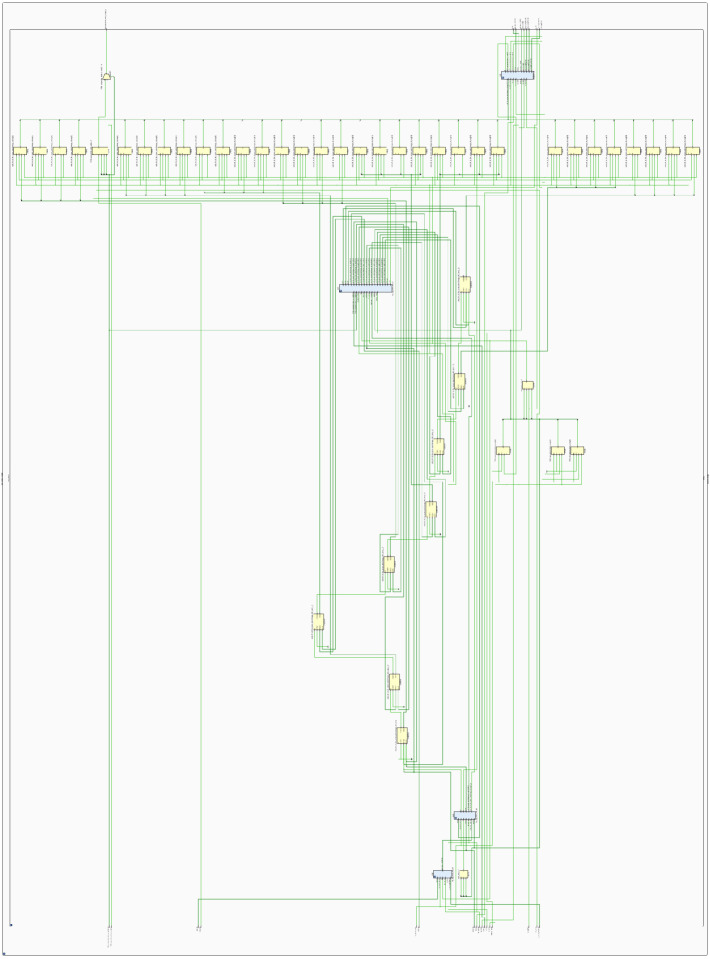
The RTL schematic zoom of $\psi_{d}$ transformation block, parallel method.

**Figure 17:**
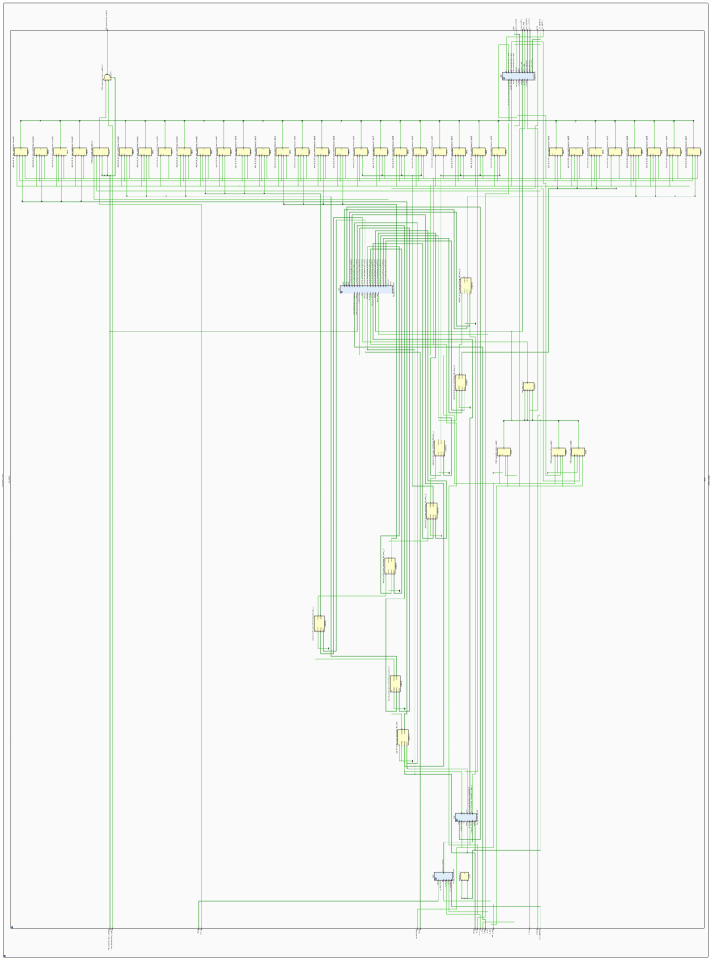
The RTL schematic zoom of $\psi_{d}$ transformation block, sequential method.

Figure 16 and Figure 17 show a zoom of the RTL of the $\psi_{d}$ generation block for the parallel and sequential approach, respectively. Here, the connection differences are noted where the elements used to generate the same block behavior have been changed significantly. Furthermore, in the sequence method connection, the signal multiplexing around the operation block is noted at the block inputs and output. This is verified in the following routing placement evidence.

As shown in Table 6, the system designed with the parallel method requires 63.33% of the embedded multipliers, 9.22% of the LUT, and 2.89% of the flip-flop due to the number of calculations performed. However, when the sequential approach is implemented (Table 7), only 47.5% of the embedded multipliers with an increment of 10.17% of LUT and 3.93% of the flip-flop is needed, saving 12% of resources.

**Figure 18:**
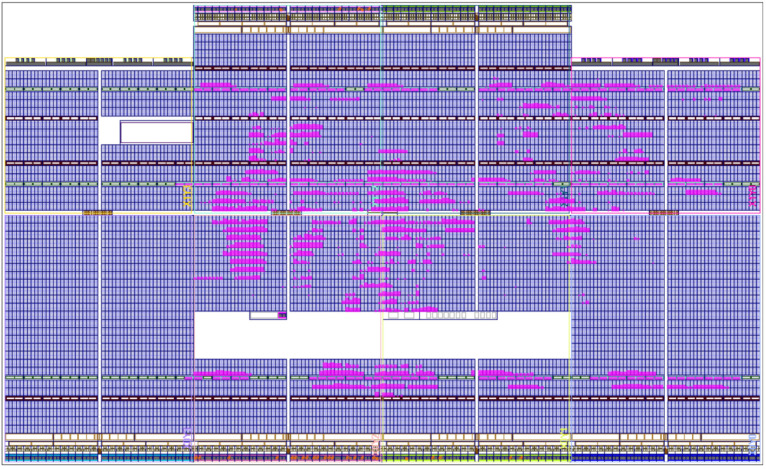
The routing placement of the digital PMSM model designed, parallel method.

In the following discussion, the magenta color is used to remark the FPGA chip resources, and the configurable logic block (CLB) is used in the implementations. As can be noticed, the parallel method distributes the CLB along the chip area; see Figure 18. Due to the connection distance between signals, which have to cross all chip sections, a timing degradation in the system connections is generated.

This phenomenon is known as worse negative slack (WNS), which is the setup slack of the critical path in the design. If WNS is negative, at least one path in the design does not meet the timing request.

**Figure 19:**
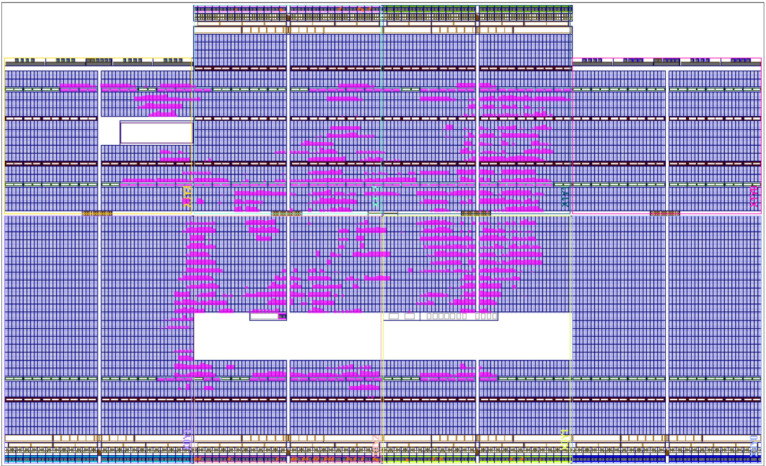
The routing placement of the digital PMSM model designed, sequential method.

In consequence, the design speed is reduced below the rated source clock. On the other hand, the sequential method compacts the CLB distribution; see Figure 19. This generates a positive WNS, which allows for the use of the rated source with a resource reduction characteristic.

### 3.2. Control Strategy Evaluation

As mentioned before, the SPC control strategy is described and implemented along the digital PMSM model in the emulator solution. Considering a maximum one hundred times faster relation between the digital PMSM model and control strategy execution interval $\left(Ts_{rate}\right)$, the control strategy is implemented on a fixed sampling time of one hundred microseconds ($Ts_{MSC}=100$ μs).

A minimum fixed sampling time of one microsecond is implemented for the digital PMSM model ($Ts_{PMSM}=1$ μs) in the parallel method. Although the sequential method allows one to reduce the system resources, it presents an 85% increment in the $Texec_{PMSM}$. For this reason, $Ts_{PMSM}=1.05$ μs is used for the sequential method. Due to the benefits obtained, the sequential method is used in the control strategy evaluation.

**Figure 20:**
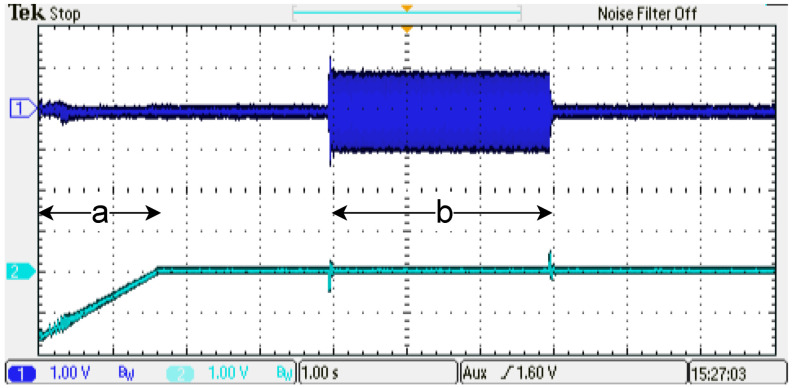
Experimental results, dynamic behavior of the emulator designed, speed reference 0.5 p.u. Channel 1: phase current U (±1 p.u. A/±1.65 V), offset=1.65 V. Channel 2: rotor angular speed (1 p.u. min${}^{-1}/3.3$ V), offset=1.65 V.

**Figure 21:**
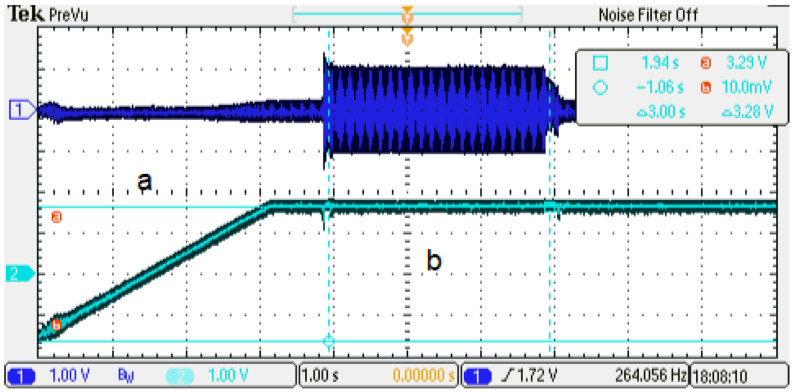
Experimental results, dynamic behavior of the emulator designed, speed reference 1.0 p.u. Channel 1: phase current U (±1 p.u. A/±1.65 V), offset=1.65 V. Channel 2: rotor angular speed (1 p.u. min${}^{-1}/3.3$ V), offset=1.65 V.

In order to verify the emulator performance, the control strategy discussed before is implemented in the emulator setup. The evaluation is performed under two conditions to determine the dynamic behavior in the operational range of the PMSM. A ten-second emulation is generated, and it is described as follows: (a) from [0–1.6] s, the speed ramp is applied to reach 0.5 p.u. nominal speed; (b) from [4–7] s, the 0.5 p.u. nominal torque is activated (see Figure 20). Furthermore, a ten-second emulation is generated, and it is described as follows: (a) from [0–3.1] s, the speed ramp is applied to reach 1.0 p.u. nominal speed; (b) from [4–7] s, the 0.5 p.u. nominal torque is activated (see Figure 21). In this case, the system presents a relevant difference in the phase-current behavior (Channel 1). The difference observed is generated by the oscilloscope acquisition. This is due to the increment in the phase-current frequency, which is synchronous to the rotor speed (Channel 2). As can be noticed, it is reaching the nominal speed (1 min${}^{-1}$ p.u.) along the phase-current frequency. The results obtained show that the emulator designed can be used in the full operating range of the PMSM rotor speed (system frequency).

**Figure 22:**
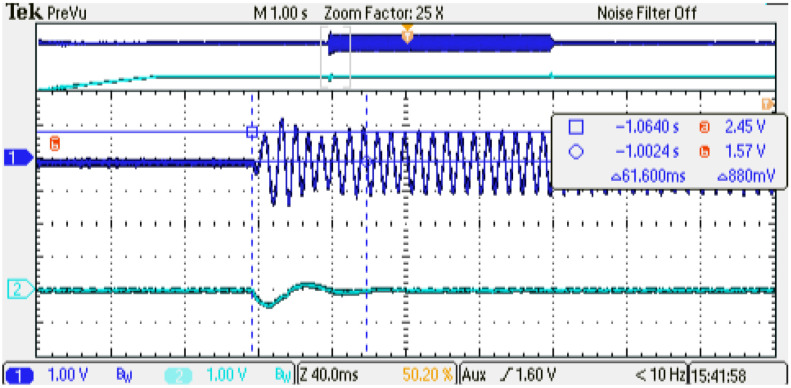
Experimental results, zoom dynamic behavior at load application. Channel 1: phase current U (±1 p.u. A/±1.65 V), offset=1.65 V. Channel 2: rotor angular speed (1p.u. min${}^{-1}/3.3$ V), offset=1.65 V.

**Figure 23:**
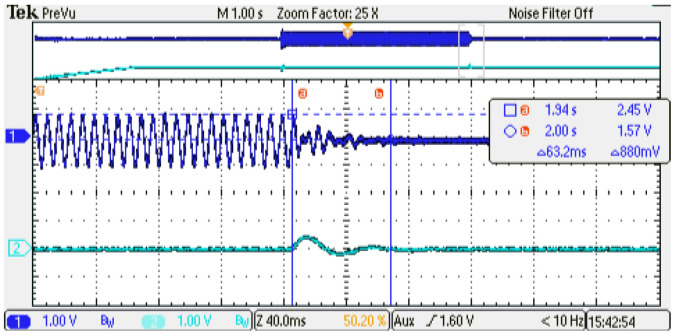
Experimental results, zoom dynamic behavior at load removal. Channel 1: phase current U (±1 p.u. A/±1.65 V), offset=1.65 V. Channel 2: rotor angular speed (1 p.u. min${}^{-1}/3.3$ V), offset=1.65 V.

Figure 22 and Figure 23 show a zoom of the load applied and a zoom of the load removal dynamic behavior of the digital PMSM model generated, respectively. As can be observed, the angular speed presents an overshoot of 12.5% while the recovery interval is around 63 ms, as configured in the linear-speed controller using the methodology presented in [17].

**Figure 24:**
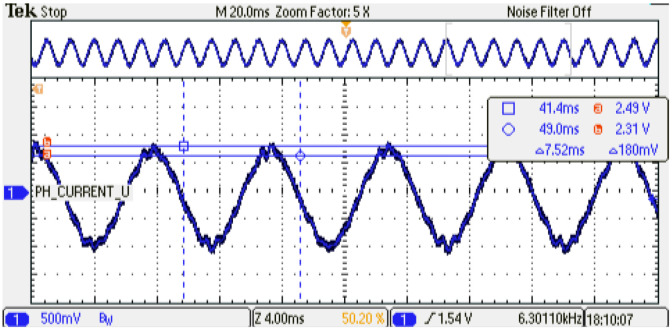
Phase-current behavior of the digital PMSM model. Sampling time: 1.5 us. Channel 1: phase current U (±1 p.u. A/±1.65 V), offset=1.65 V.

**Figure 25:**
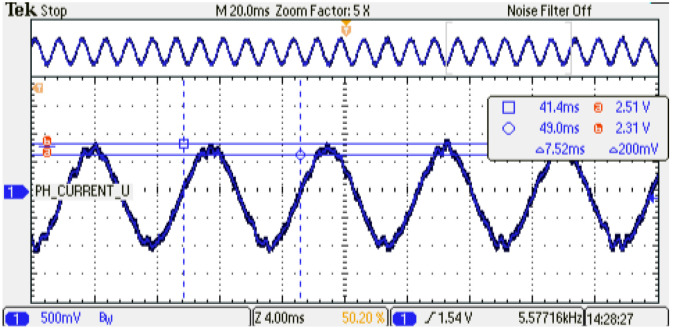
Phase-current behavior of the digital PMSM model. Sampling time: 2us. Channel 1: phase current U (±1 p.u. A/±1.65 V), offset=1.65 V.

**Figure 26:**
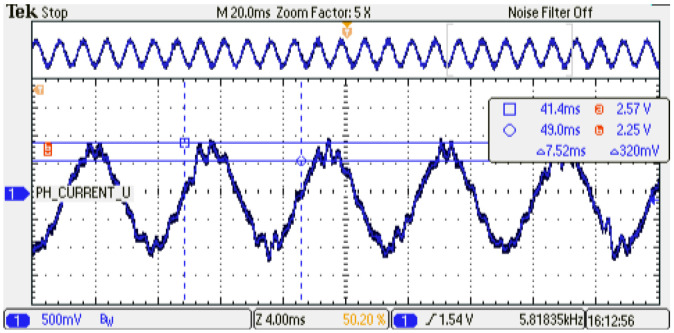
Phase-current behavior of the digital PMSM model. Sampling time: 4us. Channel 1: phase current U (±1 p.u. A/±1.65 V), offset=1.65 V.

**Figure 27:**
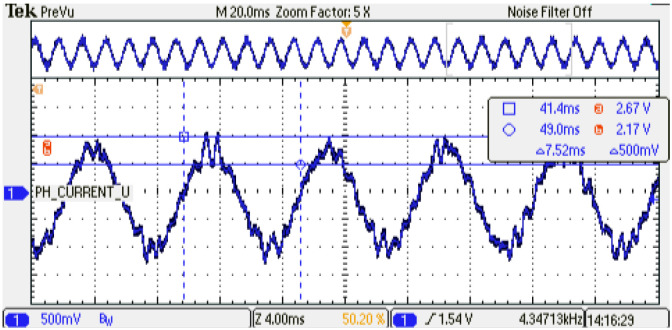
Phase-current behavior of the digital PMSM model. Dynamic condition failure. Sampling time: 6us. Channel 1: phase current U (±1 p.u. A/±1.65 V), offset=1.65 V.

The digital PMSM model designed is evaluated at several sampling time conditions where the phase current shows the performance of the emulator. As can be noticed, an increment in the sampling time of the digital PMSM model entails a smaller number of calculated iterations with respect to the control strategy. This degradation of the performance can be observed in the phase-current behavior at steady-state conditions; see Figure 24, Figure 25 and Figure 26 for $Ts_{PMSM}=\left[1.05,2.0,4.0\right]\mu s$, respectively. Although it is possible to drive the digital PMSM model at different sampling time conditions, the system degradation can be observed until 30% with respect to the maximum number of samples evaluated, where the system diverges in the dynamic condition; see Figure 27. Table 8 presents the results obtained at the sampling time conditions evaluated.

**Figure 28:**
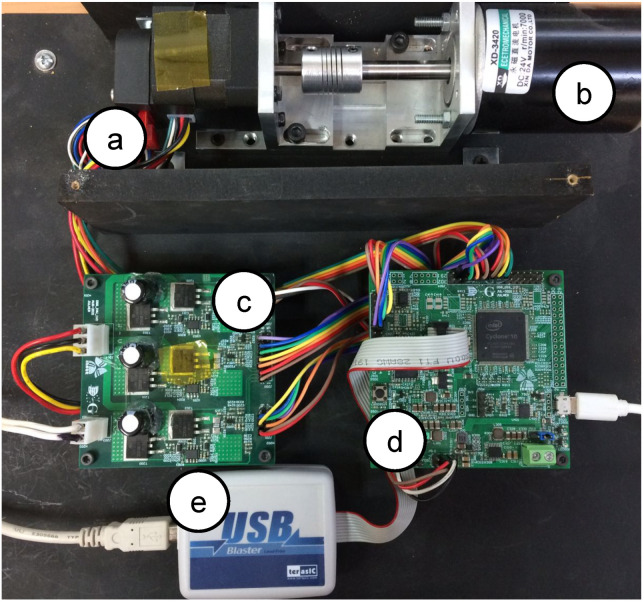
Experimental setup. (**a**) PMSM, (**b**) DC dynamometer, (**c**) VSI, (**d**) digital platform, and (**e**) programmer.

**Figure 29:**
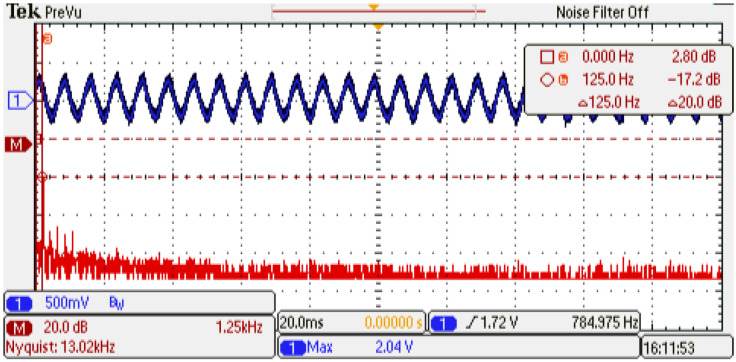
Frequency analysis of the physical setup. Channel 1: phase current U (±1 p.u. A/±1.65 V), offset=1.65 V. Channel 2: resultant FFT (vertical: 20 dB/div; horizontal: 1.25 kHz/div).

**Figure 30:**
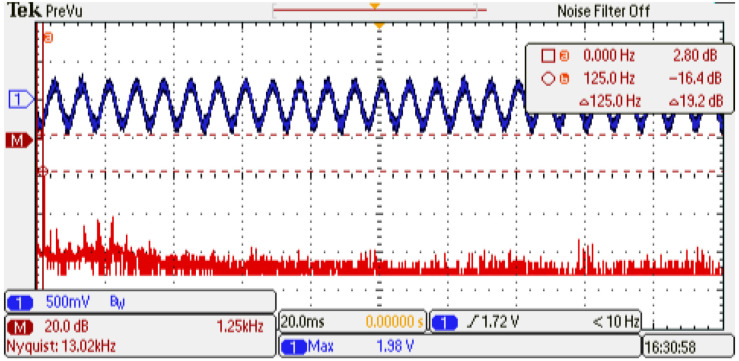
Frequency analysis of the emulator setup. Channel 1: phase current U (±1 p.u. A/±1.65 V), offset=1.65 V. Channel 2: resultant FFT (vertical: 20 dB/div; horizontal: 1.25 kHz/div).

An experimental evaluation of the physical PMSM drive is performed. The setup is a variant of the evaluation used in [13]; the emulator design presents similar parameter values as the experimental setup. Figure 28 shows the experimental setup and whole devices needed to complete the electric motor drive. This evaluation presents the operation conditions where the emulator is working. First, the system is driven at 0.5 p.u. nominal speed and a 0.15 p.u. nominal torque is applied. Second, the phase-current U data are saved and evaluated using the fast Fourier transform (FFT) application of the oscilloscope. Figure 29 shows the results obtained from the experimental setup. As remarked in the oscilloscope data, the system frequency is 133 Hz, corresponding to the 0.5 p.u. nominal speed, with an amplitude of −17.2 dB. Also, the same evaluation is performed on the emulator platform. In this case, the system frequency is 133 Hz, corresponding to the 0.5 p.u. nominal speed, with an amplitude of −16.4 dB; see Figure 30. There are some unexpected spurious harmonics around the 1 KHz band, which are undesired if implemented solutions, such as digital filters, are performed in that band. On the other hand, there is a significant difference between the amplitude of the 10 kHz component in the emulator and the corresponding in the experimental setup. In order to determine the accuracy of the emulator with respect to the experimental setup, a harmonic distortion analysis is performed using the phase current of both evaluations and the Matlab R2022b and its signal processing toolbox. Table 9 presents the results obtained from the harmonic distortion evaluation at 133 Hz. The emulator presents approximately three times the distortion factor with respect to the experimental setup due to the spurious harmonics detected. This behavior is related to the integration method and is considered in future work to overcome this drawback. The emulator system responds to the advanced control drive generation use case, and with the proper modifications, can be used in the system fault detection, early-fault diagnosis, and fault-tolerant electronic drive scenarios.

Additionally, the mean-square error (MSE) is obtained using (23), where *n* is the number of samples, *i* is the actual value evaluated, $Y_{i}$ is the observed value, and $\hat{Y_{i}}$ is the reference value. It is used to determine the average of the squares of the errors between both signals, the emulator, and the experimental setup, respectively. The result obtained is MSE = 0.0043, which is the performance of the system emulator with respect to the experimental setup. In future work, this MSE value will be reduced using other kinds of integration methods of superior order.

(23) $$\begin{array}{c} MSE=\frac{1}{n}\sum_{n-1}^{i}{\left(Y_{i}-\hat{Y_{i}}\right)}^{2} \end{array}$$

### 3.3. Low-Cost Implementation

Finally, considering the resource reduction viability in the sequential method, the digital PMSM design is implemented in a low-cost FPGA.

The DSG_DIG_2V0 custom-made board is a solution for general-purpose digital design implementation. This board presents five sections: the core, analog interface circuit, sensor circuit, main and secondary power supplies, communication ports, and digital user interface.

1. The core. This is an FPGA-based digital processing chip. The FPGA obtains the digital signals to process and send the calculations performed according to the control strategy. The peripherals around the FPGA are driven by the digital core. The general purpose input and output (GPIO) pins are driven by the 3.3 V PSU source.
2. The main and secondary power supply. The power supply unit is responsible for delivering the energy to the entire board. The main PSU can be configured to 5 V or 36 V as voltage input as needed; this PSU section is capable of driving 3.3 V as the main voltage rail in the board. Here, the secondary voltage rails are generated: 2.5 V, 1.8 V, and 1.2 V rails are generated by power converter units; these rail voltages are needed to drive the FPGA banks and the configuration section.
3. Analog interface. This is a set of ADC and DAC devices that can be used to acquire the system data or send the analog behavior outside via oscilloscope, respectively. These devices are driven by the 3.3 V PSU where the relative p.u. relative signals are scaled. These devices are driven by a serial peripheral interface (SPI) communication protocol as a slave device, where the FPGA is the master SPI device.
4. The sensor. In this case, the DSG_DIG_2V0 custom-made board is available to read its own temperature via a temperature sensor driven by an inter-integrated circuit-serial (I2C) communication protocol. This dedicated peripheral can also be used in general-purpose devices via an expansion connector.
5. The communication port is a set of programming devices as well as the universal asynchronous transition (UART) converter port. First, the programming port is responsible for the main configuration of the FPGA; this transmission is generated via a joint test action group (JTAG) protocol using a Terasic™ USB-Blaster. Second, a converter from UART to the universal serial bus (USB) is integrated to generate the link from the DSG_DIG_2V0 and the external host.
6. The digital interface. This section is a set of buttons and light emission diode (LED) indicators. The main purpose of this section is to provide the designer with a direct link with the board for debugging porpoises.

**Figure 31:**
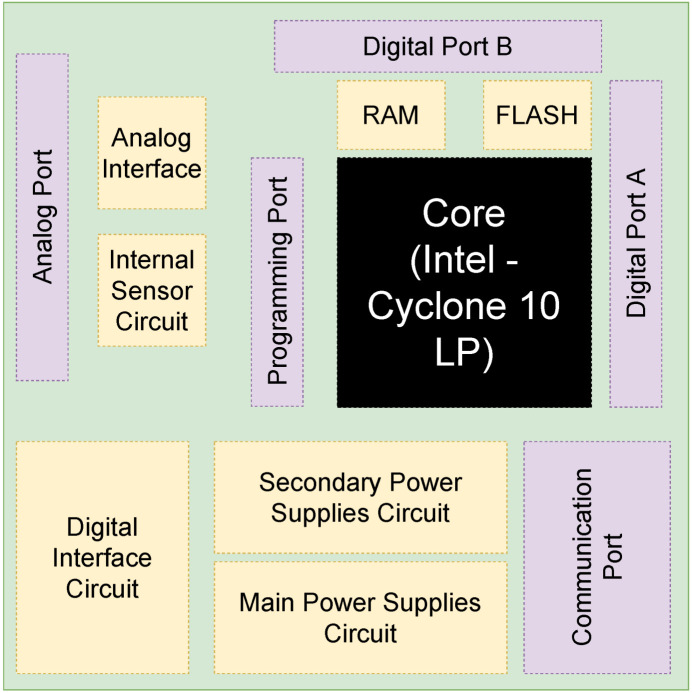
Block diagram of the DSG_DIG_2V0 custom-made board.

The general block diagram of the DSG_DIG_2V0 custom-made board is shown in Figure 31. Also, the conceptual board specifications are shown in Table 10.

**Figure 32:**
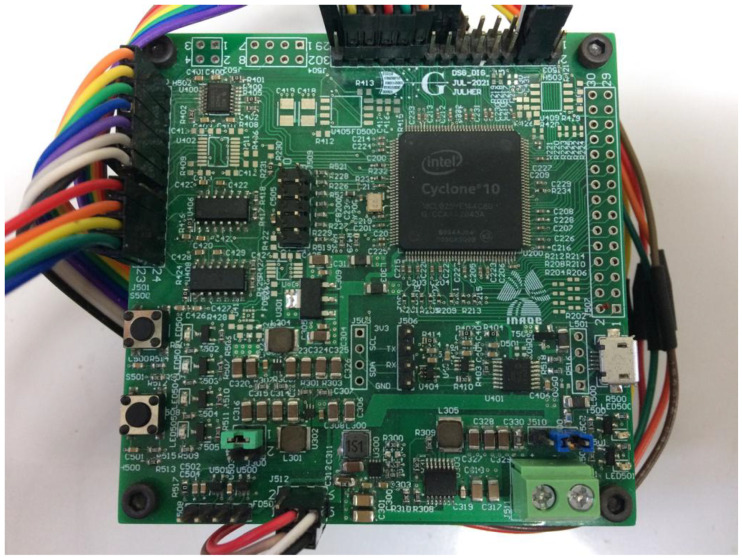
Custom-made digital platform, DSG_DIG_2V0.

When implemented, this is a custom-made digital platform with an Intel Cyclone 10LP FPGA 10CL025YE144C8G at a 100 MHz clock source. This chip has 25,000 logic elements (LE) and 132 9-bit embedded multipliers. This platform also integrates the 12-bit DAC mentioned before; see Figure 32.

**Figure 33:**
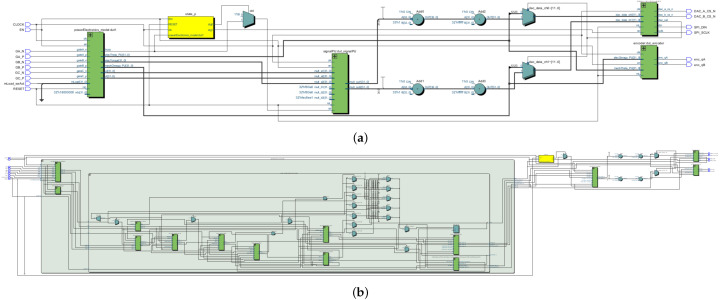
The RTL diagram of the digital power electronics model designed on Intel-Cyclone 10LP. (**a**) Full implementation and (**b**) zoom to the RTL diagram of the digital PMSM model.

Figure 33a,b show the full RTL of the power electronics designed and a zoom of the RTL of the PMSM design generated on Intel/Quartus 20.1.1, respectively.

Table 11 shows the resources summary of the implementation where the maximum frequency of the design is 78.93 MHz, which means an implementation where the maximum signal degradation is 10%, giving a cost-effective implementation of the emulator designed.

## 4. Conclusions

This work presents a high-performance PMSM drive emulator solution that is digitally implemented on an FPGA-based platform. The forward-Euler integration method is selected and evaluated due to its inherent simplicity. The digital PMSM model is described in Verilog-HDL using parallel and sequential methods. The digital PMSM model is validated using a closed-loop control strategy, where the associated electronic drive models (such as a PWM generator with dead-band implementation, a digital VSI model connected to the VDC, and a quadrature encoder/decoder for PMSM rotor position detection) are implemented along the main design. The results show that similar performance, with respect to physical power electronics and PMSM, is obtained in the steady-state and dynamic behavior when the model is evaluated with the SPC control strategy. The models are implemented on a medium-range FPGA mounted on a Nexys 4DDR (Xilinx-Artix7). The sequential method is used to verify the maximum sampling frequency obtained (1.05 MHz) and to report the output signal variations when the number of samples is reduced in the PMSM digital model. The sequential method can save up to 16% of the embedded multipliers needed with an increment of only 3% of LUT with respect to the parallel method. Furthermore, this approach allows for the use of a low-cost Cyclone 10LP, FPGA with 25,000 LE, and 132 9-bit embedded multipliers as a digital platform with a maximum main source clock frequency of 78.93 MHz, presenting a cost-effective solution for the digital PMSM drive emulator designed. These results confirm that the digital PMSM model can be used as a relevant and robust setup, keeping the portability characteristic of the FPGA-based implementation.

Unfortunately, spurious harmonics are detected when the results of the emulator design are compared with those of the experimental setup. However, this keeps allowing for the use-case scenario where the solution presents the advantage of managing the model and the environmental parameters as required in the design of PMSM control strategies such as advanced control drives. In future work, the solution presented can be implemented in other digital platforms to assure the portability characteristic and verify the effectiveness of the description method proposed, as well as to verify the effectiveness of the emulator system with different integration methods to overcome the drawback generated by the spurious harmonics and implement the emulator as a complementary design process in developing solutions such as system fault detection, early-fault diagnosis, and fault-tolerant electronic drives.

## Figures and Tables

**Table 1:** VSI behavior.

Voltage Phasor	$V_{a}$	$V_{b}$	$V_{c}$
S0(000)	0Vdc	0Vdc	0Vdc
S1(100)	$\frac{2}{3}Vdc$	$-\frac{1}{3}Vdc$	$-\frac{1}{3}Vdc$
S2(100)	$\frac{1}{3}Vdc$	$\frac{1}{3}Vdc$	$-\frac{2}{3}Vdc$
S3(100)	$-\frac{1}{3}Vdc$	$\frac{2}{3}Vdc$	$-\frac{1}{3}Vdc$
S4(100)	$-\frac{2}{3}Vdc$	$\frac{1}{3}Vdc$	$\frac{1}{3}Vdc$
S5(100)	$-\frac{1}{3}Vdc$	$-\frac{1}{3}Vdc$	$\frac{2}{3}Vdc$
S6(100)	$\frac{1}{3}Vdc$	$-\frac{2}{3}Vdc$	$\frac{1}{3}Vdc$
S7(111)	0Vdc	0Vdc	0Vdc

**Table 2:** Encoder output operation, rotor clockwise.

Event Count	${\mathrm{enc}}_{a}$	${\mathrm{enc}}_{b}$
0	1	0
1	1	1
2	0	1
3	0	0

**Table 3:** Encoder output operation, rotor counterclockwise.

Event Count	${\mathrm{enc}}_{a}$	${\mathrm{enc}}_{b}$
0	0	1
1	1	1
2	1	0
3	0	0

**Table 4:** Quadrature decoder position signal operation.

${\mathrm{enc}}_{a}\left(k\right)$	${\mathrm{enc}}_{b}\left(k\right)$	${\mathrm{enc}}_{a}\left(k-1\right)$	${\mathrm{enc}}_{b}\left(k-1\right)$	Direction	Position
0	0	1	0	1	+1
1	0	1	1	1	+1
1	1	0	1	1	+1
0	1	0	0	1	+1
0	0	0	1	0	−1
0	1	1	1	0	−1
1	1	1	0	0	−1
1	0	0	0	0	−1

**Table 5:** Parameters of the PMSM model.

Parameter	Units	Value
$R_{s}$	Ω	0.75
$L_{s}$	H	0.0105
$\omega_{r_{nom}}$	min${}^{-1}$	4000
$\psi_{f}$	V.s	0.005116
$M_{e}$	N.m	0.062
$P_{p}$		4
$V_{nom}$	V	24
$I_{nom}$	A	2.02
$Freq_{nom}$	Hz	266

**Table 6:** Digital emulator resources summary, parallel method.

	Utilization	Available	Percentage [%]
LUT	5845	63,400	9.22
FF	3667	126,800	2.89
DSP	152	240	63.33
IO	28	210	13.33
BUFG	1	32	3.13
Power	0.368 W	-	N.A
WNS	−0.29 ns	-	N.A
Delay	10.29 ns	-	N.A
Speed	97.181 MHz	-	N.A

**Table 7:** Digital emulator resources summary, sequential method.

	Utilization	Available	Percentage [%]
LUT	6449	63,400	10.17
FF	4983	126,800	3.93
DSP	114	240	47.5
IO	28	210	13.33
BUFG	1	32	3.13
Power	0.314 W	-	N.A
WNS	0.104 ns	-	N.A
Delay	9.89 ns	-	N.A
Speed	101.05 MHz	-	N.A

**Table 8:** Phase-current signal degradation with respect to PMSM sampling time.

${\mathrm{Ts}}_{\mathrm{PMSM}}$[μs]	${\mathrm{Ts}}_{\mathrm{rate}}$	Signal Degradation [%]
1.05	95.23	6.36
1.50	66.66	10.9
2.00	50.00	12.12
2.50	40.00	13.33
3.00	33.33	14.54
3.50	28.57	15.75
4.00	25.00	19.39
4.50	22.22	21.81
5.00	20.00	23.03
5.50	18.18	24.24
6.00	16.66	30.3

**Table 9:** Harmonic distortion evaluation of the experimental and emulator setup at 133 Hz.

Setup	Freq [Hz]	Distortion Attenuation [dB]	Distortion Factor [%]
Experimental	133	−34.0114	1.99
Emulator	133	−24.7256	5.8

**Table 10:** Specifications of the DSG_DIG_2V0 custom-made board.

Parameter	Unit	Min.	Typ.	Max.	Description
Size	mm		80 × 80		Size of printed circuit board (PCB).
PCB					Four-layer, FR4 dielectric.
Protection	IP		01		Protection grade.
Mounting			4		Mounting holes, size: M3.
Temperature	${}^{\circ }$C			50	Operating temperature.
Humidity	%			60	Relative humidity.
Heat-sink					Bottom board heat-sink.
Voltage	V			5.5/36	Power supply. USB/DC.
Current	A			1/3	Power consumption. USB/DC.
GPIO				52	GPIO pin.
ADC				4	ADC channels.
ADC-res	bit		12		ADC resolution.
DAC				2	DAC channels.
DAC-res	bit		12		DAC resolution.
Communication					USB-UART/I2C.
Sensor					Temperature sensor, I2C.
Interface				4/4	User interface: button/led.
Programming					JTAG programming interface.

**Table 11:** Digital emulator resources summary: sequential method on Intel-Cyclone 10LP.

	Utilization	Available	Percentage [%]
Logic Elements	4962	24,624	20
Registers	2785	N.A	N.A
Pins	16	77	21
Virtual Pins	0	N.A	0
Memory Bits	0	608256	0
Embedded Multiplier 9-bit Elements	104	132	79
PLL	0	4	0
Fmax	78.93 MHz	N.A	N.A

## Data Availability

Data are contained within the article or Supplementary Materials.

## Supplementary Materials

The following supporting information can be downloaded at: https://www.mdpi.com/article/10.3390/mi14101864/s1.
