# Supplementary material for: A High-Performance and Cost-Effective Field Programmable Gate Array-Based Motor Drive Emulator
Source: Micromachines (Basel). 2023 Sep 28;14(10):1864. doi: 10.3390/mi14101864 (PMC10608923; doi:10.3390/mi14101864)
Supplement: Supplementary file 1 [file micromachines-14-01864-s001.zip › micromachines-2585529-supplementary.pdf]

1 powerElectronics_top.v

This is a demo of the module powerElectronics_top.v and it is part of the Power Electronics Emulator project. This code is shared as a work-in-progress version due to the internal politics of our household Institute. The released version will be available upon request.

```
`timescale 1ns/100ps
// +FHDR ------------------------------------------------------------------------
// Copyright (c) 2020 INAOE All rights reserved
// INSTITUTO NACIONAL DE ASTROF?SICA, OPTICA Y ELECTRONICA
// COORDINACI?N DE ELECTR?NICA
// GRUPO DE SISTEMAS DIGITALES
// Confidential Proprietary
// ------------------------------------------------------------------------------
// FILE NAME  :   mcs_tb.v
// DEPARTMENT :   GRUPO DE SISTEMAS DIGITALES
// AUTHOR   :   Julio Hernandez
// AUTHOR?S EMAIL:    julio.hernandez@inaoep.mx
// ------------------------------------------------------------------------------
// RELEASE HISTORY
// VERSION DATE AUTHOR DESCRIPTION
//
// 1V0 220427 First test.
// ------------------------------------------------------------------------------
// PURPOSE  : This module implements a general purpose top level caller.
//        This module must be used as a bridge between Active-HDL and Quartus
// NOTE:    In Quartus the definicion of SYS_IMP must be activated.
// ------------------------------------------------------------------------------
// PROJECT PINOUT ASSIGMENT
//  WARNING: In order to use the DSG_DIG_2V0, dedicated pins must be configured as GPIO in Quartus
//        Go to: Assignments -> Device -> Device and Pin Options -> Dual-Purpose Pins, and change the value of the appropriate pin to Use as regular I/O.
//
// adc2dac.v    DSG_DIG_2V0   pinPlanner
// SPI_DIN      SPI_DIN     [7]
// SPI_DOUT     SPI_DOUT      [8]
// SPI_SCLK     SPI_SCLK      [6]
// ADC_CS_N     ADC_A_CS      [10]
// DAC_A_CS_N   DAC_AUX_CS    [11]  // This pin is used to drive the DAC_A or ADC_B on the DSG_DIG_2V0 board
// DAC_B_CS_N   DAC_B_CS      [13]
```

```
// FLASH_SC_N   B7_IO_44       [111] // This pin is used to drive the cs_n pin of the RAM or FLASH device on the DSG_DIG_2V0 board
//
// CLOCK         CLK        [23]
// SCL         SCL        [28]
// SDA         SDA        [31]
// UART_RX       UART_RX      [32]
// UART_TX       UART_TX      [33]
// LED[0]      B3_IO_12       [39]
// LED[1]      B3_IO_13       [42]
// LED[2]      B3_IO_14       [43]
// LED[3]      B3_IO_15       [44]
// RESET         B3_IO_16       [46]
// EN          B3_IO_17       [49]
// EN          B3_IO_17       [119] Use this for external enable
//
// DEBUG         B6_IO_39       [100]
// GC_P        B7_IO_51       [121]
// GB_P        B8_IO_53       [132]
// GA_P        B8_IO_55       [135]
// GC_N        B8_IO_57       [137]
// GB_N        B8_IO_59       [142]
// GA_N        B8_IO_61       [144]
// QEP[0]      B8_IO_56       [136]
// QEP[1]      B8_IO_58       [141]
// QEP[2]      B8_IO_60       [143]



// -FHDR ------------------------------------------------------------------------


`define SYS_IMP           1                      // Define the implementation condition
`define LEVEL             1                      // Define the implementation condition
`define SIM_QEP_CW          1                      // Define the clock-wise QEP simulation condition

`define SYS_DFT_DATA_WIDTH      32                      // Default system data width.
`define SYS_Q_FORMAT        24                      // Q format data width.
`define SYS_FREQ_kHz        100000                  // Main clock frequency in kHz.
//`define SYS_FREQ_kHz        50000                  // Main clock frequency in kHz.
`define SYS_PRD_ns          10                      // Main clock period in ns *** Warning this value must match with SYS_FREQ_kHz.

`define ADC_DATA_WIDTH        12    // Define data width of the adc module on DSG_ADC2DAC_1V1
```

```
`define ADC_CH_BIT          2   // Define number of bits needed to contain the adc channels on DSG_ADC2DAC_1V1; channels= 4 -> ADC_CH= log(4)/log(2)= 2
`define ADC_SPI_FREQ_kHz      6400  // DEVICE_FREQ_MHz= ((ADC_SPI_FREQ_kHz/SYS_FREQ_kHz)*SYS_CLK_FREQ_MHz)/2

`define DAC_DATA_WIDTH        12    // Define data width of the adc module on DSG_ADC2DAC_1V1
`define DAC_CH_BIT          2   // Define number of bits needed to contain the adc channels on DSG_ADC2DAC_1V1; channels= 4 -> ADC_CH= log(4)/log(2)= 2
`define DAC_SPI_FREQ_kHz      20000 // DEVICE_FREQ_MHz= ((DAC_SPI_FREQ_kHz/SYS_FREQ_kHz)*SYS_CLK_FREQ_MHz)/2

`define MULT_FIX_n_bit        `SYS_DFT_DATA_WIDTH         // Number of bit to contain the first input data.
`define MULT_FIX_m_bit        `MULT_FIX_n_bit << 1          // Number of bit to contain the multiplication data.
`define MULT_FIX_k_bit        `SYS_Q_FORMAT             // Number of shift bit to resize the multiplication at the input size.
`define MULT_ONE_PU          2** `MULT_FIX_k_bit          // Set a unit at the q format
`define MULT_TWO_PI          32'h06487ED5    // Set a unit at the q format

`define MATH_ONE_PU            32'h01000000  // ((1.0)(2*`SYS_Q_FORMAT))

`define RMP_CNT              32'h00000200  // ((0.0000305)*(2**`SYS_Q_FORMAT))
//`define RMP_CNT            32'h0000624E  // ((0.0015)*(2**`SYS_Q_FORMAT))
`define RMP_SAT_MAX            32'h01000000  // ((1.0)*(2**`SYS_Q_FORMAT))

`define MATH_ONE_PU            32'h01000000  // ((1.0)*(2**`SYS_Q_FORMAT))
`define MATH_TWO_PU            32'h02000000  // ((2.0)*(2**`SYS_Q_FORMAT))
`define MATH_ONE_OVER_SQRT_THREE_PU   32'h0093CD3A  // ((0.57735)*(2**`SYS_Q_FORMAT))
`define MATH_ONE_OVER_THREE       32'h00555555  // ((0.33333)*(2**`SYS_Q_FORMAT))

`define main_data_size    32
`define mult_data_size    64
`define qFormat_data_size   24


module powerElectronics_top
//#(  parameter   phCurr_scale_pu = 1016800, math_one_pu= 16777215, math_two_pi_pu= 105414357,vdc= 402653184, mLoad= 0 ) //, mLoad_value= 250000) //mLoad= 0, mLoad= 1053609
#(  parameter   phCurr_scale_pu = 1016800, math_one_pu= 16777215, math_one_over_two_pu= 8388608,math_two_pi_pu= 105414357, vdc= 402653184, elecTorque_scale_pu= 267153121, mLoad_value= 750000) //mLoad= 0, mLoad= 1053609
```

```
(
  input CLOCK, RESET, EN,

  input GA_P, GA_N, GB_P, GB_N, GC_P, GC_N,
  input mLoad_swAct,

//  input SPI_DOUT,
  output enc_qA, enc_qB,
//    output busy,
  output SPI_SCLK, SPI_DIN, DAC_A_CS_N, DAC_B_CS_N//, ADC_CS_N //FLASH_CS_N,

);

  // -----------------------------------------------------------------------------
  // Implementation I/O assigments
  // LEVEL: peripheral are validated
  // -----------------------------------------------------------------------------

    wire clk,rst,en;
//    wire adc_spi_dout;
//    wire clk_aux;

    // -----------------------------------------------------------------------------
    // ctrl and flg initialization
    localparam[2:0]
    idle    = 3'b100,   // Idle
    stg0    = 3'b000,   // Wait for the busy_pwrElec_model flag. 0: doNothing and keep the current state; 1: doMult_PU and go next state
    stg1    = 3'b001,   // Do doWriteDAC and go next state.
    stg2    = 3'b010;   // Wait for the busy_dac flag. 0: doNothing and keep the current state; 1: doNothing and go idle state.

    localparam [1:0]    // Control local parameters
    doNothing     = 2'b00,  // Idle
    doWriteDAC      = 2'b01,  //
    doMult_PU     = 2'b10;  //


    // -----------------------------------------------------------------------------
    // Top module local reg definition
    // ctrl : Run the Counter process
    reg [2:0] state_p, state_f;

    reg [1:0] ctrl;

    wire [1:0] stgDone;
```

```
    //
    ---------------------------------------------------------------------------
    // Wire & Reg local definition

    // DAC signal initialization
    wire [`SYS_DFT_DATA_WIDTH-1:0] dac_data_ch0, dac_data_ch1;
    wire str_dac, busy_dac, dac_spi_sclk, dac_spi_din, dac_a_cs_n,
    dac_b_cs_n, dac_sel; //, set_dac_spi;

    // PMSM signal initialization
    wire gateA_p, gateA_n, gateB_p, gateB_n, gateC_p, gateC_n;
    wire busy_pwrElec_model;
    wire str_MultPU;
//    (* keep = "true" *) (* dont_touch = "true" *) wire signed [
    `SYS_DFT_DATA_WIDTH-1:0] uI, vI, wI, elecTheta, mechOmega_PU,
    elecTorque;
//    (* keep = "true" *) (* dont_touch = "true" *) wire signed [
    `SYS_DFT_DATA_WIDTH-1:0] uI_pu, vI_pu, wI_pu;
//        wire signed [`SYS_DFT_DATA_WIDTH-1:0] elecTheta;
//        wire signed [`SYS_DFT_DATA_WIDTH-1:0] wI;
//        wire signed [`SYS_DFT_DATA_WIDTH-1:0] wI_pu;
//        wire signed [`SYS_DFT_DATA_WIDTH-1:0] wI_pu_n;

    wire signed [`SYS_DFT_DATA_WIDTH-1:0] uI, vI, mechOmega_PU,
    elecTorque, elecTheta_PU, elecTorque_PU;
        wire signed [`SYS_DFT_DATA_WIDTH-1:0] uI_pu, vI_pu;
        wire signed [`SYS_DFT_DATA_WIDTH-1:0] uI_pu_n, vI_pu_n;
        wire signed [`SYS_DFT_DATA_WIDTH-1:0] mLoad; // ,
    mLoad_value; //mLoad_value


    //
    ---------------------------------------------------------------------------
    // Top module initial conditions definition
    initial state_p = idle;

//    assign busy = state_p[2];

    assign clk  = CLOCK;

    assign rst  = ~RESET;        // DSG_DIG_2V0 BTN's High Common
    assign en = EN;          // DSG_DIG_2V0 BTN's High Common

    assign SPI_SCLK = dac_spi_sclk;
    assign SPI_DIN = dac_spi_din;

    assign DAC_A_CS_N = dac_a_cs_n;
    assign DAC_B_CS_N = dac_b_cs_n;

    assign gateA_p= GA_P;
    assign gateA_n= GA_N;
    assign gateB_p= GB_P;
    assign gateB_n= GB_N;
    assign gateC_p= GC_P;
    assign gateC_n= GC_N;
```

```
//
------------------------------------------------------------------------------
// Top module assignations definition

assign str_dac   =     ctrl[0];
assign str_MultPU =     ctrl[1];

assign stgDone[0] = busy_dac  ? 1'b1 : 1'b0;
assign stgDone[1] = busy_pwrElec_model  ? 1'b1 : 1'b0;
assign uI_pu_n= -uI_pu;
assign vI_pu_n= -vI_pu;
//    assign wI_pu_n= -wI_pu;

        assign mLoad = mLoad_swAct ? mLoad_value : 0;

//
------------------------------------------------------------------------------
// DAC configuration for zero amps

//    assign dac_data_ch0 = 32'd29360128 >>> 1;
//    assign dac_data_ch1 = 32'd4194304 >>> 1;

assign dac_data_ch0 = ~dac_sel ? (uI_pu_n + math_one_pu >>> 1)
: elecTheta_PU;
assign dac_data_ch1 = ~dac_sel ? (vI_pu_n + math_one_pu >>> 1)
: mechOmega_PU;

//    assign dac_data_ch0 = ~dac_sel ? (uI_pu_n + math_one_pu >>>
1) : elecTheta_PU;
//    assign dac_data_ch1 = ~dac_sel ? (vI_pu_n + math_one_pu >>>
1) : mechOmega_PU;

//    assign dac_data_ch0 = ~dac_sel ? (uI_pu_n + math_one_pu >>>
1) : (elecTorque_PU);
//        assign dac_data_ch1 = ~dac_sel ? (vI_pu_n + math_one_pu
>>> 1) : (mechOmega_PU);

// state machine activation
always @(state_p or en or stgDone)
  begin
    state_f <= state_p;
    ctrl <= doNothing;

    case(state_p)
      idle :
      begin
        if(en)
        begin
          state_f <= stg0;
          ctrl <= doNothing;
        end

      end
```

```
          stg0 :
          begin
            if(stgDone[1])
              begin
                state_f <= stg1;
                ctrl <= doMult_PU;
              end

          end

          stg1 :
          begin
            state_f <= stg2;
            ctrl <= doWriteDAC;
          end

          stg2 :
          begin
            state_f <= idle;
            ctrl <= doNothing;
          end

          default :
            begin
              ctrl <= doNothing;
              state_f <= idle;
            end
        endcase
      end

    always @(posedge clk)
      begin
        if (rst)
          state_p <= idle;
        else
          begin
            if (en)
              state_p <= state_f;
            else
              state_p <= idle;
          end
      end

//    (* DONT_TOUCH = "TRUE" *) powerElectronics_model
    powerElectronics_model
    #(.n(`main_data_size), .m(`mult_data_size), .k(
    `qFormat_data_size), .math_one_over_sqrt_three(9686330), .Rs
    (12582912), .Ts(16), .tdz(8), .tcm(100))
    dut1(
    .clk(clk),
    .rst(rst),
    .en(en),
    .busy(busy_pwrElec_model),

    .gateA_p(gateA_p),
    .gateA_n(gateA_n),
    .gateB_p(gateB_p),
```

```
    .gateB_n(gateB_n),
    .gateC_p(gateC_p),
    .gateC_n(gateC_n),
    .vdc(vdc),

    .mLoad(mLoad),
    .uI(uI),
    .vI(vI),
//    .wI(wI),
//    .elecTheta(elecTheta),
    .mechOmega_PU(mechOmega_PU),
    .elecTorque(elecTorque),
    .elecTheta_PU(elecTheta_PU)

    );

    dac_hdr
     #( .n(`DAC_DATA_WIDTH), .ch(`DAC_CH_BIT), .FREQ_FPGA_kHz(`SYS_FREQ_kHz), .FREQ_SPI_x2_kHz(`DAC_SPI_FREQ_kHz))
    dut_dac(
      .clk(clk),
      .rst(rst),
      .str(str_dac),
      .busy(busy_dac),
      .dac_data_ch0( dac_data_ch0[`MULT_FIX_k_bit-1:`MULT_FIX_k_bit-`DAC_DATA_WIDTH] ),
      .dac_data_ch1( dac_data_ch1[`MULT_FIX_k_bit-1:`MULT_FIX_k_bit-`DAC_DATA_WIDTH] ),
      .sclk(dac_spi_sclk),
      .din(dac_spi_din),
      .dac_a_cs_n(dac_a_cs_n),
      .dac_b_cs_n(dac_b_cs_n),
      .dac_sel(dac_sel)

    );

  encoder
        #(.n(`main_data_size), .m(`mult_data_size), .k(`qFormat_data_size))
        dut_encoder(
        .clk(clk),
        .rst(rst),
        .en(en),
        .elecOmega_PU(mechOmega_PU),
        .mechTheta_PU(elecTheta_PU),

        .enc_qA(enc_qA),
        .enc_qB(enc_qB)

        );

//    (* DONT_TOUCH = "TRUE" *) signalPU
    signalPU
        #(.n(`main_data_size), .m(`mult_data_size), .k(`qFormat_data_size))
        dut_signalPU(
        .clk(clk),
```

```
        .rst(rst),
        .en(en),
    .str(str_MultPU),

    .mult_a1(uI),
        .mult_b1(phCurr_scale_pu),
        .mult_out1(uI_pu),

    .mult_a2(vI),
        .mult_b2(phCurr_scale_pu),
        .mult_out2(vI_pu),

    .mult_a3(elecTorque),
        .mult_b3(elecTorque_scale_pu),
        .mult_out3(elecTorque_PU)

        );

//  fix_sig_mult_nbit #(.n(`main_data_size), .m(`mult_data_size), .k(`qFormat_data_size))
//  dut8
//  (
//    .clk(clk),
//    .rst(rst),
//    .en(en),
//    .str(str_MultPU),
//    .mult_a(uI),
//    .mult_b(phCurr_scale_pu),
//    .mult_out(uI_pu)
//  );

//  fix_sig_mult_nbit #(.n(`main_data_size), .m(`mult_data_size), .k(`qFormat_data_size))
//  dut9
//  (
//    .clk(clk),
//    .rst(rst),
//    .en(en),
//    .str(str_MultPU),
//    .mult_a(vI),
//    .mult_b(phCurr_scale_pu),
//    .mult_out(vI_pu)
//  );

////  fix_sig_mult_nbit #(.n(`main_data_size), .m(`mult_data_size), .k(`qFormat_data_size))
////  dut10
////  (
////    .clk(clk),
////    .rst(rst),
////    .en(en),
////    .str(str_MultPU),
////    .mult_a(wI),
////    .mult_b(phCurr_scale_pu),
////    .mult_out(wI_pu)
////  );

```

```
//  fix_sig_mult_nbit #(.n(`main_data_size), .m(`mult_data_size), .
    k(`qFormat_data_size))
//    dut11
//    (
//        .clk(clk),
//        .rst(rst),
//        .en(en),
//        .str(str_MultPU),
//        .mult_a(elecTorque),
//        .mult_b(elecTorque_scale_pu),
//        .mult_out(elecTorque_PU)
//    );



endmodule
```

2 powerElectronics_model.v

This is a demo of the module powerElectronics_model.v and it is part of the Power Electronics Emulator project. This code is shared as a work-in-progress version due to the internal politics of our household Institute. The released version will be available upon request.

```
// +FHDR ------------------------------------------------------------------------
// Copyright (c) 2020 INAOE All rights reserved
// INSTITUTO NACIONAL DE ASTROF?SICA, OPTICA Y ELECTRONICA
// COORDINACI?N DE ELECTR?NICA
// GRUPO DE SISTEMAS DIGITALES
// Confidential Proprietary
// -----------------------------------------------------------------------------
// FILE NAME  :   mcs_tb.v
// DEPARTMENT :   GRUPO DE SISTEMAS DIGITALES
// AUTHOR   :   Julio Hernandez
// AUTHOR?S EMAIL:    julio.hernandez@inaoep.mx
// -----------------------------------------------------------------------------
// RELEASE HISTORY
// VERSION DATE AUTHOR DESCRIPTION
//
// 1V0 220427 First test.
// -----------------------------------------------------------------------------
// PURPOSE  : This module implements a general purpose top level caller.
//        This module must be used as a bridge between Active-HDL and Quartus
// NOTE:    In Quartus the definicion of SYS_IMP must be activated.
// -----------------------------------------------------------------------------
// PROJECT PINOUT ASSIGMENT
//  WARNING: In order to use the DSG_DIG_2V0, dedicated pins must be configured as GPIO in Quartus
//        Go to: Assignments -> Device -> Device and Pin Options -> Dual-Purpose Pins, and change the value of the appropriate pin to Use as regular I/O.
//
// adc2dac.v    DSG_DIG_2V0   pinPlanner
// SPI_DIN      SPI_DIN     [7]
// SPI_DOUT     SPI_DOUT      [8]
// SPI_SCLK     SPI_SCLK      [6]
// ADC_CS_N     ADC_A_CS      [10]
// DAC_A_CS_N   DAC_AUX_CS    [11]  // This pin is used to drive the DAC_A or ADC_B on the DSG_DIG_2V0 board
// DAC_B_CS_N   DAC_B_CS      [13]
// FLASH_SC_N   B7_IO_44      [111] // This pin is used to drive the cs_n pin of the RAM or FLASH device on the DSG_DIG_2V0
```

```
    board
//

// -FHDR ------------------------------------------------------------------------

module powerElectronics_model
#(  parameter   n = 32, m= 64, k=24, math_one_over_sqrt_three= 9686330, Rs= 12582912, Ts= 16, tdz= 8, tcm= 100)
(
  input clk, rst, en,
  output busy,

  input gateA_p, gateA_n, gateB_p, gateB_n, gateC_p, gateC_n,
  input signed [n-1:0] vdc, mLoad,
//  output signed [n-1:0] wI elecTheta,
  output signed [n-1:0] uI, vI, mechOmega_PU, elecTorque, elecTheta_PU

);

// -----------------------------------------------------------------------------
// Wire & Reg local definition

  // abcV signal initialization
  wire signed [n-1:0] aV,bV,cV;

  // pmsm signal initialization
  wire str_pmsm, busy_pmsm;

// -----------------------------------------------------------------------------
// Top module assignations definition
  assign busy = busy_pmsm;

// -----------------------------------------------------------------------------
// Top module assignations definition


  vsi_digModel
  #(.n(n) )
  dut_vsi_digModel(
  .clk(clk),
  .rst(rst),
  .en(en),
  .gateA_p(gateA_p),
  .gateA_n(gateA_n),
  .gateB_p(gateB_p),
  .gateB_n(gateB_n),
  .gateC_p(gateC_p),
  .gateC_n(gateC_n),
```

```
  .vdc(vdc),
  .aV(aV),
  .bV(bV),
  .cV(cV)
  );

  clkTrigger
  #(.n(tdz), .trg_count_max(tcm) )
  dut_clkTrigger(
  .clk(clk),
  .rst(rst),
  .en(en),
  .trg(str_pmsm)
  );

//  pmsm_model
//  #(.n(n), .m(m), .k(k), .math_one_over_sqrt_three(
    math_one_over_sqrt_three), .Rs(Rs), .Ts(Ts))
//  dut_pmsm_model(
//  .clk(clk),
//  .rst(rst),
//  .en(en),
//  .str(str_pmsm),
//  .busy(busy_pmsm),
//  .aV(aV),
//  .bV(bV),
//  .cV(cV),
//  .mLoad(mLoad),
//  .uI(uI),
//  .vI(vI),
////  .wI(wI),
////  .elecTheta(elecTheta),
//  .mechOmega_PU(mechOmega_PU),
//  .elecTorque(elecTorque),
//  .elecTheta_PU(elecTheta_PU)
//
//  );

  pmsm_model_seq
  #(.n(`main_data_size), .m(`mult_data_size), .k(`qFormat_data_size
    ))
  dut_pmsm_model(
  .clk(clk),
  .rst(rst),
//  .en(en & ~do_cal),
  .en(en),
  .str(str_pmsm),
  .busy(busy_pmsm),
  .aV(aV),
  .bV(bV),
  .cV(cV),
  .mLoad(mLoad),
  .uI_pu(uI),
  .vI_pu(vI),
//  .wI(wI),
//  .elecTheta(elecTheta),
  .mechOmega_PU(mechOmega_PU),
```

```
//  .elecTorque_pu(elecTorque_pu),
  .elecTorque_pu(elecTorque),
  .elecTheta_PU(elecTheta_PU)

  );



endmodule
```

3 clkTrigger.v

This is a demo of the module clkTrigger.v and it is part of the Power Electronics Emulator project. This code is shared as a work-in-progress version due to the internal politics of our household Institute. The released version will be available upon request.

```
                          // +FHDR ------------------------------------------------------------------------
// Copyright (c) 2020 INAOE All rights reserved
// INSTITUTO NACIONAL DE ASTROF?SICA, OPTICA Y ELECTRONICA
// COORDINACI?N DE ELECTR?NICA
// GRUPO DE INSTRUMENTACI?N
// Confidential Proprietary
// -----------------------------------------------------------------------------
// FILE NAME  :   ph3_ab.v
// DEPARTMENT :   GRUPO DE SISTEMAS DIGITALES
// AUTHOR     :   Julio Hernandez
// AUTHOR?S EMAIL:  julio.hernandez@inaoep.mx
// -----------------------------------------------------------------------------
// RELEASE HISTORY
// VERSION DATE AUTHOR DESCRIPTION
//
// 1V0 200509 First test.
// -----------------------------------------------------------------------------
// PURPOSE  : This module implements a general trasformation from
//        three-phase coordinates frame (U,V,W) to stationary coordinates
//        frame (alpha-beta).
//        AKA Clark Transform.
// -FHDR ------------------------------------------------------------------------


module clkTrigger
#(  parameter   n = 8, trg_count_max= 100)
(
  input clk, rst, en,

  output reg trg
);

// -----------------------------------------------------------------------------
// Wire & Reg local definition

  // ph3 signal initialization
  reg [n-1:0] cnt;
```

```
  wire [n-1:0] maxCount;

// -----------------------------------------------------------------------------
// Top module assignations definition
  assign maxCount = trg_count_max[n-1:0];

// -----------------------------------------------------------------------------
// Top module assignations definition

  always @(posedge clk)
    begin
      if (rst)
        cnt <= 0;
      else
        begin
          if (en)
          begin
            if (cnt >= maxCount)
               cnt <= 0;
                       else
               cnt <= cnt + 1'b1;
          end
          else
            cnt <= 0;
        end
    end

  always @(posedge clk)
    begin
      if (rst)
        trg <= 0;
      else
        begin
          if (en)
          begin
            if (cnt >= maxCount)
               trg <= 1'b1;
                       else
               trg <= 0;
          end
          else
            trg <= 0;
        end
    end


endmodule
```

4 vsi_digModel.v

This is a demo of the module vsi_digModel.v and it is part of the Power Electronics Emulator project. This code is shared as a work-in-progress version due to the internal politics of our household Institute. The released version will be available upon request.

```
                        // +FHDR
    ------------------------------------------------------------------------
// Copyright (c) 2020 INAOE All rights reserved
// INSTITUTO NACIONAL DE ASTROF?SICA, OPTICA Y ELECTRONICA
// COORDINACI?N DE ELECTR?NICA
// GRUPO DE INSTRUMENTACI?N
// Confidential Proprietary
//
    -----------------------------------------------------------------------------
// FILE NAME  :   ph3_ab.v
// DEPARTMENT :   GRUPO DE SISTEMAS DIGITALES
// AUTHOR     :   Julio Hernandez
// AUTHOR?S EMAIL:  julio.hernandez@inaoep.mx
//
    -----------------------------------------------------------------------------
// RELEASE HISTORY
// VERSION DATE AUTHOR DESCRIPTION
//
// 1V0 200509 First test.
//
    -----------------------------------------------------------------------------
// PURPOSE  : This module implements a general trasformation from
//          three-phase coordinates frame (U,V,W) to stationary
    coordinates
//          frame (alpha-beta).
//          AKA Clark Transform.
// -FHDR
    ------------------------------------------------------------------------


module vsi_digModel
#(  parameter   n = 32)
(
  input clk, rst, en,

  input gateA_p, gateA_n, gateB_p, gateB_n, gateC_p, gateC_n,
  input signed [n-1:0] vdc,
  output reg signed [n-1:0] aV, bV, cV
);
//
    -----------------------------------------------------------------------------
// Top module assignations definition

  always @(posedge clk)
```

```
    begin
      if (rst)
        aV <= 0;
      else
        begin
          if (en)
          begin
            if (gateA_p > 0)
              aV <= vdc;
                       else
                if (gateA_n > 0)
                  aV <=-vdc;
                           else
                              aV <= 0;
          end
          else
            aV <= 0;
        end
    end

  always @(posedge clk)
    begin
      if (rst)
        bV <= 0;
      else
        begin
          if (en)
          begin
            if (gateB_p > 0)
              bV <= vdc;
                       else
                if (gateB_n > 0)
                  bV <=-vdc;
                           else
                              bV <= 0;
          end
          else
            bV <= 0;
        end
    end

  always @(posedge clk)
    begin
      if (rst)
        cV <= 0;
      else
        begin
          if (en)
          begin
            if (gateC_p > 0)
              cV <= vdc;
                       else
                if (gateC_n > 0)
                  cV <=-vdc;
                           else
                              cV <= 0;
          end
```

```
          else
            cV <= 0;
        end
    end

endmodule
```

5 pmsm_model_seq.v

This is a demo of the module pmsm_model_seq.v and it is part of the Power Electronics Emulator project. This code is shared as a work-in-progress version due to the internal politics of our household Institute. The released version will be available upon request.

```
//`timescale 1ns/100ps
// +FHDR ------------------------------------------------------------------------
// Copyright (c) 2020 INAOE All rights reserved
// INSTITUTO NACIONAL DE ASTROF?SICA, OPTICA Y ELECTRONICA
// COORDINACI?N DE ELECTR?NICA
// GRUPO DE SISTEMAS DIGITALES
// Confidential Proprietary
// -----------------------------------------------------------------------------
// FILE NAME  :   mcs_tb.v
// DEPARTMENT :   GRUPO DE SISTEMAS DIGITALES
// AUTHOR   :   Julio Hernandez
// AUTHOR?S EMAIL:    julio.hernandez@inaoep.mx
// -----------------------------------------------------------------------------
// RELEASE HISTORY
// VERSION DATE AUTHOR DESCRIPTION
//
// 1V0 220427 First test.
// -----------------------------------------------------------------------------
// PURPOSE  : This module implements a general purpose top level caller.
//        This module must be used as a bridge between Active-HDL and Quartus
// NOTE:    In Quartus the definicion of SYS_IMP must be activated.
// -----------------------------------------------------------------------------
// PROJECT PINOUT ASSIGMENT
//  WARNING: In order to use the DSG_DIG_2V0, dedicated pins must be configured as GPIO in Quartus
//        Go to: Assignments -> Device -> Device and Pin Options -> Dual-Purpose Pins, and change the value of the appropriate pin to Use as regular I/O.
//
// adc2dac.v    DSG_DIG_2V0   pinPlanner
// SPI_DIN      SPI_DIN     [7]
// SPI_DOUT     SPI_DOUT      [8]
// SPI_SCLK     SPI_SCLK      [6]
// ADC_CS_N     ADC_A_CS      [10]
// DAC_A_CS_N   DAC_AUX_CS    [11]  // This pin is used to drive the DAC_A or ADC_B on the DSG_DIG_2V0 board
// DAC_B_CS_N   DAC_B_CS      [13]
```

```
// FLASH_SC_N    B7_IO_44       [111] // This pin is used to drive
    the cs_n pin of the RAM or FLASH device on the DSG_DIG_2V0
    board
//



// -FHDR
    ------------------------------------------------------------------------

module pmsm_model_seq
#(  parameter    n = 32, m= 64, k=24, math_one_over_sqrt_three=
    9686330, Rs= 12582912, Ts= 17, phCurr_scale_pu = 8388608,
    elecTorque_scale_pu= 267153121)
(
  input clk, rst, en, str,
  output busy,

  input signed [n-1:0] aV, bV, cV, mLoad,
  output signed [n-1:0] elecTheta,
//  output signed [n-1:0] wI,
//  output signed [n-1:0] uI, vI, mechOmega_PU, elecTorque,
    elecTheta_PU
  output signed [n-1:0] uI_pu, vI_pu, mechOmega_PU, elecTorque_pu,
    elecTheta_PU

);

//
    -----------------------------------------------------------------------------

// Top module local parameters definition
  localparam[4:0]
    idle  = 5'b10000, // Idle
    stg0  = 5'b00000, //
    stg1  = 5'b00001, //
    stg2  = 5'b00010, //
    stg3  = 5'b00011, //
    stg4  = 5'b00100, //
    stg5  = 5'b00101, //
    stgX  = 5'b00110, //
    stgY  = 5'b00111, //
        stgZ    = 5'b01000, //
        stgA    = 5'b01001; //


  localparam [5:0]    // Control local parameters
    doNothing     = 6'b000000,  // Idle
    doPh3ab_CorSinCos = 6'b000001,  // Calculate the Clark
    trasformation and Cordic SinCos
    doab_dq       = 6'b000010,  // Calculate Park trasformation
    dodqFlux      = 6'b000100,  // Calculate dq Flux (voltage
    integration)
    dodqI       = 6'b001000,  // Calculate dqI
    doelecTorque_dqPh3  = 6'b010000,  // Calculate electromagnetic
    torque and three-phase currents
```

```
    domechModel     = 6'b100000;  // Calculate electromagnetic torque and three-phase currents

// -----------------------------------------------------------------------------
// Top module local reg definition
  // ctrl : Run the Counter process
  reg [5:0] ctrl;

  reg[4:0] state_p, state_f;

  wire [5:0] stgDone;

// -----------------------------------------------------------------------------
// Wire & Reg local definition

  // ph3 signal initialization
//  (* keep = "true" *) (* dont_touch = "true" *) wire signed [n-1:0] uV, vV, wV, alphaV, betaV;
  wire signed [n-1:0] uV, vV, wV, alphaV, betaV;
  wire str_ph3_ab, busy_ph3_ab;

  // ab_dq signal initialization
//  (* keep = "true" *) (* dont_touch = "true" *)
    wire signed [n-1:0] dV, qV;
  wire str_ab_dq_V, busy_ab_dq_V;

  // dqFlux signal initialization
//  (* keep = "true" *) (* dont_touch = "true" *)
  wire signed [n-1:0] dLamda, qLamda;
  wire str_dqFlux, busy_dFlux, busy_qFlux;

  // PMSM signal initialization
//  (* keep = "true" *) (* dont_touch = "true" *) wire signed [n-1:0] elecTheta_aux, elecOmega_noInertia, elecTorque_aux;
    wire signed [n-1:0] elecTheta_aux, elecOmega_noInertia, elecTorque_aux;
//  wire str_elecTorque, busy_elecTorque;

  // Cordic SinCos signal initialization
//  (* keep = "true" *) (* dont_touch = "true" *) wire signed [n-1:0] cosElecTheta, sinElecTheta;
    wire signed [n-1:0] cosElecTheta, sinElecTheta;
  wire str_corSinCos, busy_corSinCos;

  // dqI signal initialization
//  (* keep = "true" *) (* dont_touch = "true" *) wire signed [n-1:0] dI, qI;
    wire signed [n-1:0] dI, qI;
  wire str_dqI, busy_dqI;

  // dqI signal initialization
    wire signed [n-1:0] uI, vI;
  wire str_dq_ph3, busy_dq_ph3;
```

```
  wire str_mechModel_noInertia, busy_mechModel_noInertia;

  // PU signal initialization
//    wire signed [n-1:0] uI_pu, vI_pu, elecTorque_PU;
  wire str_pu;

//
    ---------------------------------------------------------------------------
// Top module initial conditions definition
  initial state_p = idle;
//
    ---------------------------------------------------------------------------
// Top module assignations definition
  assign busy = state_p[4];

  assign str_ph3_ab     = ctrl[0];
  assign str_corSinCos  = ctrl[0];
  assign str_ab_dq_V    = ctrl[1];
  assign str_dqFlux     = ctrl[2];
  assign str_dqI      = ctrl[3];
//  assign str_elecTorque   = ctrl[4];
  assign str_dq_ph3   = ctrl[4];
  assign str_mechModel_noInertia  = ctrl[5];
  assign str_pu     = ctrl[5];

  assign stgDone[0] = busy_ph3_ab & busy_corSinCos  ? 1'b1 : 1'b0;
  assign stgDone[1] = busy_ab_dq_V  ? 1'b1 : 1'b0;
  assign stgDone[2] = busy_dFlux & busy_qFlux ? 1'b1 : 1'b0;
  assign stgDone[3] = busy_dqI  ? 1'b1 : 1'b0;
//  assign stgDone[4] = busy_dq_ph3 & busy_elecTorque ? 1'b1 : 1'b0
    ;
  assign stgDone[4] = busy_dq_ph3 ? 1'b1 : 1'b0;
  assign stgDone[5] = busy_mechModel_noInertia;

  assign elecTheta= elecTheta_aux;
//  assign elecTorque= elecTorque_aux;

  // state machine activation
  always @(state_p or str or stgDone)
    begin
      state_f <= state_p;
      ctrl <= doNothing;

      case(state_p)
        idle :
        begin
          if(str)
          begin
            state_f <= stg0;
            ctrl <= doPh3ab_CorSinCos;
          end

        end

//        stg0 :
```

```
//         begin
//           if(stgDone[0])
//             begin
//               state_f <= stg1;
//               ctrl <= doab_dq;
//             end
//         end

        stg0 :
                begin
                    if(stgDone[0])
                        begin
                            state_f <= stgA;
                            ctrl <= doNothing;
                        end
                end

        stgA :
                begin
                    state_f <= stgX;
                    ctrl <= doNothing;
                end

        stgX :
                begin
                    state_f <= stgZ;
                    ctrl <= doNothing;
                end

        stgZ :
                begin
                    state_f <= stg1;
                    ctrl <= doab_dq;
                end

        stg1 :
        begin
          if(stgDone[1])
            begin
              state_f <= stg2;
              ctrl <= dodqFlux;
            end
        end

        stg2 :
        begin
          if(stgDone[2])
            begin
              state_f <= stg3;
              ctrl <= dodqI;
            end
        end

//         stg3 :
//         begin
//           if(stgDone[3])
//             begin
```

```
//                state_f <= stg4;
//                ctrl <= doelecTorque_dqPh3;
//              end
//          end

        stg3 :
                begin
                    if(stgDone[0])
                        begin
                            state_f <= stgY;
                            ctrl <= doNothing;
                        end
                end

        stgY :
                begin
                    state_f <= stg4;
                    ctrl <= doelecTorque_dqPh3;
                end

        stg4 :
        begin
          if(stgDone[4])
            begin
              state_f <= stg5;
              ctrl <= domechModel;
            end
        end

        stg5 :
        begin
          if(stgDone[3])
            begin
              state_f <= idle;
              ctrl <= doNothing;
            end
        end

        default :
          begin
            ctrl <= doNothing;
            state_f <= idle;
          end
      endcase
    end

  always @(posedge clk)
    begin
      if (rst)
        state_p <= idle;
      else
        state_p <= state_f;
    end

//  (* DONT_TOUCH = "TRUE" *)
    ph3_ab
```

```
  #(.n(n), .m(m), .k(k), .math_one_over_sqrt_three(
    math_one_over_sqrt_three) )
  dut_ph3_ab(
  .clk(clk),
  .rst(rst),
  .en(en),
  .str(str_ph3_ab),
  .busy(busy_ph3_ab),

  .uX(aV),
  .vX(bV),
  .wX(cV),
  .alphaX(alphaV),
  .betaX(betaV)
  );

//  (* DONT_TOUCH = "TRUE" *) cordic_sincos_hdr
    cordic_sincos_hdr
  dut_cor_sincos(
  .clk(clk),
  .rst(rst),
  .str(str_corSinCos),
  .busy(busy_corSinCos),
  .angle(elecTheta_aux),
  .cos (cosElecTheta),
  .sin (sinElecTheta)
  );

//  (* DONT_TOUCH = "TRUE" *) ab_dq
//    ab_dq
//  #(.n(n), .m(m), .k(k))
//  dut_ab_dq(
//  .clk(clk),
//  .rst(rst),
//  .en(en),
//  .str(str_ab_dq_V),
//  .busy(busy_ab_dq_V),
//  .alphaX(alphaV),
//  .betaX(betaV),
//  .cosElecTheta(cosElecTheta),
//  .sinElecTheta(sinElecTheta),
//  .dX(dV),
//  .qX(qV)

//  );

    abV_dqV_seq
  #(.n(n), .m(m), .k(k))
  dut_ab_dq(
  .clk(clk),
  .rst(rst),
  .en(en),
  .str(str_ab_dq_V),
  .busy(busy_ab_dq_V),
  .alphaV(alphaV),
  .betaV(betaV),
  .cosElecTheta(cosElecTheta),
```

```
  .sinElecTheta(sinElecTheta),
  .dV(dV),
  .qV(qV)

  );

////  (* DONT_TOUCH = "TRUE" *)
//    dFlux
//  #(.n(n), .m(m), .k(k), .Rs(12582912), .Ts(Ts))
//  dut_dFlux(
//  .clk(clk),
//  .rst(rst),
//  .en(en),
//  .str(str_dqFlux),
//  .busy(busy_dFlux),
//  .dV(dV),
//  .dI(dI),
//  .qLamda(qLamda),
//  .elecOmega(elecOmega_noInertia),
//  .dLamda(dLamda)

//  );

////  (* DONT_TOUCH = "TRUE" *) qFlux
//    qFlux
//  #(.n(n), .m(m), .k(k), .Rs(12582912), .Ts(Ts))
//  dut_qFlux(
//  .clk(clk),
//  .rst(rst),
//  .en(en),
//  .str(str_dqFlux),
//  .busy(busy_qFlux),
//  .qV(qV),
//  .qI(qI),
//  .dLamda(dLamda),
//  .elecOmega(elecOmega_noInertia),
//  .qLamda(qLamda)

//  );

//  (* DONT_TOUCH = "TRUE" *)
    dFlux_seq
  #(.n(n), .m(m), .k(k), .Rs(12582912), .Ts(Ts))
  dut_dFlux(
  .clk(clk),
  .rst(rst),
  .en(en),
  .str(str_dqFlux),
  .busy(busy_dFlux),
  .dqV(dV),
  .dqI(dI),
  .dqFlux(qLamda),
  .elecOmega(elecOmega_noInertia),
  .dFlux(dLamda)

  );

```

```
//  (* DONT_TOUCH = "TRUE" *) qFlux
    qFlux_seq
  #(.n(n), .m(m), .k(k), .Rs(12582912), .Ts(Ts))
  dut_qFlux(
  .clk(clk),
  .rst(rst),
  .en(en),
  .str(str_dqFlux),
  .busy(busy_qFlux),
  .dqV(qV),
  .dqI(qI),
  .dqFlux(dLamda),
  .elecOmega(elecOmega_noInertia),
  .qFlux(qLamda)

  );

////  (* DONT_TOUCH = "TRUE" *) dqI
//    dqI
//  #(.n(n), .m(m), .k(k), .pmsm_pm(85832), .dL_factor(15603809), .qL_factor(15603809), .dqL_shift_bit(10))
//  dut_dqI(
//  .clk(clk),
//  .rst(rst),
//  .en(en),
//  .str(str_dqI),
//  .busy(busy_dqI),
//  .dLamda(dLamda),
//  .qLamda(qLamda),
//  .dI(dI),
//  .qI(qI)

//  );

////  (* DONT_TOUCH = "TRUE" *) elecTorque
//    elecTorque
//  #(.n(n), .m(m), .k(k), .pmsm_three_over_two_n(100663296))
//  dut_elecTorque(
//  .clk(clk),
//  .rst(rst),
//  .en(en),
//  .str(str_elecTorque),
//  .busy(busy_elecTorque),
//  .dLamda(dLamda),
//  .qLamda(qLamda),
//  .dI(dI),
//  .qI(qI),
//  .elecTorque(elecTorque_aux)

//  );

//#(  parameter   n = 32, m= 64, k=24, j=3, pmsm_pm= 85832, dqL_shift_bit= 10, qdL_factor= 15603809, pmsm_pp_three_over_two = 100663296)
    dqI_elecTorque_seq
  #(.n(n), .m(m), .k(k), .pmsm_pm(85832), .qdL_factor(15603809), .dqL_shift_bit(10))
```

```
  dut_dqI(
  .clk(clk),
  .rst(rst),
  .en(en),
  .str(str_dqI),
  .busy(busy_dqI),
  .dFlux(dLamda),
  .qFlux(qLamda),
  .dI(dI),
  .qI(qI),
  .elecTorque(elecTorque_aux)

  );

//  (* DONT_TOUCH = "TRUE" *) dq_ph3
//     dq_ph3
//  #(.n(n), .m(m), .k(k), .math_sqrt_three_over_two(14529495))
//  dut_dq_ph3(
//  .clk(clk),
//  .rst(rst),
//  .en(en),
//  .str(str_dq_ph3),
//  .busy(busy_dq_ph3),
//  .dI(dI),
//  .qI(qI),
//  .sinElecTheta(sinElecTheta),
//  .cosElecTheta(cosElecTheta),
////  .wI(wI),
//  .vI(vI),
//  .uI(uI)

//  );

    dqI_ph3_seq
  #(.n(n), .m(m), .k(k), .math_sqrt_three_over_two(14529495))
  dut_dq_ph3(
  .clk(clk),
  .rst(rst),
  .en(en),
  .str(str_dq_ph3),
  .busy(busy_dq_ph3),
  .dI(dI),
  .qI(qI),
  .sinElecTheta(sinElecTheta),
  .cosElecTheta(cosElecTheta),
//  .wI(wI),
  .vI(vI),
  .uI(uI)

  );

////  (* DONT_TOUCH = "TRUE" *)
//     mechOmega_elecTheta_elecOmega_noInertia
//  #(.n(n), .m(m), .k(k), .pmsm_dryFriction(0), .pmsm_visFriction
    (0), .spdPU_factor(32582714), .spd_shift_bit(9), .j_factor
    (13328335), .j_shift_bit(19), .pmsm_n(67108864), .math_two_pi
    (105414357), .Ts(Ts))
```

```
//   dut_mechOmega_elecTheta_elecOmega_noInertia(
//   .clk(clk),
//   .rst(rst),
//   .en(en),
//   .str(str_mechModel_noInertia),
//   .busy(busy_mechModel_noInertia),
//   .elecTorque(elecTorque_aux),
//   .mLoad(mLoad),
//   .elecTheta(elecTheta_aux),
//   .mechOmega_PU(mechOmega_PU),
//   .elecOmega_noInertia(elecOmega_noInertia),
//   .elecTheta_PU(elecTheta_PU)

//   );

//   (* DONT_TOUCH = "TRUE" *)
    mechOmega_elecTheta_elecOmega_noInertia_seq
  #(.n(n), .m(m), .k(k), .pmsm_dryFriction(0), .pmsm_visFriction(0)
    , .spdPU_factor(32582714), .spd_shift_bit(9), .j_factor
    (13328335), .j_shift_bit(19), .pmsm_n(67108864), .math_two_pi
    (105414357), .Ts(Ts))
  dut_mechOmega_elecTheta_elecOmega_noInertia(
  .clk(clk),
  .rst(rst),
  .en(en),
  .str(str_mechModel_noInertia),
  .busy(busy_mechModel_noInertia),
  .elecTorque(elecTorque_aux),
  .mLoad(mLoad),
  .elecTheta(elecTheta_aux),
  .mechOmega_PU(mechOmega_PU),
  .elecOmega_noInertia(elecOmega_noInertia),
  .elecTheta_PU(elecTheta_PU)

  );


    signal_pu
        #(.n(n), .m(m), .k(k))
        dut_signal_pu(
        .clk(clk),
        .rst(rst),
        .en(en),
    .str(str_pu),

    .mult_a1(uI),
        .mult_b1(phCurr_scale_pu),
        .mult_out1(uI_pu),

    .mult_a2(vI),
        .mult_b2(phCurr_scale_pu),
        .mult_out2(vI_pu),

    .mult_a3(elecTorque_aux),
        .mult_b3(elecTorque_scale_pu),
        .mult_out3(elecTorque_pu)

```

```
        );


endmodule
```

6 ph3_ab.v

This is a demo of the module ph3_ab.v and it is part of the Power Electronics Emulator project. This code is shared as a work-in-progress version due to the internal politics of our household Institute. The released version will be available upon request.

```
// +FHDR
    ------------------------------------------------------------------------

// Copyright (c) 2020 INAOE All rights reserved
// INSTITUTO NACIONAL DE ASTROF?SICA, OPTICA Y ELECTRONICA
// COORDINACI?N DE ELECTR?NICA
// GRUPO DE INSTRUMENTACI?N
// Confidential Proprietary
//
    -----------------------------------------------------------------------------

// FILE NAME  :   ph3_ab.v
// DEPARTMENT :   GRUPO DE SISTEMAS DIGITALES
// AUTHOR   :   Julio Hernandez
// AUTHOR?S EMAIL:  julio.hernandez@inaoep.mx
//
    -----------------------------------------------------------------------------

// RELEASE HISTORY
// VERSION DATE AUTHOR DESCRIPTION
//
// 1V0 200509 First test.
//
    -----------------------------------------------------------------------------

// PURPOSE  : This module implements a general trasformation from
//        three-phase coordinates frame (U,V,W) to stationary
    coordinates
//        frame (alpha-beta).
//        AKA Clark Transform.
// -FHDR
    ------------------------------------------------------------------------



module ph3_ab
#(  parameter   n = 32, m= 64, k=24, math_one_over_sqrt_three=
    9686330)
(
  input clk, rst, en, str,
  output busy,

  input signed [n-1:0] uX, vX, wX,
  output reg signed [n-1:0] alphaX,
  output signed [n-1:0] betaX
);
//
    -----------------------------------------------------------------------------

// Top module local parameters definition
```

```
  localparam //[1:0]
    idle  = 1'b1, // Idle
    stg0  = 1'b0; // Run the algorithm


  localparam [1:0]    // Control local parameters
  doNothing = 2'b00,  // Idle
  doAdder1  = 2'b01,  // Calculate vX - wX
  doMult1   = 2'b10;  // Calculate betaX= 1/sqrt(3)*(vX - wX)
    alphaX= uX

//
    ----------------------------------------------------------------------------
// Top module local reg definition
  // ctrl : Run the Counter process
  reg [1:0] ctrl;

//  reg[1:0] state_p, state_f;
  reg state_p, state_f;
//
    ----------------------------------------------------------------------------
// Wire & Reg local definition

  wire signed   [n-1:0] vXwX;
  wire signed   [n-1:0] cnt_vXwX;

//
    ----------------------------------------------------------------------------
// Top module initial conditions definition
  initial state_p = idle;
//
    ----------------------------------------------------------------------------
// Top module assignations definition
//  assign busy = state_p[1];
  assign busy = state_p;

  // state machine activation
  always @(state_p or str)
    begin
      state_f <= state_p;
      case(state_p)
        idle :
        begin
          if(str)
          begin
            state_f <= stg0;
            ctrl <= doAdder1;
          end
          else
            ctrl <= doNothing;
        end

        stg0 :
```

```
      begin
        state_f <= idle;
        ctrl <= doMult1;
      end

      default :
        begin
          ctrl <= doNothing;
          state_f <= idle;
        end
    endcase
  end

always @(posedge clk)
  begin
    if (rst)
      state_p <= idle;
    else
      state_p <= state_f;
  end

// outBlk activation
always@(posedge clk)
  begin
    if(rst)
      alphaX <= 0;
    else
      begin
        if (en)
          begin
            if (ctrl[1])
              begin
                alphaX <= uX;
              end
          end
        else
          begin
            alphaX <= 0;
          end
      end
  end

addsub #( .n(n))
dut1
(
  .clk(clk),
  .rst(rst),
  .en(en),
  .str(ctrl[0]),
  .iVal_a( vX),
  .iVal_b(-wX),
  .oVal_z(vXwX)
);

fix_sig_mult_nbit #(.n(n), .m(m), .k(k))
dut2
(
```

```
    .clk(clk),
    .rst(rst),
    .en(en),
    .str(ctrl[1]),
    .mult_a(vXwX),
    .mult_b(math_one_over_sqrt_three),
    .mult_out(betaX)
  );


endmodule
```

7 cordic_sincos_hdr.v

This is a demo of the module cordic_sincos_hdr.v and it is part of the Power Electronics Emulator project. This code is shared as a work-in-progress version due to the internal politics of our household Institute. The released version will be available upon request.

```
// +FHDR ------------------------------------------------------------------------
// Copyright (c) 2020 INAOE All rights reserved
// INSTITUTO NACIONAL DE ASTROF?SICA, OPTICA Y ELECTRONICA
// COORDINACI?N DE ELECTR?NICA
// GRUPO DE INSTRUMENTACI?N
// Confidential Proprietary
// -----------------------------------------------------------------------------
// DEPARTMENT : LABORATORIO DE SISTEMAS DIGITALES
// AUTHOR   : Julio Hernandez
// A U T H O R S  EMAIL:  julio.hernandez@inaoep.mx
// -----------------------------------------------------------------------------
// RELEASE HISTORY
// VERSION DATE AUTHOR DESCRIPTION
//
// 1V0 200217   First test. This module is based on "Use Cordic to Calculate Sin/Cos with Verilog Implementation" project.
//        For more information, see https://www.valpont.com/use-cordic-to-calculate-sincos-with-verilog-implementation/pst/.
//        The input in rads is obtained and evaluated such a way 0-pi/2 and polarity expected is always send to cordic_core and cordic_rom_X_Y file.
// 1V1 200219   This module is renamed to cordic_sincos_hdr based on cordic arctan generation aproach.
//        The internal registers are renamed to sincos_core_XYZ based on cordic arctan generation aproach.
// -----------------------------------------------------------------------------
// PURPOSE  : This module handles the cordic algorithm to generate a sin/cos output.
//        The algorithm computes a 0-pi/2 rad input and sin/cos is calculated as 0-1 output; the Q30 format is used.
// -----------------------------------------------------------------------------
// NOTE :   Warning! The properties assigned to this module are set  assuming a resolution of 32bit.
//        If different resolution is required, module parameters and cordic_rom_X_Y must be updated as well.
// -FHDR ------------------------------------------------------------------------

//`timescale 1ns/100ps
```

```
`define CORDIC_DATA_WIDTH   32
`define CORDIC_ADDR_WIDTH   5
`define CORDIC_GAIN         32'h26DD3B6A
`define INTERNAL_QFORMAT    30         // Q30
`define EXTERNAL_QFORMAT    24         // Q24
`define ZERO_Q24          32'h00000000
`define ONE_Q24           32'h01000000
`define PI_OVER_TWO_Q24      32'h01921FB5  // PI/2 = 1.5707
`define PI_Q24            32'h03243F6A  // PI   = 3.1415
`define THREE_PI_OVER_TWO_Q24 32'h04B65F1F  // 3*PI/2 = 4.7123
`define TWO_PI_Q24          32'h06487ED4  // 2*PI  = 6.2831

module cordic_sincos_hdr //#( parameter  DATA_WIDTH = 32,
    ADDR_WIDTH = 5, IQ = 24)
(
  input  clk,rst,str,
  output wire busy,
  input  signed [`CORDIC_DATA_WIDTH-1:0] angle,
  output wire signed [`CORDIC_DATA_WIDTH-1:0] cos, sin
);

  wire [`CORDIC_ADDR_WIDTH-1:0] rom_addr;
  wire [`CORDIC_DATA_WIDTH-1:0] rom_data;
  reg signed [`CORDIC_DATA_WIDTH-1:0] sincos_core_angle;
  wire signed [`CORDIC_DATA_WIDTH-1:0] sincos_core_cos;
  wire signed [`CORDIC_DATA_WIDTH-1:0] sincos_core_sin;

  reg signed [`CORDIC_DATA_WIDTH-1:0] preCos;
  reg signed [`CORDIC_DATA_WIDTH-1:0] preSin;

  wire sincos_core_done;
  wire[1:0] ctrl;
  reg pol;    // Defines the polarization of sin/cos output


  // ctrl[0] : The start flag for cordic_core module
  // ctrl[1] : The cordic_core module status
  localparam[1:0]
    idle   = 2'b10,  // Idle
    start   = 2'b01,  // Start the cordic_core module
    run    = 2'b00;  // The cordic_core module is running

  reg[1:0] Qp, Qf;

  initial Qp = idle;
  assign ctrl = Qp;
  assign busy = ctrl[1];

  assign cos= preCos;
  assign sin= preSin;

  always @(posedge clk)
  begin
   if(rst)
     begin
      preCos    <= `ONE_Q24;
```

```
    preSin     <= ‘ZERO_Q24;
    pol      <= 1’b1;
  end
 else
   begin
     if (angle >= ‘THREE_PI_OVER_TWO_Q24 && angle < ‘TWO_PI_Q24)
       begin
         sincos_core_angle <= (angle -‘TWO_PI_Q24) << (‘INTERNAL_QFORMAT-‘EXTERNAL_QFORMAT);
         pol     <= 1’b1;
       end
     else
       begin
         if (angle >= ‘PI_OVER_TWO_Q24 && angle < ‘THREE_PI_OVER_TWO_Q24)
           begin
             sincos_core_angle <= (angle -‘PI_Q24) << (‘INTERNAL_QFORMAT-‘EXTERNAL_QFORMAT);
             pol     <= 1’b0;
           end
         else
           begin
             sincos_core_angle <= angle << (‘INTERNAL_QFORMAT-‘EXTERNAL_QFORMAT);
             pol     <= 1’b1;
           end
       end

     if (sincos_core_done)
     begin
      preCos     <= {{‘INTERNAL_QFORMAT-‘EXTERNAL_QFORMAT{sincos_core_cos[‘CORDIC_DATA_WIDTH-1]}}, sincos_core_cos [‘CORDIC_DATA_WIDTH-1:‘INTERNAL_QFORMAT-‘EXTERNAL_QFORMAT]};
      preSin     <= {{‘INTERNAL_QFORMAT-‘EXTERNAL_QFORMAT{sincos_core_sin[‘CORDIC_DATA_WIDTH-1]}}, sincos_core_sin [‘CORDIC_DATA_WIDTH-1:‘INTERNAL_QFORMAT-‘EXTERNAL_QFORMAT]};

     end
   end
end

always @(Qp or str or sincos_core_done)
  begin
    case(Qp)
      idle :
        if(str)
          Qf <= start;
        else
          Qf <= Qp;
      start :
        Qf <= run;
      run :
        if(sincos_core_done)
          Qf <= idle;
        else
          Qf <= Qp;
      default :
```

```
          Qf <= idle;
      endcase
    end
  always @(posedge clk)
    begin
      if(rst)
        Qp <= idle;
      else //if(ena)
        Qp <= Qf;
    end

  cordic_sincos_core #(.DATA_WIDTH(`CORDIC_DATA_WIDTH),.ADDR_WIDTH(
    `CORDIC_ADDR_WIDTH), .GAIN(`CORDIC_GAIN))
  core
  (
    .clk(clk),
    .rst(rst),
    .str(ctrl[0]),
    .pol(pol),
    .done(sincos_core_done),
    .angle(sincos_core_angle),
    .addr(rom_addr),
    .data(rom_data),
    .cos(sincos_core_cos),
    .sin(sincos_core_sin)
  );

  cordic_rom_32_5 #(.DATA_WIDTH(`CORDIC_DATA_WIDTH), .ADDR_WIDTH(
    `CORDIC_ADDR_WIDTH))
  rom_32_5
  (
    .addr(rom_addr),
    .data(rom_data)
  );

endmodule
```

8 cordic_sincos_core.v

This is a demo of the module cordic_sincos_core.v and it is part of the Power Electronics Emulator project. This code is shared as a work-in-progress version due to the internal politics of our household Institute. The released version will be available upon request.

```
// +FHDR ------------------------------------------------------------------------
// Copyright (c) 2020 INAOE All rights reserved
// INSTITUTO NACIONAL DE ASTROF?SICA, OPTICA Y ELECTRONICA
// COORDINACI?N DE ELECTR?NICA
// GRUPO DE INSTRUMENTACI?N
// Confidential Proprietary
// ----------------------------------------------------------------------------
// DEPARTMENT : LABORATORIO DE SISTEMAS DIGITALES
// AUTHOR   : Julio Hernandez
//  A U T H O R S  EMAIL:  julio.hernandez@inaoep.mx
// ----------------------------------------------------------------------------
// RELEASE HISTORY
// VERSION DATE AUTHOR DESCRIPTION
//
// 1V0 200217   First test. This module is based on "Use Cordic to Calculate Sin/Cos with Verilog Implementation" project.
//         For more information, see https://www.valpont.com/use-cordic-to-calculate-sincos-with-verilog-implementation/pst/.
//         The input gain parameter is used to calculate the cordic algorithim and is based on the data width parameter.
//         The output is a 0-1 value with fixed point on data_width-2 QFormat
// 1V1 200219   This module is renamed to cordic_sincos_core based on cordic arctan generation aproach.
// ----------------------------------------------------------------------------
// PURPOSE  : This module sets a constants LUT required to compute the cordic algorithm.
// ----------------------------------------------------------------------------
// SYNCHRONOUS MODULE : Yes.
// ----------------------------------------------------------------------------
// NOTE :
// -FHDR ------------------------------------------------------------------------

module cordic_sincos_core #(parameter DATA_WIDTH = 16, ADDR_WIDTH = 4, GAIN = 16'h26dd )
(
```

```
  input clk, rst, str, pol,
  input signed [DATA_WIDTH-1:0] angle,data,
  output [ADDR_WIDTH-1:0] addr,
  output done,
  output signed [DATA_WIDTH-1:0] cos,sin
);
  reg [ADDR_WIDTH-1:0] n,n_next;
  reg done_int, done_dly;
  wire done_redge;

  reg signed [DATA_WIDTH+3:0]x_reg,y_reg,x_next,y_next,z_reg,z_next;


  reg [1:0] state,state_next;

  localparam idle = 1'b0, iteration = 1'b1;

  always @ (posedge clk, posedge rst)
  begin
    if (rst)
    begin
      state <= idle;
      n     <= 0;
      x_reg <= 0;
      y_reg <= 0;
      z_reg <= 0;
      done_dly <=1;
    end
    else
    begin
      state <= state_next;
      n     <= n_next;
      x_reg <= x_next;
      y_reg <= y_next;
      z_reg <= z_next;
      done_dly <= done_int;
    end
  end


  always @ (*)
  begin
//    n_next = n;
//    done_int = 1'b0;
    case (state)
      idle:
      begin
        if (str)
        begin
          state_next = iteration;
        end
        else
        begin
          state_next = idle;
        end
        n_next = 0;
```

```
        done_int = 1'b1;
      end
      iteration:
      begin
        if (n == (DATA_WIDTH-2))
        begin
//            n_next = n;
          state_next = idle;
        end
        else
        begin
//          n_next = n + 1'b1;
          state_next = iteration;
        end
        n_next = n + 1'b1;
        done_int = 1'b0;
      end
    endcase
  end


  always @ (*)
  begin
    case (state)
      idle:
      begin
        x_next = GAIN;
        y_next = 0;
        z_next = angle;

      end
      iteration:
      begin
        if (z_reg[DATA_WIDTH-1]==1'b0)      //check polarity
        begin
          x_next = x_reg - (y_reg >>> n);
          y_next = y_reg + (x_reg >>> n);
          z_next = z_reg - data;
        end
        else
        begin
          x_next = x_reg + (y_reg >>> n);
          y_next = y_reg - (x_reg >>> n);
          z_next = z_reg + data;
        end
      end
    endcase
  end


  assign addr = n;
  assign done = done_redge;
  assign done_redge = done_int & (~done_dly);

  assign cos = done_redge? (pol? x_reg:-x_reg): 0;
  assign sin = done_redge? (pol? y_reg:-y_reg): 0;

```

```
endmodule
```

9 cordic_rom_32_5.v

This is a demo of the module cordic_rom_32_5.v and it is part of the Power Electronics Emulator project. This code is shared as a work-in-progress version due to the internal politics of our household Institute. The released version will be available upon request.

```
// +FHDR ------------------------------------------------------------------------
// Copyright (c) 2020 INAOE All rights reserved
// INSTITUTO NACIONAL DE ASTROF?SICA, OPTICA Y ELECTRONICA
// COORDINACI?N DE ELECTR?NICA
// GRUPO DE INSTRUMENTACI?N
// Confidential Proprietary
// -----------------------------------------------------------------------------
// DEPARTMENT : LABORATORIO DE SISTEMAS DIGITALES
// AUTHOR   : Julio Hernandez
// AUTHORS EMAIL:  julio.hernandez@inaoep.mx
// -----------------------------------------------------------------------------
// RELEASE HISTORY
// VERSION DATE AUTHOR DESCRIPTION
//
// 1V0 200217   First test. This module is based on "Use Cordic to Calculate Sin/Cos with Verilog Implementation" project.
//        For more information, see https://www.valpont.com/use-cordic-to-calculate-sincos-with-verilog-implementation/pst/.
//        The input address required is evaluated and the value is send to the output.
// -----------------------------------------------------------------------------
// PURPOSE  : This module sets a constants LUT required to compute the cordic algorithm.
// -----------------------------------------------------------------------------
// SYNCHRONOUS MODULE : No.
// -----------------------------------------------------------------------------
// NOTE :   Warning! The properties assigned to this module are set  assuming a resolution of 32bit.
//        If different resolution is required, module parameters and cordic_rom_X_Y must be updated as well.
// -FHDR ------------------------------------------------------------------------


// Async Single Port ROM

module cordic_rom_32_5 #( parameter  DATA_WIDTH = 32, ADDR_WIDTH = 5)
```

```
(
  input  [(ADDR_WIDTH-1):0]  addr,
  output [(DATA_WIDTH-1):0]  data

);

//  localparam [(ADDR_WIDTH-1):0]
//  gain    = 32'h26DD3B6A;         // cordic_core output polatity 1= positive; 0= negative

  // Declare the ROM variable//
  // Data used on 32bit
  // Gain = 32'h26dd3b6a;

  reg [DATA_WIDTH-1:0] rom[(2**ADDR_WIDTH)-1:0];
  initial
  begin
    rom[0]= 32'h3243F6A9;
    rom[1]= 32'h1DAC6705;
    rom[2]= 32'h0FADBAFD;
    rom[3]= 32'h07F56EA7;
    rom[4]= 32'h03FEAB77;
    rom[5]= 32'h01FFD55C;
    rom[6]= 32'h00FFFAAB;
    rom[7]= 32'h007FFF55;
    rom[8]= 32'h003FFFEB;
    rom[9]= 32'h001FFFFD;
    rom[10]= 32'h00100000;
    rom[11]= 32'h00080000;
    rom[12]= 32'h00040000;
    rom[13]= 32'h00020000;
    rom[14]= 32'h00010000;
    rom[15]= 32'h00008000;
    rom[16]= 32'h00004000;
    rom[17]= 32'h00002000;
    rom[18]= 32'h00001000;
    rom[19]= 32'h00000800;
    rom[20]= 32'h00000400;
    rom[21]= 32'h00000200;
    rom[22]= 32'h00000100;
    rom[23]= 32'h00000080;
    rom[24]= 32'h00000040;
    rom[25]= 32'h00000020;
    rom[26]= 32'h00000010;
    rom[27]= 32'h00000008;
    rom[28]= 32'h00000004;
    rom[29]= 32'h00000002;
    rom[30]= 32'h00000001;
    rom[31]= 32'h00000000;
  end

  assign data = rom[addr];

endmodule
```

10 abV_dqV_seq.v

This is a demo of the module abV_dqV_seq.v and it is part of the Power Electronics Emulator project. This code is shared as a work-in-progress version due to the internal politics of our household Institute. The released version will be available upon request.

```
`timescale 1ns/100ps
// +FHDR
    ------------------------------------------------------------------------

// Copyright (c) 2020 INAOE All rights reserved
// INSTITUTO NACIONAL DE ASTROF?SICA, OPTICA Y ELECTRONICA
// COORDINACI?N DE ELECTR?NICA
// GRUPO DE INSTRUMENTACI?N
// Confidential Proprietary
//
    -----------------------------------------------------------------------------

// FILE NAME  :   svm_gen.v
// DEPARTMENT :   GRUPO DE SISTEMAS DIGITALES
// AUTHOR   :   Julio Hernandez
// AUTHOR?S EMAIL:  julio.hernandez@inaoep.mx
//
    -----------------------------------------------------------------------------

// RELEASE HISTORY
// VERSION DATE AUTHOR DESCRIPTION
//
// 1V0 200509 First test.
//
    -----------------------------------------------------------------------------

// PURPOSE  : This module implements a space vector modulation
    generator.
// -FHDR
    ------------------------------------------------------------------------



module abV_dqV_seq
#(  parameter   n = 32, m= 64, k=24, j=3 )
(
  input clk, rst, en, str,
  output busy,

  input signed [n-1:0] alphaV, betaV, cosElecTheta, sinElecTheta,

  output reg signed [n-1:0] dV, qV
);
//
    -----------------------------------------------------------------------------

// Top module local parameters definition
  localparam[5:0]
  idle  = 6'b100000,    // Idle
  stg0  = 6'b000000,    //
```

```
  stg1  = 6'b000001,    //
  stg2  = 6'b000010,    //
  stg3  = 6'b000011,    //
  stg4  = 6'b000100,    //
  stg5  = 6'b000101,    //
  stg6  = 6'b000110,    //
  stg7  = 6'b000111,    //
  stg8  = 6'b001000,    //
  stg9  = 6'b001001,    //
  stg10   = 6'b001010,    //
  stg11   = 6'b001011,    //
  stg12   = 6'b001100,    //
  stg13 = 6'b001101,    //
  stg14   = 6'b001110,    //
  stg15   = 6'b001111,    //
  stg16 = 6'b010000;    //

  localparam [6:0]    // Control local parameters
  doNothing = 7'b0000000, //
  doLoad0   = 7'b0001000, //
  doLoad1   = 7'b0001001, //
  doLoad2   = 7'b0001010, //
  doLoad3   = 7'b0001011, //
  doLoad4   = 7'b0001100, //
  doLoad5   = 7'b0001101, //

  doSave0   = 7'b0010000, //
  doSave1   = 7'b0010001, //
  doSave2   = 7'b0010010, //
  doSave3   = 7'b0010011, //
  doSave4   = 7'b0010100, //
  doSave5   = 7'b0010101, //

  doGenMult = 7'b0100000, //
  doGenAdd  = 7'b1000000; //

  localparam [2:0]    // id values
  id_0  = 3'd0, //
  id_1  = 3'd1, //
  id_2  = 3'd2, //
  id_3  = 3'd3, //
  id_4  = 3'd4, //
  id_5  = 3'd5; //


//
    ----------------------------------------------------------------------------
// Top module local reg definition
  // ctrl : Run the Counter process
  reg [6:0] ctrl;

  reg [5:0] state_p, state_f;

//
    ----------------------------------------------------------------------------
```

```
// Wire & Reg local definition

  wire      [j-1:0] id;

  reg signed    [n-1:0] inMult_a;
  reg signed    [n-1:0] inMult_b;
  reg signed    [n-1:0] inAdd_a;
  reg signed    [n-1:0] inAdd_b;

  wire signed   [n-1:0] res_mult;
  wire signed   [n-1:0] res_add;


  reg signed    [n-1:0] alphaV_cosElecTheta,  betaV_sinElecTheta;
  reg signed    [n-1:0]  betaV_cosElecTheta, alphaV_sinElecTheta;

  wire str_load, str_mult, str_add;
  wire str_save0, str_save1, str_save2, str_save3, str_save4,
    str_save5;

//
    ----------------------------------------------------------------------------

// Top module assignations definition
  assign busy = state_p[5];

  assign id           = {ctrl[2],ctrl[1],ctrl[0]};

  assign str_load   = ctrl[3];
  assign str_mult   = ctrl[5];
  assign str_add    = ctrl[6];

  assign str_save0  = (ctrl[4] & ~ctrl[2] & ~ctrl[1] & ~ctrl[0]) ?
    1'b1 : 1'b0;
  assign str_save1  = (ctrl[4] & ~ctrl[2] & ~ctrl[1] &  ctrl[0]) ?
    1'b1 : 1'b0;
  assign str_save2  = (ctrl[4] & ~ctrl[2] &  ctrl[1] & ~ctrl[0]) ?
    1'b1 : 1'b0;
  assign str_save3  = (ctrl[4] & ~ctrl[2] &  ctrl[1] &  ctrl[0]) ?
    1'b1 : 1'b0;
  assign str_save4  = (ctrl[4] &  ctrl[2] & ~ctrl[1] & ~ctrl[0]) ?
    1'b1 : 1'b0;
  assign str_save5  = (ctrl[4] &  ctrl[2] & ~ctrl[1] &  ctrl[0]) ?
    1'b1 : 1'b0;

  // state machine activation
  always @(state_p or str)
    begin
      state_f <= state_p;
      ctrl  <= doNothing;

      case(state_p)
        idle :
        begin
          if (str)
            begin
              state_f <=stg0;
```

```
          ctrl <= doLoad0;
        end
    end

    stg0 :
    begin
      state_f <= stg1;
      ctrl <= doGenMult; // alphaV * cosElecTheta
    end

    stg1 :
    begin
      state_f <= stg2;
      ctrl <= doSave0;
    end

    stg2 :
    begin
      state_f <= stg3;
      ctrl <= doLoad1;
    end

    stg3 :
    begin
      state_f <= stg4;
      ctrl <= doGenMult; //  betaV * sinElecTheta
    end

    stg4 :
    begin
      state_f <= stg5;
      ctrl <= doSave1;
    end

    stg5 :
    begin
      state_f <= stg6;
      ctrl <= doLoad2;
    end

    stg6 :
    begin
      state_f <= stg7;
      ctrl <= doGenAdd; //  alphaV_cosElecTheta +
betaV_sinElecTheta
    end

    stg7 :
    begin
      state_f <= stg8;
      ctrl <= doSave2;
    end

    stg8 :
    begin
      state_f <= stg9;
      ctrl <= doLoad3;
```

```
        end

        stg9 :
        begin
          state_f <= stg10;
          ctrl <= doGenMult; //  betaV * cosElecTheta
        end

        stg10 :
        begin
          state_f <= stg11;
          ctrl <= doSave3;
        end

        stg11 :
        begin
          state_f <= stg12;
          ctrl <= doLoad4;
        end

        stg12 :
        begin
          state_f <= stg13;
          ctrl <= doGenMult; // alphaV * sinElecTheta
        end

        stg13 :
        begin
          state_f <= stg14;
          ctrl <= doSave4;
        end

        stg14 :
        begin
          state_f <= stg15;
          ctrl <= doLoad5;
        end

        stg15 :
        begin
          state_f <= stg16;
          ctrl <= doGenAdd; //  betaV_cosElecTheta +
    alphaV_sinElecTheta
        end

        stg16 :
        begin
          state_f <= idle;
          ctrl <= doSave5;
        end

        default :
          begin
            ctrl <= doNothing;
            state_f <= idle;
          end
      endcase
```

```
    end

  always @(posedge clk)
    begin
      if (rst)
        state_p <= idle;
      else
        state_p <= state_f;
    end

//  always @(ctrl or str_load)
  always @(posedge clk)
    begin

      if (str_load)
      begin
        case(id)
          id_0 :
          begin
            inMult_a <= alphaV;
            inMult_b <= cosElecTheta;
          end

          id_1 :
          begin
            inMult_a <= betaV;
            inMult_b <= sinElecTheta;
          end

          id_2 :
          begin
            inAdd_a <= alphaV_cosElecTheta;
            inAdd_b <=  betaV_sinElecTheta;
          end

          id_3 :
          begin
            inMult_a <= betaV;
            inMult_b <= cosElecTheta;
          end

          id_4 :
          begin
            inMult_a <= alphaV;
            inMult_b <= sinElecTheta;
          end

          id_5 :
          begin
            inAdd_a <=  betaV_cosElecTheta;
            inAdd_b <=-alphaV_sinElecTheta;
          end

          default :
            begin
              inMult_a <= 0;
              inMult_b <= 0;
```

```
          inAdd_a <= 0;
          inAdd_b <= 0;
        end
    endcase
  end

 end

// alphaV_cosElecTheta activation
always@(posedge clk)
 begin
  if(rst)
    alphaV_cosElecTheta <= 0;
  else
    begin
      if (en)
        begin
          if (str_save0)
            alphaV_cosElecTheta <= res_mult;
        end
      else
        alphaV_cosElecTheta <= 0;
    end

 end

// betaV_sinElecTheta activation
always@(posedge clk)
 begin
  if(rst)
    betaV_sinElecTheta <= 0;
  else
    begin
      if (en)
        begin
          if (str_save1)
            betaV_sinElecTheta <= res_mult;
        end
      else
        betaV_sinElecTheta <= 0;
    end

 end

// dV activation
always@(posedge clk)
 begin
  if(rst)
    dV <= 0;
  else
    begin
      if (en)
        begin
          if (str_save2)
            dV <= res_add;
        end
      else
```

```
          dV <= 0;
        end

    end

  // betaV_cosElecTheta activation
  always@(posedge clk)
    begin
      if(rst)
        betaV_cosElecTheta <= 0;
      else
        begin
          if (en)
            begin
              if (str_save3)
                betaV_cosElecTheta <= res_mult;
            end
          else
            betaV_cosElecTheta <= 0;
        end

    end

  // alphaV_sinElecTheta activation
  always@(posedge clk)
    begin
      if(rst)
        alphaV_sinElecTheta <= 0;
      else
        begin
          if (en)
            begin
              if (str_save4)
                alphaV_sinElecTheta <= res_mult;
            end
          else
            alphaV_sinElecTheta <= 0;
        end

    end

  // qV activation
  always@(posedge clk)
    begin
      if(rst)
        qV <= 0;
      else
        begin
          if (en)
            begin
              if (str_save5)
                qV <= res_add;
            end
          else
            qV <= 0;
        end

```

```
    end

    fix_sig_mult_nbit #(.n(n), .m(m), .k(k))
  dut_mult
  (
    .clk(clk),
    .rst(rst),
    .en(en),
    .str(str_mult),
    .mult_a(inMult_a),
    .mult_b(inMult_b),
    .mult_out(res_mult)
  );

  addsub #( .n(n))
  dut_add
  (
    .clk(clk),
    .rst(rst),
    .en(en),
    .str(str_add),
    .iVal_a(inAdd_a),
    .iVal_b(inAdd_b),
    .oVal_z(res_add)
  );


endmodule
```

11 dFlux_seq.v

This is a demo of the module dFlux_seq.v and it is part of the Power Electronics Emulator project. This code is shared as a work-in-progress version due to the internal politics of our household Institute. The released version will be available upon request.

```
`timescale 1ns/100ps
// +FHDR ------------------------------------------------------------------------
// Copyright (c) 2020 INAOE All rights reserved
// INSTITUTO NACIONAL DE ASTROF?SICA, OPTICA Y ELECTRONICA
// COORDINACI?N DE ELECTR?NICA
// GRUPO DE INSTRUMENTACI?N
// Confidential Proprietary
// -----------------------------------------------------------------------------
// FILE NAME  :   svm_gen.v
// DEPARTMENT :   GRUPO DE SISTEMAS DIGITALES
// AUTHOR     :   Julio Hernandez
// AUTHOR?S EMAIL:  julio.hernandez@inaoep.mx
// -----------------------------------------------------------------------------
// RELEASE HISTORY
// VERSION DATE AUTHOR DESCRIPTION
//
// 1V0 200509 First test.
// -----------------------------------------------------------------------------
// PURPOSE  : This module implements a space vector modulation generator.
// -FHDR ------------------------------------------------------------------------


module dFlux_seq
#(  parameter   n = 32, m= 64, k=24, j=3, Rs= 12582912, Ts= 16, j_shift_bit= 10 , j_factor= 13328335 )
(
  input clk, rst, en, str,
  output busy,

  input signed [n-1:0] dqV, dqI, dqFlux, elecOmega,

  output signed [n-1:0] dFlux
);
// -----------------------------------------------------------------------------
// Top module local parameters definition
  localparam[4:0]
  idle  = 5'b10000,   // Idle
```

```
  stg0  = 5'b00000,   //
  stg1  = 5'b00001,   //
  stg2  = 5'b00010,   //
  stg3  = 5'b00011,   //
  stg4  = 5'b00100,   //
  stg5  = 5'b00101,   //
  stg6  = 5'b00110,   //
  stg7  = 5'b00111,   //
  stg8  = 5'b01000,   //
  stg9  = 5'b01001,   //
  stg10   = 5'b01010,   //
  stg11   = 5'b01011,   //
  stg12   = 5'b01100,   //
  stg13 = 5'b01101,   //
  stg14   = 5'b01110,   //
  stg15   = 5'b01111;   //

  localparam [7:0]    // Control local parameters
  doNothing = 8'b00000000,  //
  doLoad0   = 8'b00001000,  //
  doLoad1   = 8'b00001001,  //
  doLoad2   = 8'b00001010,  //
  doLoad3   = 8'b00001011,  //
  doLoad4   = 8'b00001100,  //
  doLoad5   = 8'b00001101,  //

  doSave0   = 8'b00010000,  //
  doSave1   = 8'b00010001,  //
  doSave2   = 8'b00010010,  //
  doSave3   = 8'b00010011,  //
  doSave4   = 8'b00010100,  //
  doSave5   = 8'b00010101,  //

  doGenMult = 8'b00100000,  //
  doGenAdd  = 8'b01000000,  //
  doIntegrator= 8'b10000000;  //

  localparam [2:0]    // id values
  id_0  = 3'd0, //
  id_1  = 3'd1, //
  id_2  = 3'd2, //
  id_3  = 3'd3, //
  id_4  = 3'd4; //


//
    ----------------------------------------------------------------------------

// Top module local reg definition
  // ctrl : Run the Counter process
  reg [7:0] ctrl;

  reg [4:0] state_p, state_f;

//
    ----------------------------------------------------------------------------
```

```
// Wire & Reg local definition

  wire      [j-1:0] id;

  reg signed    [n-1:0] inMult_a;
  reg signed    [n-1:0] inMult_b;
  reg signed    [n-1:0] inAdd_a;
  reg signed    [n-1:0] inAdd_b;

  wire signed   [n-1:0] res_mult;
  wire signed   [n-1:0] res_add;

  reg signed    [n-1:0] jFactor;

  reg signed    [n-1:0] dqFlux_shift,  dqFlux_shift_elecOmega, dqFlux_shift_elecOmega_jFactor;
  reg signed    [n-1:0] Rs_dqI;
  reg signed    [n-1:0] dqV_Rs_dqI, dqV_Rs_dqI_dqFlux_shift_elecOmega_jFactor;




  wire str_load, str_mult, str_add;
  wire str_load0;
  wire str_save0, str_save1, str_save2, str_save3, str_save4;

  wire str_integrator, busy_integrator;

// -----------------------------------------------------------------------------

// Top module assignations definition
  assign busy = state_p[4];

  assign id           = {ctrl[2],ctrl[1],ctrl[0]};

  assign str_load   = ctrl[3];
  assign str_mult   = ctrl[5];
  assign str_add    = ctrl[6];
  assign str_integrator = ctrl[7];

  assign str_load0  = (ctrl[3] & ~ctrl[2] & ~ctrl[1] & ~ctrl[0]) ? 1'b1 : 1'b0;

  assign str_save0  = (ctrl[4] & ~ctrl[2] & ~ctrl[1] & ~ctrl[0]) ? 1'b1 : 1'b0;
  assign str_save1  = (ctrl[4] & ~ctrl[2] & ~ctrl[1] &  ctrl[0]) ? 1'b1 : 1'b0;
  assign str_save2  = (ctrl[4] & ~ctrl[2] &  ctrl[1] & ~ctrl[0]) ? 1'b1 : 1'b0;
  assign str_save3  = (ctrl[4] & ~ctrl[2] &  ctrl[1] &  ctrl[0]) ? 1'b1 : 1'b0;
  assign str_save4  = (ctrl[4] &  ctrl[2] & ~ctrl[1] & ~ctrl[0]) ? 1'b1 : 1'b0;

  // state machine activation
```

```
  always @(state_p or str or busy_integrator)
    begin
      state_f <= state_p;
      ctrl <= doNothing;

      case(state_p)
        idle :
        begin
          if (str)
            begin
              state_f <=stg0;
              ctrl <= doLoad0;
            end
        end

        stg0 :
        begin
          state_f <= stg1;
          ctrl <= doGenMult; // dqFlux_shift * elecOmega
        end

        stg1 :
        begin
          state_f <= stg2;
          ctrl <= doSave0;
        end

        stg2 :
        begin
          state_f <= stg3;
          ctrl <= doLoad1;
        end

        stg3 :
        begin
          state_f <= stg4;
          ctrl <= doGenMult; //  dqFlux_shift_elecOmega * jFactor
        end

        stg4 :
        begin
          state_f <= stg5;
          ctrl <= doSave1;
        end

        stg5 :
        begin
          state_f <= stg6;
          ctrl <= doLoad2;
        end

        stg6 :
        begin
          state_f <= stg7;
          ctrl <= doGenMult; // Rs * dqI
        end

```

```
        stg7 :
        begin
          state_f <= stg8;
          ctrl <= doSave2;
        end

        stg8 :
        begin
          state_f <= stg9;
          ctrl <= doLoad3;
        end

        stg9 :
        begin
          state_f <= stg10;
          ctrl <= doGenAdd; //  dqV - Rs_dqI
        end

        stg10 :
        begin
          state_f <= stg11;
          ctrl <= doSave3;
        end

        stg11 :
        begin
          state_f <= stg12;
          ctrl <= doLoad4;
        end

        stg12 :
        begin
          state_f <= stg13;
          ctrl <= doGenAdd; // dqV_Rs_dqI +
    dqFlux_shift_elecOmega_jFactor
        end

        stg13 :
        begin
          state_f <= stg14;
          ctrl <= doSave4;
        end

        stg14 :
        begin
          state_f <= stg15;
          ctrl <= doIntegrator;
        end

        stg15 :
        begin
          if (busy_integrator)
            begin
              state_f <= idle;
              ctrl <= doNothing; //  dqFlux =
    dqV_Rs_dqI_dqFlux_shift_elecOmega_jFactor
            end
```

```
        end

        default :
          begin
            ctrl <= doNothing;
            state_f <= idle;
          end
      endcase
    end

  always @(posedge clk)
    begin
      if (rst)
        state_p <= idle;
      else
        state_p <= state_f;
    end

//  always @(ctrl or str_load)
  always @(posedge clk)
    begin

      if (str_load)
      begin
        case(id)
          id_0 :
          begin
            inMult_a <= dqFlux_shift;
            inMult_b <= elecOmega;
          end

          id_1 :
          begin
            inMult_a <= dqFlux_shift_elecOmega;
            inMult_b <= jFactor;
          end

          id_2 :
          begin
            inMult_a <= Rs;
            inMult_b <= dqI;
          end

          id_3 :
          begin
            inAdd_a <= dqV;
            inAdd_b <=-Rs_dqI;
          end

          id_4 :
          begin
            inAdd_a <= dqV_Rs_dqI;
            inAdd_b <= dqFlux_shift_elecOmega_jFactor;
          end

          default :
            begin
```

```
              inMult_a <= 0;
              inMult_b <= 0;
              inAdd_a <= 0;
              inAdd_b <= 0;
            end
        endcase
      end

    end


    // jFactor activation
  always@(posedge clk)
    begin
      if(rst)
        jFactor <= 0;
      else
        begin
          if (en)
            begin
              if (str_load0)
                jFactor <= j_factor;
            end
          else
            jFactor <= 0;
        end

    end

  // dqFlux_shift activation
  always@(posedge clk)
    begin
      if(rst)
        dqFlux_shift <= 0;
      else
        begin
          if (en)
            begin
              if (str_load0)
                dqFlux_shift <= dqFlux <<< j_shift_bit;
            end
          else
            dqFlux_shift <= 0;
        end

    end

  // dqFlux_shift_elecOmega activation
  always@(posedge clk)
    begin
      if(rst)
        dqFlux_shift_elecOmega <= 0;
      else
        begin
          if (en)
            begin
              if (str_save0)
```

```
                dqFlux_shift_elecOmega <= res_mult;
            end
          else
            dqFlux_shift_elecOmega <= 0;
        end

    end

  // dqFlux_shift_elecOmega_jFactor activation
  always@(posedge clk)
    begin
      if(rst)
        dqFlux_shift_elecOmega_jFactor <= 0;
      else
        begin
          if (en)
            begin
              if (str_save1)
                dqFlux_shift_elecOmega_jFactor <= res_mult;
            end
          else
            dqFlux_shift_elecOmega_jFactor <= 0;
        end

    end

  // Rs_dqI activation
  always@(posedge clk)
    begin
      if(rst)
        Rs_dqI <= 0;
      else
        begin
          if (en)
            begin
              if (str_save2)
                Rs_dqI <= res_mult;
            end
          else
            Rs_dqI <= 0;
        end

    end

  // dqV_Rs_dqI activation
       always@(posedge clk)
           begin
               if(rst)
                   dqV_Rs_dqI <= 0;
               else
                   begin
                       if (en)
                           begin
                               if (str_save3)
                                   dqV_Rs_dqI <= res_add;
                           end
                       else
```

```
                              dqV_Rs_dqI <= 0;
                      end

              end

  // dqV_Rs_dqI_dqFlux_shift_elecOmega_jFactor activation
  always@(posedge clk)
    begin
      if(rst)
        dqV_Rs_dqI_dqFlux_shift_elecOmega_jFactor <= 0;
      else
        begin
          if (en)
            begin
              if (str_save4)
                dqV_Rs_dqI_dqFlux_shift_elecOmega_jFactor <=
    res_add;
            end
          else
            dqV_Rs_dqI_dqFlux_shift_elecOmega_jFactor <= 0;
        end

    end

    fix_sig_mult_nbit #(.n(n), .m(m), .k(k))
  dut_mult
  (
    .clk(clk),
    .rst(rst),
    .en(en),
    .str(str_mult),
    .mult_a(inMult_a),
    .mult_b(inMult_b),
    .mult_out(res_mult)
  );

  addsub #( .n(n))
  dut_add
  (
    .clk(clk),
    .rst(rst),
    .en(en),
    .str(str_add),
    .iVal_a(inAdd_a),
    .iVal_b(inAdd_b),
    .oVal_z(res_add)
  );

  integrator #(.n(n), .m(m), .k(k), .Ts(Ts))
  dut4(
    .clk(clk),
    .rst(rst),
    .str(str_integrator),
    .en(en),
    .busy(busy_integrator),
    .u(dqV_Rs_dqI_dqFlux_shift_elecOmega_jFactor),
    .y(dFlux)
```

```
  );


endmodule
```

12 qFlux_seq.v

This is a demo of the module qFlux_seq.v and it is part of the Power Electronics Emulator project. This code is shared as a work-in-progress version due to the internal politics of our household Institute. The released version will be available upon request.

```
`timescale 1ns/100ps
// +FHDR ------------------------------------------------------------------------
// Copyright (c) 2020 INAOE All rights reserved
// INSTITUTO NACIONAL DE ASTROF?SICA, OPTICA Y ELECTRONICA
// COORDINACI?N DE ELECTR?NICA
// GRUPO DE INSTRUMENTACI?N
// Confidential Proprietary
// -----------------------------------------------------------------------------
// FILE NAME  :   svm_gen.v
// DEPARTMENT :   GRUPO DE SISTEMAS DIGITALES
// AUTHOR    :   Julio Hernandez
// AUTHOR?S EMAIL:  julio.hernandez@inaoep.mx
// -----------------------------------------------------------------------------
// RELEASE HISTORY
// VERSION DATE AUTHOR DESCRIPTION
//
// 1V0 200509 First test.
// -----------------------------------------------------------------------------
// PURPOSE  : This module implements a space vector modulation generator.
// -FHDR ------------------------------------------------------------------------



module qFlux_seq
#(  parameter   n = 32, m= 64, k=24, j=3, Rs= 12582912, Ts= 16, j_shift_bit= 10 , j_factor= 13328335 )
(
  input clk, rst, en, str,
  output busy,

  input signed [n-1:0] dqV, dqI, dqFlux, elecOmega,

  output signed [n-1:0] qFlux
);
// -----------------------------------------------------------------------------
// Top module local parameters definition
  localparam[4:0]
  idle  = 5'b10000,   // Idle
```

```
  stg0  = 5'b00000,   //
  stg1  = 5'b00001,   //
  stg2  = 5'b00010,   //
  stg3  = 5'b00011,   //
  stg4  = 5'b00100,   //
  stg5  = 5'b00101,   //
  stg6  = 5'b00110,   //
  stg7  = 5'b00111,   //
  stg8  = 5'b01000,   //
  stg9  = 5'b01001,   //
  stg10   = 5'b01010,   //
  stg11   = 5'b01011,   //
  stg12   = 5'b01100,   //
  stg13 = 5'b01101,   //
  stg14   = 5'b01110,   //
  stg15   = 5'b01111;   //

  localparam [7:0]    // Control local parameters
  doNothing = 8'b00000000,  //
  doLoad0   = 8'b00001000,  //
  doLoad1   = 8'b00001001,  //
  doLoad2   = 8'b00001010,  //
  doLoad3   = 8'b00001011,  //
  doLoad4   = 8'b00001100,  //
  doLoad5   = 8'b00001101,  //

  doSave0   = 8'b00010000,  //
  doSave1   = 8'b00010001,  //
  doSave2   = 8'b00010010,  //
  doSave3   = 8'b00010011,  //
  doSave4   = 8'b00010100,  //
  doSave5   = 8'b00010101,  //

  doGenMult = 8'b00100000,  //
  doGenAdd  = 8'b01000000,  //
  doIntegrator= 8'b10000000;  //

  localparam [2:0]    // id values
  id_0  = 3'd0, //
  id_1  = 3'd1, //
  id_2  = 3'd2, //
  id_3  = 3'd3, //
  id_4  = 3'd4; //


//
    -----------------------------------------------------------------------------
// Top module local reg definition
  // ctrl : Run the Counter process
  reg [7:0] ctrl;

  reg [4:0] state_p, state_f;

//
    -----------------------------------------------------------------------------
```

```verilog
// Wire & Reg local definition

  wire      [j-1:0] id;

  reg signed    [n-1:0] inMult_a;
  reg signed    [n-1:0] inMult_b;
  reg signed    [n-1:0] inAdd_a;
  reg signed    [n-1:0] inAdd_b;

  wire signed   [n-1:0] res_mult;
  wire signed   [n-1:0] res_add;

  reg signed    [n-1:0] jFactor;

  reg signed    [n-1:0] dqFlux_shift,  dqFlux_shift_elecOmega, dqFlux_shift_elecOmega_jFactor;
  reg signed    [n-1:0] Rs_dqI;
  reg signed    [n-1:0] dqV_Rs_dqI, dqV_Rs_dqI_dqFlux_shift_elecOmega_jFactor;




  wire str_load, str_mult, str_add;
  wire str_load0;
  wire str_save0, str_save1, str_save2, str_save3, str_save4;

  wire str_integrator, busy_integrator;

// -----------------------------------------------------------------------------
// Top module assignations definition
  assign busy = state_p[4];

  assign id           = {ctrl[2],ctrl[1],ctrl[0]};

  assign str_load   = ctrl[3];
  assign str_mult   = ctrl[5];
  assign str_add    = ctrl[6];
  assign str_integrator = ctrl[7];

  assign str_load0  = (ctrl[3] & ~ctrl[2] & ~ctrl[1] & ~ctrl[0]) ? 1'b1 : 1'b0;

  assign str_save0  = (ctrl[4] & ~ctrl[2] & ~ctrl[1] & ~ctrl[0]) ? 1'b1 : 1'b0;
  assign str_save1  = (ctrl[4] & ~ctrl[2] & ~ctrl[1] &  ctrl[0]) ? 1'b1 : 1'b0;
  assign str_save2  = (ctrl[4] & ~ctrl[2] &  ctrl[1] & ~ctrl[0]) ? 1'b1 : 1'b0;
  assign str_save3  = (ctrl[4] & ~ctrl[2] &  ctrl[1] &  ctrl[0]) ? 1'b1 : 1'b0;
  assign str_save4  = (ctrl[4] &  ctrl[2] & ~ctrl[1] & ~ctrl[0]) ? 1'b1 : 1'b0;

  // state machine activation
```

```
  always @(state_p or str or busy_integrator)
    begin
      state_f <= state_p;
      ctrl <= doNothing;

      case(state_p)
        idle :
        begin
          if (str)
            begin
              state_f <=stg0;
              ctrl <= doLoad0;
            end
        end

        stg0 :
        begin
          state_f <= stg1;
          ctrl <= doGenMult; // dqFlux_shift * elecOmega
        end

        stg1 :
        begin
          state_f <= stg2;
          ctrl <= doSave0;
        end

        stg2 :
        begin
          state_f <= stg3;
          ctrl <= doLoad1;
        end

        stg3 :
        begin
          state_f <= stg4;
          ctrl <= doGenMult; //  dqFlux_shift_elecOmega * jFactor
        end

        stg4 :
        begin
          state_f <= stg5;
          ctrl <= doSave1;
        end

        stg5 :
        begin
          state_f <= stg6;
          ctrl <= doLoad2;
        end

        stg6 :
        begin
          state_f <= stg7;
          ctrl <= doGenMult; // Rs * dqI
        end

```

```
        stg7 :
        begin
          state_f <= stg8;
          ctrl <= doSave2;
        end

        stg8 :
        begin
          state_f <= stg9;
          ctrl <= doLoad3;
        end

        stg9 :
        begin
          state_f <= stg10;
          ctrl <= doGenAdd; //  dqV - Rs_dqI
        end

        stg10 :
        begin
          state_f <= stg11;
          ctrl <= doSave3;
        end

        stg11 :
        begin
          state_f <= stg12;
          ctrl <= doLoad4;
        end

        stg12 :
        begin
          state_f <= stg13;
          ctrl <= doGenAdd; // dqV_Rs_dqI + dqFlux_shift_elecOmega_jFactor
        end

        stg13 :
        begin
          state_f <= stg14;
          ctrl <= doSave4;
        end

        stg14 :
        begin
          state_f <= stg15;
          ctrl <= doIntegrator;
        end

        stg15 :
        begin
          if (busy_integrator)
            begin
              state_f <= idle;
              ctrl <= doNothing; //  dqFlux = dqV_Rs_dqI_dqFlux_shift_elecOmega_jFactor
            end
```

```
        end

        default :
          begin
            ctrl <= doNothing;
            state_f <= idle;
          end
      endcase
    end

  always @(posedge clk)
    begin
      if (rst)
        state_p <= idle;
      else
        state_p <= state_f;
    end

//  always @(ctrl or str_load)
  always @(posedge clk)
    begin

      if (str_load)
      begin
        case(id)
          id_0 :
          begin
            inMult_a <= dqFlux_shift;
            inMult_b <= elecOmega;
          end

          id_1 :
          begin
            inMult_a <= dqFlux_shift_elecOmega;
            inMult_b <= jFactor;
          end

          id_2 :
          begin
            inMult_a <= Rs;
            inMult_b <= dqI;
          end

          id_3 :
          begin
            inAdd_a <= dqV;
            inAdd_b <=-Rs_dqI;
          end

          id_4 :
          begin
            inAdd_a <= dqV_Rs_dqI;
            inAdd_b <=-dqFlux_shift_elecOmega_jFactor;
          end

          default :
            begin
```

```
              inMult_a <= 0;
              inMult_b <= 0;
              inAdd_a <= 0;
              inAdd_b <= 0;
            end
        endcase
      end

    end


    // jFactor activation
  always@(posedge clk)
    begin
      if(rst)
        jFactor <= 0;
      else
        begin
          if (en)
            begin
              if (str_load0)
                jFactor <= j_factor;
            end
          else
            jFactor <= 0;
        end

    end

  // dqFlux_shift activation
  always@(posedge clk)
    begin
      if(rst)
        dqFlux_shift <= 0;
      else
        begin
          if (en)
            begin
              if (str_load0)
                dqFlux_shift <= dqFlux <<< j_shift_bit;
            end
          else
            dqFlux_shift <= 0;
        end

    end

  // dqFlux_shift_elecOmega activation
  always@(posedge clk)
    begin
      if(rst)
        dqFlux_shift_elecOmega <= 0;
      else
        begin
          if (en)
            begin
              if (str_save0)
```

```
                dqFlux_shift_elecOmega <= res_mult;
            end
          else
            dqFlux_shift_elecOmega <= 0;
        end

    end

  // dqFlux_shift_elecOmega_jFactor activation
  always@(posedge clk)
    begin
      if(rst)
        dqFlux_shift_elecOmega_jFactor <= 0;
      else
        begin
          if (en)
            begin
              if (str_save1)
                dqFlux_shift_elecOmega_jFactor <= res_mult;
            end
          else
            dqFlux_shift_elecOmega_jFactor <= 0;
        end

    end

  // Rs_dqI activation
  always@(posedge clk)
    begin
      if(rst)
        Rs_dqI <= 0;
      else
        begin
          if (en)
            begin
              if (str_save2)
                Rs_dqI <= res_mult;
            end
          else
            Rs_dqI <= 0;
        end

    end

  // dqV_Rs_dqI activation
          always@(posedge clk)
              begin
                  if(rst)
                      dqV_Rs_dqI <= 0;
                  else
                      begin
                          if (en)
                              begin
                                  if (str_save3)
                                      dqV_Rs_dqI <= res_add;
                              end
                          else
```

```
                                dqV_Rs_dqI <= 0;
                        end

                end

// dqV_Rs_dqI_dqFlux_shift_elecOmega_jFactor activation
always@(posedge clk)
  begin
    if(rst)
      dqV_Rs_dqI_dqFlux_shift_elecOmega_jFactor <= 0;
    else
      begin
        if (en)
          begin
            if (str_save4)
              dqV_Rs_dqI_dqFlux_shift_elecOmega_jFactor <=
  res_add;
          end
        else
          dqV_Rs_dqI_dqFlux_shift_elecOmega_jFactor <= 0;
      end

  end

  fix_sig_mult_nbit #(.n(n), .m(m), .k(k))
dut_mult
(
  .clk(clk),
  .rst(rst),
  .en(en),
  .str(str_mult),
  .mult_a(inMult_a),
  .mult_b(inMult_b),
  .mult_out(res_mult)
);

addsub #( .n(n))
dut_add
(
  .clk(clk),
  .rst(rst),
  .en(en),
  .str(str_add),
  .iVal_a(inAdd_a),
  .iVal_b(inAdd_b),
  .oVal_z(res_add)
);

integrator #(.n(n), .m(m), .k(k), .Ts(Ts))
dut4(
  .clk(clk),
  .rst(rst),
  .str(str_integrator),
  .en(en),
  .busy(busy_integrator),
  .u(dqV_Rs_dqI_dqFlux_shift_elecOmega_jFactor),
  .y(qFlux)
```

```
  );


endmodule
```

13 dqI_elecTorque_seq.v

This is a demo of the module dqI_elecTorque_seq.v and it is part of the Power Electronics Emulator project. This code is shared as a work-in-progress version due to the internal politics of our household Institute. The released version will be available upon request.

```
`timescale 1ns/100ps
// +FHDR------------------------------------------------------------------------
// Copyright (c) 2020 INAOE All rights reserved
// INSTITUTO NACIONAL DE ASTROF?SICA, OPTICA Y ELECTRONICA
// COORDINACI?N DE ELECTR?NICA
// GRUPO DE INSTRUMENTACI?N
// Confidential Proprietary
// -----------------------------------------------------------------------------
// FILE NAME  :   svm_gen.v
// DEPARTMENT :   GRUPO DE SISTEMAS DIGITALES
// AUTHOR   :   Julio Hernandez
// AUTHOR?S EMAIL:  julio.hernandez@inaoep.mx
// -----------------------------------------------------------------------------
// RELEASE HISTORY
// VERSION DATE AUTHOR DESCRIPTION
//
// 1V0 200509 First test.
// -----------------------------------------------------------------------------
// PURPOSE  : This module implements a space vector modulation generator.
// -FHDR------------------------------------------------------------------------



module dqI_elecTorque_seq
#(  parameter   n = 32, m= 64, k=24, j=3, pmsm_pm= 85832, dqL_shift_bit= 10, qdL_factor= 15603809, pmsm_pp_three_over_two = 100663296)
(
  input clk, rst, en, str,
  output busy,

  input signed [n-1:0] dFlux, qFlux,

  output signed [n-1:0] dI, qI,
  output reg signed [n-1:0] elecTorque
);
// -----------------------------------------------------------------------------
// Top module local parameters definition
```

```
localparam[5:0]
idle  = 6'b100000,    // Idle
stg0  = 6'b000000,    //
stg1  = 6'b000001,    //
stg2  = 6'b000010,    //
stg3  = 6'b000011,    //
stg4  = 6'b000100,    //
stg5  = 6'b000101,    //
stg6  = 6'b000110,    //
stg7  = 6'b000111,    //
stg8  = 6'b001000,    //
stg9  = 6'b001001,    //
stg10   = 6'b001010,    //
stg11   = 6'b001011,    //
stg12   = 6'b001100,    //
stg13 = 6'b001101,    //
stg14   = 6'b001110,    //
stg15   = 6'b001111,    //
stg16 = 6'b010000,    //
stg17 = 6'b010001,    //
stg18 = 6'b010010,    //
stg19 = 6'b010011,    //
stg20 = 6'b010100;    //

localparam [7:0]    // Control local parameters
doNothing = 8'b00000000,  //
doLoad0   = 8'b00001000,  //
doLoad1   = 8'b00001001,  //
doLoad2   = 8'b00001010,  //
doLoad3   = 8'b00001011,  //
doLoad4   = 8'b00001100,  //
doLoad5   = 8'b00001101,  //
doLoad6   = 8'b00001110,  //

doSave0   = 8'b00010000,  //
doSave1   = 8'b00010001,  //
doSave2   = 8'b00010010,  //
doSave3   = 8'b00010011,  //
doSave4   = 8'b00010100,  //
doSave5   = 8'b00010101,  //
doSave6   = 8'b00010110,  //

doGenMult = 8'b00100000,  //
doGenAdd  = 8'b01000000,  //
doShift   = 8'b10000000;  //

localparam [2:0]    // id values
id_0  = 3'd0, //
id_1  = 3'd1, //
id_2  = 3'd2, //
id_3  = 3'd3, //
id_4  = 3'd4, //
id_5  = 3'd5, //
id_6  = 3'd6; //
```

```
//  -----------------------------------------------------------------------------
// Top module local reg definition
  // ctrl : Run the Counter process
  reg [7:0] ctrl;

  reg [5:0] state_p, state_f;

//  -----------------------------------------------------------------------------
// Wire & Reg local definition

  wire       [j-1:0] id;

  reg signed    [n-1:0] inMult_a;
  reg signed    [n-1:0] inMult_b;
  reg signed    [n-1:0] inAdd_a;
  reg signed    [n-1:0] inAdd_b;

  wire signed   [n-1:0] res_mult;
  wire signed   [n-1:0] res_add;

  reg signed    [n-1:0] qdLFactor, pmsmFactor;

  reg signed    [n-1:0] dFlux_shift,  qFlux_shift;
  reg signed    [n-1:0] dFlux_pmsm_pm, dI_aux, qI_aux;
  reg signed    [n-1:0] dFlux_qI_aux, qFlux_dI_aux,
    dFlux_qI_aux_qFlux_dI_aux;

  wire str_load, str_mult, str_add;
  wire str_save0, str_save1, str_save2, str_save3, str_save4,
    str_save5, str_save6;

  wire str_shift;

//  -----------------------------------------------------------------------------
// Top module assignations definition
  assign busy = state_p[5];

  assign id           = {ctrl[2],ctrl[1],ctrl[0]};

  assign str_load   = ctrl[3];
  assign str_mult   = ctrl[5];
  assign str_add    = ctrl[6];
  assign str_shift  = ctrl[7];

  assign str_save0  = (ctrl[4] & ~ctrl[2] & ~ctrl[1] & ~ctrl[0]) ?
    1'b1 : 1'b0;
  assign str_save1  = (ctrl[4] & ~ctrl[2] & ~ctrl[1] &  ctrl[0]) ?
    1'b1 : 1'b0;
  assign str_save2  = (ctrl[4] & ~ctrl[2] &  ctrl[1] & ~ctrl[0]) ?
    1'b1 : 1'b0;
```

```verilog
assign str_save3  = (ctrl[4] & ~ctrl[2] &  ctrl[1] &  ctrl[0]) ? 1'b1 : 1'b0;
assign str_save4  = (ctrl[4] &  ctrl[2] & ~ctrl[1] & ~ctrl[0]) ? 1'b1 : 1'b0;
assign str_save5  = (ctrl[4] &  ctrl[2] & ~ctrl[1] &  ctrl[0]) ? 1'b1 : 1'b0;
assign str_save6  = (ctrl[4] &  ctrl[2] &  ctrl[1] & ~ctrl[0]) ? 1'b1 : 1'b0;

assign dI= dI_aux;
assign qI= qI_aux;

// state machine activation
always @(state_p or str)
  begin
    state_f <= state_p;
    ctrl <= doNothing;

    case(state_p)
      idle :
      begin
        if (str)
          begin
            state_f <=stg0;
            ctrl <= doLoad0;
          end
      end

      stg0 :
      begin
        state_f <= stg1;
        ctrl <= doGenAdd; // dFlux + pmsm_pm
      end

      stg1 :
      begin
        state_f <= stg2;
        ctrl <= doSave0;
      end

      stg2 :
      begin
        state_f <= stg3;
        ctrl <= doShift; // dFlux_shift, qFlux_shift, dqLFactor
      end

      stg3 :
      begin
        state_f <= stg4;
        ctrl <= doLoad1;
      end

      stg4 :
      begin
        state_f <= stg5;
        ctrl <= doGenMult; //  dFlux_shift * dqLFactor
      end
```

```
        stg5 :
        begin
          state_f <= stg6;
          ctrl <= doSave1;
        end

        stg6 :
        begin
          state_f <= stg7;
          ctrl <= doLoad2;
        end

        stg7 :
        begin
          state_f <= stg8;
          ctrl <= doGenMult; // qFlux_shift * dqLFactor
        end

        stg8 :
        begin
          state_f <= stg9;
          ctrl <= doSave2;
        end

        stg9 :
        begin
          state_f <= stg10;
          ctrl <= doLoad3;
        end

        stg10 :
        begin
          state_f <= stg11;
          ctrl <= doGenMult; // dFlux * qI_aux
        end

        stg11 :
        begin
          state_f <= stg12;
          ctrl <= doSave3;
        end

        stg12 :
        begin
          state_f <= stg13;
          ctrl <= doLoad4;
        end

        stg13 :
        begin
          state_f <= stg14;
          ctrl <= doGenMult; // qFlux * dI_aux
        end

        stg14 :
        begin
```

```
          state_f <= stg15;
          ctrl <= doSave4;
        end

        stg15 :
        begin
          state_f <= stg16;
          ctrl <= doLoad5;
        end

        stg16 :
        begin
          state_f <= stg17;
          ctrl <= doGenAdd; // dFlux_qI_aux - qFlux_dI_aux
        end

        stg17 :
        begin
          state_f <= stg18;
          ctrl <= doSave5;
        end

        stg18 :
        begin
          state_f <= stg19;
          ctrl <= doLoad6;
        end

        stg19 :
        begin
          state_f <= stg20;
          ctrl <= doGenMult; // dFlux_qI_aux_qFlux_dI_aux * pmsmFactor
        end

        stg20 :
        begin
          state_f <= idle;
          ctrl <= doSave6;
        end

        default :
          begin
            ctrl <= doNothing;
            state_f <= idle;
          end
      endcase
    end

  always @(posedge clk)
    begin
      if (rst)
        state_p <= idle;
      else
        state_p <= state_f;
    end

```

```
//  always @(ctrl or str_load)
  always @(posedge clk)
    begin

      if (str_load)
      begin
        case(id)
          id_0 :
          begin
            inAdd_a <= dFlux;
            inAdd_b <=-pmsm_pm;
          end

          id_1 :
          begin
            inMult_a <= dFlux_shift;
            inMult_b <= qdLFactor;
          end

          id_2 :
          begin
            inMult_a <= qFlux_shift;
            inMult_b <= qdLFactor;
          end

          id_3 :
          begin
            inMult_a <= dFlux;
            inMult_b <= qI_aux;
          end

          id_4 :
          begin
            inMult_a <= qFlux;
            inMult_b <= dI_aux;
          end

          id_5 :
          begin
            inAdd_a <= dFlux_qI_aux;
            inAdd_b <=-qFlux_dI_aux;
          end

          id_6 :
          begin
            inMult_a <= dFlux_qI_aux_qFlux_dI_aux;
            inMult_b <= pmsmFactor;
          end

          default :
            begin
              inMult_a <= 0;
              inMult_b <= 0;
              inAdd_a <= 0;
              inAdd_b <= 0;
            end
        endcase
```

```
      end

    end


  // dFlux_pmsm_pm activation
  always@(posedge clk)
    begin
      if(rst)
        dFlux_pmsm_pm <= 0;
      else
        begin
          if (en)
            begin
              if (str_save0)
                dFlux_pmsm_pm <= res_add;
            end
          else
            dFlux_pmsm_pm <= 0;
        end

    end

  // dFlux_shift activation
  always@(posedge clk)
    begin
      if(rst)
        dFlux_shift <= 0;
      else
        begin
          if (en)
            begin
              if (str_shift)
                dFlux_shift <= dFlux_pmsm_pm <<< dqL_shift_bit;
            end
          else
            dFlux_shift <= 0;
        end

    end

  // qFlux_shift activation
  always@(posedge clk)
    begin
      if(rst)
        qFlux_shift <= 0;
      else
        begin
          if (en)
            begin
              if (str_shift)
                qFlux_shift <= qFlux <<< dqL_shift_bit;
            end
          else
            qFlux_shift <= 0;
        end

```

```
    end

  // qdLFactor activation
  always@(posedge clk)
    begin
      if(rst)
        qdLFactor <= 0;
      else
        begin
          if (en)
            begin
              if (str_shift)
                qdLFactor <= qdL_factor;
            end
          else
            qdLFactor <= 0;
        end

    end

  // pmsmFactor activation
  always@(posedge clk)
    begin
      if(rst)
        pmsmFactor <= 0;
      else
        begin
          if (en)
            begin
              if (str_shift)
                pmsmFactor <= pmsm_pp_three_over_two;
            end
          else
            pmsmFactor <= 0;
        end

    end

  // dI_aux activation
  always@(posedge clk)
    begin
      if(rst)
        dI_aux <= 0;
      else
        begin
          if (en)
            begin
              if (str_save1)
                dI_aux <= res_mult;
            end
          else
            dI_aux <= 0;
        end

    end

  // qI_aux activation
```

```
always@(posedge clk)
  begin
    if(rst)
      qI_aux <= 0;
    else
      begin
        if (en)
          begin
            if (str_save2)
              qI_aux <= res_mult;
          end
        else
          qI_aux <= 0;
      end

  end

// dFlux_qI_aux activation
always@(posedge clk)
  begin
    if(rst)
      dFlux_qI_aux <= 0;
    else
      begin
        if (en)
          begin
            if (str_save3)
              dFlux_qI_aux <= res_mult;
          end
        else
          dFlux_qI_aux <= 0;
      end

  end

// qFlux_dI_aux activation
always@(posedge clk)
  begin
    if(rst)
      qFlux_dI_aux <= 0;
    else
      begin
        if (en)
          begin
            if (str_save4)
              qFlux_dI_aux <= res_mult;
          end
        else
          qFlux_dI_aux <= 0;
      end

  end

// dFlux_qI_aux_qFlux_dI_aux activation
always@(posedge clk)
  begin
    if(rst)
```

```
        dFlux_qI_aux_qFlux_dI_aux <= 0;
      else
        begin
          if (en)
            begin
              if (str_save5)
                dFlux_qI_aux_qFlux_dI_aux <= res_add;
            end
          else
            dFlux_qI_aux_qFlux_dI_aux <= 0;
        end

    end

  // elecTorque activation
  always@(posedge clk)
    begin
      if(rst)
        elecTorque <= 0;
      else
        begin
          if (en)
            begin
              if (str_save6)
                elecTorque <= res_mult;
            end
          else
            elecTorque <= 0;
        end

    end

    fix_sig_mult_nbit #(.n(n), .m(m), .k(k))
  dut_mult
  (
    .clk(clk),
    .rst(rst),
    .en(en),
    .str(str_mult),
    .mult_a(inMult_a),
    .mult_b(inMult_b),
    .mult_out(res_mult)
  );

  addsub #( .n(n))
  dut_add
  (
    .clk(clk),
    .rst(rst),
    .en(en),
    .str(str_add),
    .iVal_a(inAdd_a),
    .iVal_b(inAdd_b),
    .oVal_z(res_add)
  );


```

```
endmodule
```

14 dqI_ph3_seq.v

This is a demo of the module dqI_ph3_seq.v and it is part of the Power Electronics Emulator project. This code is shared as a work-in-progress version due to the internal politics of our household Institute. The released version will be available upon request.

```
`timescale 1ns/100ps
// +FHDR -------------------------------------------------------------------------
// Copyright (c) 2020 INAOE All rights reserved
// INSTITUTO NACIONAL DE ASTROF?SICA, OPTICA Y ELECTRONICA
// COORDINACI?N DE ELECTR?NICA
// GRUPO DE INSTRUMENTACI?N
// Confidential Proprietary
// ------------------------------------------------------------------------------
// FILE NAME  :   svm_gen.v
// DEPARTMENT :   GRUPO DE SISTEMAS DIGITALES
// AUTHOR     :   Julio Hernandez
// AUTHOR?S EMAIL:  julio.hernandez@inaoep.mx
// ------------------------------------------------------------------------------
// RELEASE HISTORY
// VERSION DATE AUTHOR DESCRIPTION
//
// 1V0 200509 First test.
// ------------------------------------------------------------------------------
// PURPOSE  : This module implements a space vector modulation generator.
// -FHDR -------------------------------------------------------------------------



module dqI_ph3_seq
#(  parameter   n = 32, m= 64, k=24, j=3, math_sqrt_three_over_two= 14529495 )
(
  input clk, rst, en, str,
  output busy,

  input signed [n-1:0] dI, qI, cosElecTheta, sinElecTheta,

  output signed [n-1:0] uI,
  output reg signed [n-1:0] vI
);
// ------------------------------------------------------------------------------
// Top module local parameters definition
  localparam[5:0]
```

```
idle  = 6'b100000,    // Idle
stg0  = 6'b000000,    //
stg1  = 6'b000001,    //
stg2  = 6'b000010,    //
stg3  = 6'b000011,    //
stg4  = 6'b000100,    //
stg5  = 6'b000101,    //
stg6  = 6'b000110,    //
stg7  = 6'b000111,    //
stg8  = 6'b001000,    //
stg9  = 6'b001001,    //
stg10   = 6'b001010,    //
stg11   = 6'b001011,    //
stg12   = 6'b001100,    //
stg13 = 6'b001101,    //
stg14   = 6'b001110,    //
stg15   = 6'b001111,    //
stg16 = 6'b010000,    //
stg17 = 6'b010001,    //
stg18 = 6'b010010,    //
stg19 = 6'b010011,    //
stg20 = 6'b010100,    //
stg21 = 6'b010101,    //
stg22 = 6'b010110;    //

localparam [6:0]    // Control local parameters
doNothing = 7'b0000000, //
doLoad0   = 7'b0001000, //
doLoad1   = 7'b0001001, //
doLoad2   = 7'b0001010, //
doLoad3   = 7'b0001011, //
doLoad4   = 7'b0001100, //
doLoad5   = 7'b0001101, //
doLoad6   = 7'b0001110, //
doLoad7   = 7'b0001111, //

doSave0   = 7'b0010000, //
doSave1   = 7'b0010001, //
doSave2   = 7'b0010010, //
doSave3   = 7'b0010011, //
doSave4   = 7'b0010100, //
doSave5   = 7'b0010101, //
doSave6   = 7'b0010110, //
doSave7   = 7'b0010111, //

doGenMult = 7'b0100000, //
doGenAdd  = 7'b1000000; //

localparam [2:0]    // id values
id_0  = 3'd0, //
id_1  = 3'd1, //
id_2  = 3'd2, //
id_3  = 3'd3, //
id_4  = 3'd4, //
id_5  = 3'd5, //
id_6  = 3'd6, //
id_7  = 3'd7; //
```

```verilog

// -----------------------------------------------------------------------------
// Top module local reg definition
  // ctrl : Run the Counter process
  reg [6:0] ctrl;

  reg [5:0] state_p, state_f;

// -----------------------------------------------------------------------------
// Wire & Reg local definition

  wire      [j-1:0] id;

  reg signed    [n-1:0] inMult_a;
  reg signed    [n-1:0] inMult_b;
  reg signed    [n-1:0] inAdd_a;
  reg signed    [n-1:0] inAdd_b;

  wire signed   [n-1:0] res_mult;
  wire signed   [n-1:0] res_add;

  reg signed    [n-1:0] dI_cosElecTheta, qI_sinElecTheta;
  reg signed    [n-1:0] qI_cosElecTheta, dI_sinElecTheta;
  reg signed    [n-1:0] alphaI, betaI, alphaI_over_two, betaI_math_sqrt_three_over_two;

  wire str_load, str_mult, str_add;
  wire str_save0, str_save1, str_save2, str_save3, str_save4, str_save5, str_save6, str_save7;

// -----------------------------------------------------------------------------
// Top module assignations definition
  assign busy = state_p[5];

  assign id          = {ctrl[2],ctrl[1],ctrl[0]};

  assign str_load   = ctrl[3];
  assign str_mult   = ctrl[5];
  assign str_add    = ctrl[6];

  assign str_save0  = (ctrl[4] & ~ctrl[2] & ~ctrl[1] & ~ctrl[0]) ? 1'b1 : 1'b0;
  assign str_save1  = (ctrl[4] & ~ctrl[2] & ~ctrl[1] &  ctrl[0]) ? 1'b1 : 1'b0;
  assign str_save2  = (ctrl[4] & ~ctrl[2] &  ctrl[1] & ~ctrl[0]) ? 1'b1 : 1'b0;
  assign str_save3  = (ctrl[4] & ~ctrl[2] &  ctrl[1] &  ctrl[0]) ? 1'b1 : 1'b0;
  assign str_save4  = (ctrl[4] &  ctrl[2] & ~ctrl[1] & ~ctrl[0]) ? 1'b1 : 1'b0;
```

```
assign str_save5  = (ctrl[4] &  ctrl[2] & ~ctrl[1] &  ctrl[0]) ? 1'b1 : 1'b0;
assign str_save6  = (ctrl[4] &  ctrl[2] &  ctrl[1] & ~ctrl[0]) ? 1'b1 : 1'b0;
assign str_save7  = (ctrl[4] &  ctrl[2] &  ctrl[1] &  ctrl[0]) ? 1'b1 : 1'b0;

assign uI = alphaI;

// state machine activation
always @(state_p or str)
  begin
    state_f <= state_p;
    ctrl <= doNothing;

    case(state_p)
      idle :
      begin
        if (str)
          begin
            state_f <=stg0;
            ctrl <= doLoad0;
          end
      end

      stg0 :
      begin
        state_f <= stg1;
        ctrl <= doGenMult; // dI * cosElecTheta
      end

      stg1 :
      begin
        state_f <= stg2;
        ctrl <= doSave0;
      end

      stg2 :
      begin
        state_f <= stg3;
        ctrl <= doLoad1;
      end

      stg3 :
      begin
        state_f <= stg4;
        ctrl <= doGenMult; // qI * sinElecTheta
      end

      stg4 :
      begin
        state_f <= stg5;
        ctrl <= doSave1;
      end

      stg5 :
      begin
```

```
          state_f  <= stg6;
          ctrl  <= doLoad2;
        end

        stg6 :
        begin
          state_f  <= stg7;
          ctrl  <= doGenAdd; //  dI_cosElecTheta - qI_sinElecTheta
        end

        stg7 :
        begin
          state_f  <= stg8;
          ctrl  <= doSave2;
        end

        stg8 :
        begin
          state_f  <= stg9;
          ctrl  <= doLoad3;
        end

        stg9 :
        begin
          state_f  <= stg10;
          ctrl  <= doGenMult; //  qI * cosElecTheta
        end

        stg10 :
        begin
          state_f  <= stg11;
          ctrl  <= doSave3;
        end

        stg11 :
        begin
          state_f  <= stg12;
          ctrl  <= doLoad4;
        end

        stg12 :
        begin
          state_f  <= stg13;
          ctrl  <= doGenMult; // dI * sinElecTheta
        end

        stg13 :
        begin
          state_f  <= stg14;
          ctrl  <= doSave4;
        end

        stg14 :
        begin
          state_f  <= stg15;
          ctrl  <= doLoad5;
        end
```

```

      stg15 :
      begin
        state_f <= stg16;
        ctrl <= doGenAdd; //  qI_cosElecTheta + dI_sinElecTheta
      end

      stg16 :
      begin
        state_f <= stg17;
        ctrl <= doSave5;
      end

      stg17 :
      begin
        state_f <= stg18;
        ctrl <= doLoad6;
      end

      stg18 :
      begin
        state_f <= stg19;
        ctrl <= doGenAdd; //  betaI * math_sqrt_three_over_two , alphaI_over_two
      end

      stg19 :
      begin
        state_f <= stg20;
        ctrl <= doSave6;
      end

      stg20 :
      begin
        state_f <= stg21;
        ctrl <= doLoad7;
      end

      stg21 :
      begin
        state_f <= stg22;
        ctrl <= doGenAdd; //  betaI_math_sqrt_three_over_two - alphaI_over_two
      end

      stg22 :
      begin
        state_f <= idle;
        ctrl <= doSave7;
      end

      default :
        begin
          ctrl <= doNothing;
          state_f <= idle;
        end
    endcase
```

```
    end

  always @(posedge clk)
    begin
      if (rst)
        state_p <= idle;
      else
        state_p <= state_f;
    end

//  always @(ctrl or str_load)
  always @(posedge clk)
    begin

      if (str_load)
      begin
        case(id)
          id_0 :
          begin
            inMult_a <= dI;
            inMult_b <= cosElecTheta;
          end

          id_1 :
          begin
            inMult_a <= qI;
            inMult_b <= sinElecTheta;
          end

          id_2 :
          begin
            inAdd_a <= dI_cosElecTheta;
            inAdd_b <=-qI_sinElecTheta;
          end

          id_3 :
          begin
            inMult_a <= qI;
            inMult_b <= cosElecTheta;
          end

          id_4 :
          begin
            inMult_a <= dI;
            inMult_b <= sinElecTheta;
          end

          id_5 :
          begin
            inAdd_a <= qI_cosElecTheta;
            inAdd_b <= dI_sinElecTheta;
          end

          id_6 :
          begin
            inAdd_a <= betaI;
            inAdd_b <= math_sqrt_three_over_two;
```

```
          end

          id_7 :
          begin
            inAdd_a <= betaI_math_sqrt_three_over_two;
            inAdd_b <=-alphaI_over_two;
          end

          default :
            begin
              inMult_a <= 0;
              inMult_b <= 0;
              inAdd_a <= 0;
              inAdd_b <= 0;
            end
        endcase
      end

    end

  // dI_cosElecTheta activation
  always@(posedge clk)
    begin
      if(rst)
        dI_cosElecTheta <= 0;
      else
        begin
          if (en)
            begin
              if (str_save0)
                dI_cosElecTheta <= res_mult;
            end
          else
            dI_cosElecTheta <= 0;
        end

    end

  // qI_sinElecTheta activation
  always@(posedge clk)
    begin
      if(rst)
        qI_sinElecTheta <= 0;
      else
        begin
          if (en)
            begin
              if (str_save1)
                qI_sinElecTheta <= res_mult;
            end
          else
            qI_sinElecTheta <= 0;
        end

    end

  // alphaI activation
```

```
always@(posedge clk)
  begin
    if(rst)
      alphaI <= 0;
    else
      begin
        if (en)
          begin
            if (str_save2)
              alphaI <= res_add;
          end
        else
          alphaI <= 0;
      end

  end

// qI_cosElecTheta activation
always@(posedge clk)
  begin
    if(rst)
      qI_cosElecTheta <= 0;
    else
      begin
        if (en)
          begin
            if (str_save3)
              qI_cosElecTheta <= res_mult;
          end
        else
          qI_cosElecTheta <= 0;
      end

  end

// dI_sinElecTheta activation
always@(posedge clk)
  begin
    if(rst)
      dI_sinElecTheta <= 0;
    else
      begin
        if (en)
          begin
            if (str_save4)
              dI_sinElecTheta <= res_mult;
          end
        else
          dI_sinElecTheta <= 0;
      end

  end

// betaI activation
always@(posedge clk)
  begin
    if(rst)
```

```
        betaI <= 0;
      else
        begin
          if (en)
            begin
              if (str_save5)
                betaI <= res_add;
            end
          else
            betaI <= 0;
        end

    end

  // betaI_math_sqrt_three_over_two activation
  always@(posedge clk)
    begin
      if(rst)
        betaI_math_sqrt_three_over_two <= 0;
      else
        begin
          if (en)
            begin
              if (str_save6)
                betaI_math_sqrt_three_over_two <= res_mult;
            end
          else
            betaI_math_sqrt_three_over_two <= 0;
        end

    end

  // alphaI_over_two activation
  always@(posedge clk)
    begin
      if(rst)
        alphaI_over_two <= 0;
      else
        begin
          if (en)
            begin
              if (str_save6)
                alphaI_over_two <= alphaI >>> 1;
            end
          else
            alphaI_over_two <= 0;
        end

    end

  // vI activation
  always@(posedge clk)
    begin
      if(rst)
        vI <= 0;
      else
        begin
```

```
          if (en)
            begin
              if (str_save7)
                vI <= res_add;
            end
          else
            vI <= 0;
        end

    end

    fix_sig_mult_nbit #(.n(n), .m(m), .k(k))
  dut_mult
  (
    .clk(clk),
    .rst(rst),
    .en(en),
    .str(str_mult),
    .mult_a(inMult_a),
    .mult_b(inMult_b),
    .mult_out(res_mult)
  );

  addsub #( .n(n))
  dut_add
  (
    .clk(clk),
    .rst(rst),
    .en(en),
    .str(str_add),
    .iVal_a(inAdd_a),
    .iVal_b(inAdd_b),
    .oVal_z(res_add)
  );


endmodule
```

15 mechOmega_elecTheta_elecOmega_noInertia_seq.v

This is a demo of the module mechOmega_elecTheta_elecOmega_noInertia_seq.v and it is part of the Power Electronics Emulator project. This code is shared as a work-in-progress version due to the internal politics of our household Institute. The released version will be available upon request.

```
`timescale 1ns/100ps
// +FHDR
    ------------------------------------------------------------------------

// Copyright (c) 2020 INAOE All rights reserved
// INSTITUTO NACIONAL DE ASTROF?SICA, OPTICA Y ELECTRONICA
// COORDINACI?N DE ELECTR?NICA
// GRUPO DE INSTRUMENTACI?N
// Confidential Proprietary
//
    ------------------------------------------------------------------------------

// FILE NAME  :   spi_rw_v0.v
// DEPARTMENT :   LABORATORIO DE SISTEMAS DIGITALES
// AUTHOR   :   Julio Hernandez
// AUTHOR?S EMAIL:  julio.hernandez@inaoep.mx
//
    ------------------------------------------------------------------------------

// RELEASE HISTORY
// VERSION DATE AUTHOR DESCRIPTION
//
// 1V0 2020-03-12 First test.
// 1V1 2020-04-02 The module is updated according to the system
    module approach
//
    ------------------------------------------------------------------------------

// PURPOSE  : This module is a dummy process.
// Note1: Due to the high amplitud on the 1/J calculation, the
    following conversion is presented
//      y= u*1/J;
//      J= 0.0000024009 -> 1/J= 416510 =~ 524288 = 1<<19
//      consider c= 504288
//      y= u*c*(1/c*J) = u*c*f
//      f= 1/(c*J)= 0.794430685498146528385188875
//    ->  y1= (u<<19)*(f)
// Note2: Due to the high amplitud on the 1/J calculation, the
    following conversion is presented
//      for the mechOmega calculation wich will be presentes P.U.
//      pmsm_mechOmega_max_krps= 0.41887902047863909846168578443727
//      mechOmega_noInertia= y
//      y= u*(1/J);
//      J= 0.0000024009 -> 1/J= 416510 ~ 524288
//      consider c= 504288
//      y= u*c*(1/c*J) = u*c*f
//      f= 1/(c*J)= 0.794430685498146528385188875
//    ->  y= (u<<19)*(f)[rps]
//      In order to change units to krps and PU
//      y_krps= y(1/1000);
```

```
//      1/1000 =~ 1/1024
//      y_krps = y(1/1024)*(1024/1000) = y(1/1024)*t = (y>>10)
    *1.024
//      y_krps = u<<9*f*t = u<<9*k = 0.8134970219501020450664334208
//      y_pu= y_krps*(1/pmsm_mechOmega_max_krps) = y_krps
    *2.3873241463784300365332564505877
//      y_pu= u<<9*k*(1/pmsm_mechOmega_max_krps) = (u<<9)
    *1.9420810835084223270873491211842

// -FHDR
    ------------------------------------------------------------------------


module mechOmega_elecTheta_elecOmega_noInertia_seq
#(  parameter   n = 32, m= 64, k=24, j=3, pmsm_dryFriction= 0,
    pmsm_visFriction= 0, spdPU_factor= 32582714, spd_shift_bit= 9,
    j_factor= 13328335, j_shift_bit= 19, pmsm_n= 67108864,
    pmsm_n_over_math_two_pi= 10680707, math_two_pi= 105414357,
    math_one= 16777215, Ts= 16)
(
  input clk, rst, str, en,
  output busy,
  input signed [n-1:0] elecTorque, mLoad,
    output signed [n-1:0] elecTheta_PU,
    output reg signed [n-1:0] elecTheta, mechOmega_PU,
    elecOmega_noInertia

);

//
    -----------------------------------------------------------------------------

// Top module local parameters definition
  localparam[5:0]
  idle  = 6'b100000,    // Idle
  stg0  = 6'b000000,    //
  stg1  = 6'b000001,    //
  stg2  = 6'b000010,    //
  stg3  = 6'b000011,    //
  stg4  = 6'b000100,    //
  stg5  = 6'b000101,    //
  stg6  = 6'b000110,    //
  stg7  = 6'b000111,    //
  stg8  = 6'b001000,    //
  stg9  = 6'b001001,    //
  stg10   = 6'b001010,    //
  stg11   = 6'b001011,    //
  stg12   = 6'b001100,    //
  stg13 = 6'b001101,    //
  stg14   = 6'b001110,    //
  stg15   = 6'b001111,    //
  stg16 = 6'b010000,    //
  stg17 = 6'b010001,    //
  stg18 = 6'b010010,    //
  stg19 = 6'b010011,    //
  stg20 = 6'b010100,    //
  stg21 = 6'b010101,    //
```

```
  stg22 = 6'b010110,    //
  stg23 = 6'b010111,    //
  stg24 = 6'b011000,    //
  stg25 = 6'b011001,    //
  stg26 = 6'b011010,    //
  stg27 = 6'b011011,    //
  stg28 = 6'b011100;    //

  localparam [7:0]    // Control local parameters
  doNothing = 8'b00000000,  //
  doLoad0   = 8'b00001000,  //
  doLoad1   = 8'b00001001,  //
  doLoad2   = 8'b00001010,  //
  doLoad3   = 8'b00001011,  //
  doLoad4   = 8'b00001100,  //
  doLoad5   = 8'b00001101,  //
  doLoad6   = 8'b00001110,  //
  doLoad7   = 8'b00001111,  //

  doSave0   = 8'b00010000,  //
  doSave1   = 8'b00010001,  //
  doSave2   = 8'b00010010,  //
  doSave3   = 8'b00010011,  //
  doSave4   = 8'b00010100,  //
  doSave5   = 8'b00010101,  //
  doSave6   = 8'b00010110,  //
  doSave7   = 8'b00010111,  //

  doTask0   = 8'b00100000,  //
  doTask1   = 8'b00100001,  //
  doTask2   = 8'b00100010,  //
  doTask3   = 8'b00100011,  //
  doTask4   = 8'b00100100,  //
  doTask5   = 8'b00100101,  //

  doGenMult = 8'b01000000,  //
  doGenAdd  = 8'b10000000;  //

  localparam [2:0]    // id values
  id_0  = 3'd0, //
  id_1  = 3'd1, //
  id_2  = 3'd2, //
  id_3  = 3'd3, //
  id_4  = 3'd4, //
  id_5  = 3'd5, //
  id_6  = 3'd6, //
  id_7  = 3'd7; //

// ----------------------------------------------------------------------------
// Top module local reg definition
  // ctrl : Run the Counter process
  reg [7:0] ctrl;

  reg [5:0] state_p, state_f;

```

```
  wire [1:0] stgDone;
  wire en_mechTheta;
  reg rstIntegral;

// -------------------------------------------------------------------------------
// Wire & Reg local definition

  wire      [j-1:0] id;

  reg signed    [n-1:0] inMult_a;
  reg signed    [n-1:0] inMult_b;
  reg signed    [n-1:0] inAdd_a;
  reg signed    [n-1:0] inAdd_b;

  wire signed   [n-1:0] res_mult;
  wire signed   [n-1:0] res_add;

  reg signed    [n-1:0] elecTorque_mLoad;

  reg signed    [n-1:0] mechTheta_noInertia_shift, mechOmega_PU_shift, acc_noInertia_shift, mechOmega_noInertia_shift;
  wire signed   [n-1:0] mechOmega_noInertia, mechTheta_noInertia;
  reg signed    [n-1:0] acc_noInertia;
  reg signed    [n-1:0] mechTheta, elecTheta_aux;
  wire signed   [n-1:0] elecTheta_PU_aux;

  wire str_load, str_mult, str_add;
  wire str_save1, str_save2, str_save3, str_save4, str_save5, str_save6, str_save7;//, str_save0;
  wire str_task0, str_task1, str_task2, str_task3, str_task4, str_task5;

// -------------------------------------------------------------------------------
// Top module assignations definition
  assign busy = state_p[5];

  assign id           = {ctrl[2],ctrl[1],ctrl[0]};

  assign str_load   = ctrl[3];
  assign str_mult   = ctrl[6];
  assign str_add    = ctrl[7];

//  assign str_save0  = (ctrl[4] & ~ctrl[2] & ~ctrl[1] & ~ctrl[0]) ? 1'b1 : 1'b0;
  assign str_save1  = (ctrl[4] & ~ctrl[2] & ~ctrl[1] &  ctrl[0]) ? 1'b1 : 1'b0;
  assign str_save2  = (ctrl[4] & ~ctrl[2] &  ctrl[1] & ~ctrl[0]) ? 1'b1 : 1'b0;
  assign str_save3  = (ctrl[4] & ~ctrl[2] &  ctrl[1] &  ctrl[0]) ? 1'b1 : 1'b0;
  assign str_save4  = (ctrl[4] &  ctrl[2] & ~ctrl[1] & ~ctrl[0]) ? 1'b1 : 1'b0;
```

```
  assign str_save5  = (ctrl[4] &  ctrl[2] & ~ctrl[1] &  ctrl[0]) ? 1'b1 : 1'b0;
  assign str_save6  = (ctrl[4] &  ctrl[2] &  ctrl[1] & ~ctrl[0]) ? 1'b1 : 1'b0;
  assign str_save7  = (ctrl[4] &  ctrl[2] &  ctrl[1] &  ctrl[0]) ? 1'b1 : 1'b0;

  assign str_task0  = (ctrl[5] & ~ctrl[2] & ~ctrl[1] & ~ctrl[0]) ? 1'b1 : 1'b0;
  assign str_task1  = (ctrl[5] & ~ctrl[2] & ~ctrl[1] &  ctrl[0]) ? 1'b1 : 1'b0;
  assign str_task2  = (ctrl[5] & ~ctrl[2] &  ctrl[1] & ~ctrl[0]) ? 1'b1 : 1'b0;
  assign str_task3  = (ctrl[5] & ~ctrl[2] &  ctrl[1] &  ctrl[0]) ? 1'b1 : 1'b0;
  assign str_task4  = (ctrl[5] &  ctrl[2] & ~ctrl[1] & ~ctrl[0]) ? 1'b1 : 1'b0;
  assign str_task5  = (ctrl[5] &  ctrl[2] & ~ctrl[1] &  ctrl[0]) ? 1'b1 : 1'b0;

  assign en_mechTheta= en & ~rstIntegral;
  assign elecTheta_PU_aux= elecTheta_aux >= 0 ? elecTheta_aux & math_one : ((elecTheta_aux + math_one) & math_one);
  assign elecTheta_PU= elecTheta_PU_aux;

  // state machine activation
  always @(state_p or str or stgDone)
    begin
      state_f <= state_p;
      ctrl <= doNothing;

      case(state_p)
        idle :
        begin
          if (str)
            begin
//              state_f <=stg0;
//              ctrl <= doLoad0;
              state_f <=stg2;
              ctrl <= doNothing;
            end
        end

        stg0 :
        begin
          state_f <= stg1;
          ctrl <= doGenMult; // dI * cosElecTheta
        end

        stg1 :
        begin
          state_f <= stg2;
          ctrl <= doSave0;
        end

        stg2 :
        begin
```

```
          state_f <= stg3;
          ctrl <= doLoad1;
        end

        stg3 :
        begin
          state_f <= stg4;
          ctrl <= doGenAdd; // qI * sinElecTheta
        end

        stg4 :
        begin
          state_f <= stg5;
          ctrl <= doSave1;
        end

        stg5 :
        begin
          state_f <= stg6;
          ctrl <= doLoad2;
        end

        stg6 :
        begin
          state_f <= stg7;
          ctrl <= doGenAdd; //  acc_noInertia
        end

        stg7 :
        begin
          state_f <= stg8;
          ctrl <= doSave2;
        end

        stg8 :
        begin
          state_f <= stg9;
          ctrl <= doTask0;
        end

        stg9 :
        begin
          state_f <= stg10;
          ctrl <= doTask1;
        end

        stg10 :
        begin
          if(stgDone[0])
            begin
              state_f <= stg11;
              ctrl <= doLoad3;
            end
        end

        stg11 :
        begin
```

```
          state_f <= stg12;
          ctrl <= doGenMult; //  qI * cosElecTheta
        end

        stg12 :
        begin
          state_f <= stg13;
          ctrl <= doSave3;
        end

        stg13 :
        begin
          state_f <= stg14;
          ctrl <= doTask2;
        end

        stg14 :
        begin
          state_f <= stg15;
          ctrl <= doTask3;
        end

        stg15 :
        begin
          if(stgDone[1])
            begin
              state_f <= stg16;
              ctrl <= doLoad4;
            end
        end

        stg16 :
        begin
          state_f <= stg17;
          ctrl <= doGenMult; // dI * sinElecTheta
        end

        stg17 :
        begin
          state_f <= stg18;
          ctrl <= doSave4;
        end

        stg18 :
        begin
          state_f <= stg19;
          ctrl <= doTask4;
        end

        stg19 :
        begin
          state_f <= stg20;
          ctrl <= doLoad5;
        end

        stg20 :
        begin
```

```
          state_f <= stg21;
          ctrl <= doGenMult; //  qI_cosElecTheta + dI_sinElecTheta
        end

        stg21 :
        begin
          state_f <= stg22;
          ctrl <= doSave5;
        end

        stg22 :
        begin
          state_f <= stg23;
          ctrl <= doTask5;
        end

        stg23 :
        begin
          state_f <= stg24;
          ctrl <= doLoad6;
        end

        stg24 :
        begin
          state_f <= stg25;
          ctrl <= doGenMult; //  betaI * math_sqrt_three_over_two,
    alphaI_over_two
        end

        stg25 :
        begin
          state_f <= stg26;
          ctrl <= doSave6;
        end

//        stg22 :
//        begin
//          state_f <= stg23;
//          ctrl <= doLoad6;
//        end
//
//        stg23 :
//        begin
//          state_f <= stg24;
//          ctrl <= doGenMult; //  betaI * math_sqrt_three_over_two
    , alphaI_over_two
//        end
//
//        stg24 :
//        begin
//          state_f <= stg25;
//          ctrl <= doSave6;
//        end
//
//        stg25 :
//        begin
//          state_f <= stg26;
```

```
//          ctrl <= doTask5;
//        end



    stg26 :
    begin
      state_f <= stg27;
      ctrl <= doLoad7;
    end

    stg27 :
    begin
      state_f <= stg28;
      ctrl <= doGenMult; //  betaI_math_sqrt_three_over_two - alphaI_over_two
    end

    stg28 :
    begin
      state_f <= idle;
      ctrl <= doSave7;
    end

    default :
      begin
        ctrl <= doNothing;
        state_f <= idle;
      end
   endcase
  end

 always @(posedge clk)
  begin
   if (rst)
    state_p <= idle;
   else
    state_p <= state_f;
  end

//  always @(ctrl or str_load)
 always @(posedge clk)
  begin

   if (str_load)
   begin
    case(id)

     id_0 :
     begin
       inMult_a <= pmsm_visFriction;
       inMult_b <=-mLoad;
     end

     id_1 :
     begin
       inAdd_a <= elecTorque;
```

```
            inAdd_b <=-mLoad;
          end

          id_2 :
          begin
            inAdd_a <= elecTorque_mLoad;
            inAdd_b <=-pmsm_visFriction;
          end

          id_3 :
          begin
            inMult_a <= mechOmega_noInertia;
            inMult_b <= pmsm_n;
          end

          id_4 :
          begin
            inMult_a <= mechOmega_PU_shift;
            inMult_b <= spdPU_factor;
          end

          id_5 :
          begin
            inMult_a <= mechTheta_noInertia_shift;
            inMult_b <= j_factor;
          end

          id_6 :
          begin
            inMult_a <= mechTheta;
            inMult_b <= pmsm_n_over_math_two_pi;
          end

          id_7 :
          begin
            inMult_a <= elecTheta_PU;
            inMult_b <= math_two_pi;
          end

          default :
            begin
              inMult_a <= 0;
              inMult_b <= 0;
              inAdd_a <= 0;
              inAdd_b <= 0;
            end
        endcase
      end

    end

//  // dI_cosElecTheta activation
//  always@(posedge clk)
//    begin
//      if(rst)
//        dI_cosElecTheta <= 0;
//      else
```

```
//         begin
//           if (en)
//             begin
//               if (str_save0)
//                 dI_cosElecTheta <= res_mult;
//             end
//           else
//             dI_cosElecTheta <= 0;
//         end
//
//     end

  // elecTorque_mLoad activation
  always@(posedge clk)
    begin
      if(rst)
        elecTorque_mLoad <= 0;
      else
        begin
          if (en)
            begin
              if (str_save1)
                elecTorque_mLoad <= res_add;
            end
          else
            elecTorque_mLoad <= 0;
        end

    end

  // acc_noInertia activation
  always@(posedge clk)
    begin
      if(rst)
        acc_noInertia <= 0;
      else
        begin
          if (en)
            begin
              if (str_save2)
                acc_noInertia <= res_add;
            end
          else
            acc_noInertia <= 0;
        end

    end

  // elecOmega_noInertia activation
  always@(posedge clk)
    begin
      if(rst)
        elecOmega_noInertia <= 0;
      else
        begin
          if (en)
            begin
```

```
              if (str_save3)
                elecOmega_noInertia <= res_mult;
            end
          else
            elecOmega_noInertia <= 0;
        end

    end

  // mechOmega_PU activation
  always@(posedge clk)
    begin
      if(rst)
        mechOmega_PU <= 0;
      else
        begin
          if (en)
            begin
              if (str_save4)
                mechOmega_PU <= res_mult;
            end
          else
            mechOmega_PU <= 0;
        end

    end

  // mechTheta activation
  always@(posedge clk)
    begin
      if(rst)
        mechTheta <= 0;
      else
        begin
          if (en)
            begin
              if (str_save5)
                mechTheta <= res_mult;
            end
          else
            mechTheta <= 0;
        end

    end

  // elecTheta_aux activation
  always@(posedge clk)
    begin
      if(rst)
        elecTheta_aux <= 0;
      else
        begin
          if (en)
            begin
              if (str_save6)
                elecTheta_aux <= res_mult;
            end
```

```
    else
     elecTheta_aux <= 0;
   end

 end

// elecTheta activation
always@(posedge clk)
 begin
  if(rst)
   elecTheta <= 0;
  else
   begin
    if (en)
     begin
      if (str_save7)
       elecTheta <= res_mult;
     end
    else
     elecTheta <= 0;
   end

 end

always @(posedge clk)
     begin
       if (rst)
         acc_noInertia_shift <= 0;
       else
         begin
           if (en)
           begin
             if (str_task0)
               acc_noInertia_shift <= acc_noInertia <<< 9;
           end
           else
             acc_noInertia_shift <= 0;
         end


     end

always @(posedge clk)
     begin
       if (rst)
         mechOmega_noInertia_shift <= 0;
       else
         begin
           if (en)
           begin
             if (str_task2)
               mechOmega_noInertia_shift <= mechOmega_noInertia <<< 7;
           end
           else
             mechOmega_noInertia_shift <= 0;
```

```
          end


      end

always @(posedge clk)
 begin
  if (rst)
   mechTheta_noInertia_shift <= 0;
  else
   begin
    if (en)
    begin
     if (str_task4)
      mechTheta_noInertia_shift <= mechTheta_noInertia <<< 3;
    end
    else
     mechTheta_noInertia_shift <= 0;
   end


 end

always @(posedge clk)
 begin
  if (rst)
   mechOmega_PU_shift <= 0;
  else
   begin
    if (en)
    begin
     if (str_task2)
      mechOmega_PU_shift <= mechOmega_noInertia;
    end
    else
     mechOmega_PU_shift <= 0;
   end


 end

always @(posedge clk)
 begin
  if (rst)
   rstIntegral <= 0;
  else
   begin
    if (en)
    begin
     if (str_task5)
     begin
      if (mechTheta >= math_two_pi || mechTheta <= -math_two_pi)
      rstIntegral <= 1;
     end
     else
```

```
              rstIntegral <= 0;
          end
          else
            rstIntegral <= 0;
        end


    end

    fix_sig_mult_nbit #(.n(n), .m(m), .k(k))
  dut_mult
  (
    .clk(clk),
    .rst(rst),
    .en(en),
    .str(str_mult),
    .mult_a(inMult_a),
    .mult_b(inMult_b),
    .mult_out(res_mult)
  );

  addsub #( .n(n))
  dut_add
  (
    .clk(clk),
    .rst(rst),
    .en(en),
    .str(str_add),
    .iVal_a(inAdd_a),
    .iVal_b(inAdd_b),
    .oVal_z(res_add)
  );

  integrator #(.n(n), .m(m), .k(k), .Ts(Ts))
  dut2(
    .clk(clk),
    .rst(rst),
    .str(str_task1),
    .en(en),
    .busy(stgDone[0]),
    .u(acc_noInertia_shift),
    .y(mechOmega_noInertia)

  );

  integrator #(.n(n), .m(m), .k(k), .Ts(Ts))
  dut3(
    .clk(clk),
    .rst(rst),
    .str(str_task3),
    .en(en_mechTheta),
    .busy(stgDone[1]),
    .u(mechOmega_noInertia_shift),
    .y(mechTheta_noInertia)

  );

```

```
endmodule
```

16 dac_hdr.v

This is a demo of the module dac_hdr.v and it is part of the Power Electronics Emulator project. This code is shared as a work-in-progress version due to the internal politics of our household Institute. The released version will be available upon request.

```
            // +FHDR
    ------------------------------------------------------------------------

// Copyright (c) 2020 INAOE All rights reserved
// +FHDR
    ------------------------------------------------------------------------

// Copyright (c) 2020 INAOE All rights reserved
// INSTITUTO NACIONAL DE ASTROF?SICA, OPTICA Y ELECTRONICA
// COORDINACI?N DE ELECTR?NICA
// GRUPO DE INSTRUMENTACI?N
// Confidential Proprietary
//
    -----------------------------------------------------------------------------

// FILE NAME  :   spi_rw_v0.v
// DEPARTMENT :   LABORATORIO DE SISTEMAS DIGITALES
// AUTHOR    :   Julio Hernandez
// AUTHORS EMAIL:  julio.hernandez@inaoep.mx
//
    -----------------------------------------------------------------------------

// RELEASE HISTORY
// VERSION DATE AUTHOR DESCRIPTION
//
// 1V0 2020-02-07 This module drives the DSG_DAS_1V1 BRD.
//            Warning!: HW dependent. Designed for DE0-Nano &
    DSG_DAS_1V1 BRD
//
    -----------------------------------------------------------------------------

// PURPOSE  : This module drives the DSG_DAS_1V1 BRD.
//          Using DIP's conf, this module reads the ADC module info
     via SPI.
//          The info obtained is send to DAC module via SPI.

//
    -----------------------------------------------------------------------------

//  PROJECT PINOUT ASSIGMENT
//
//  adc2dac.v   DE0-NANO          DSG_DAS_1V1
//
//  ADC_SCLK     (T13) GPIO_1[13]   (1) DCLK_FPGA
//  ADC_CS_N     (T14) GPIO_1[12]   (2) CS_ADC_FPGA
//  ADC_SADDR    (T12) GPIO_1[15]   (3) DIN_FPGA
//  DAC_A_CS_N    (R13) GPIO_1[14]    (4) CS_DAC0_FPGA
//  ADC_SDAT    (T11) GPIO_1[17]    (5) DOUT_FPGA
//  DAC_B_CS_N    (R12) GPIO_1[16]    (6) CS_DAC1_FPGA
//  START     (E10)   GPIO_0[27]  (7) NC
```

```
//                              (8) NC
// -FHDR
    ------------------------------------------------------------------------


module dac_hdr
#(  parameter   n = 12, ch = 2, parameter integer FREQ_FPGA_kHz =
    50000, parameter integer FREQ_SPI_x2_kHz = 40000)
  (
    input clk, rst, str,
    output busy,

    output sclk, din, dac_a_cs_n, dac_b_cs_n, dac_sel,
    input [n-1:0] dac_data_ch0,
    input [n-1:0] dac_data_ch1

  );
//
    -----------------------------------------------------------------------------

// Top module local parameters definition
  localparam[2:0]
  idle      = 3'b100, // Idle
  stage_0   = 3'b000, // Run the stage 0
  stage_1   = 3'b001, // Run the stage 1
  stage_2   = 3'b010, // Run the stage 2
  stage_3   = 3'b011; // Run the stage 2

  localparam[1:0]
  doNothing = 2'b00,  // Idle
  doDacPrc    = 2'b01,  // Run the stage 0 processes
  doStrDac    = 2'b11;  // Run activity 1

//
    -----------------------------------------------------------------------------

// Top module local wire definition

  // done : Stage 0 processes done
  wire done;

  // flag : Stage 0 proces a is done
  wire flag;

//
    -----------------------------------------------------------------------------

// Top module local reg definition
  // ctrl : Run the stage 0 processes
  reg [1:0] ctrl;
  reg [2:0]state_p, state_f;
 //
    -----------------------------------------------------------------------------

// Top module initial conditions definition
  initial state_p = idle;
```

```
//
    ---------------------------------------------------------------------------

// Wire & Reg local definition
  wire [ch-1:0] cs_n;
  wire [n-1:0] dac_data;
  wire dac_busy;

  reg [ch-1:0]    cnt;

//
    ---------------------------------------------------------------------------

// Top module assignations definition
  // I/O assigments
  assign busy   = state_p[2];
  assign dac_a_cs_n= cs_n[0];
  assign dac_b_cs_n= cs_n[1];
  assign dac_data= (cnt[0] == 0)? dac_data_ch0 : dac_data_ch1;
  assign dac_sel= cnt[1];

  // Internal module assigments
  assign flag= dac_busy ? 1'b1 : 1'b0;
  assign done= flag ?   1'b1 : 1'b0;

//
    ---------------------------------------------------------------------------

// Top module state machine
  always @(state_p or str, done)
    begin
      state_f <= state_p;
      case(state_p)
        idle :
        begin
          cnt= 2'b00;
          if(str)
          begin
            state_f <= stage_0;
            ctrl <= doStrDac;
          end
          else
            ctrl <= doNothing;
        end
        // DAC task's
        stage_0 :
        begin
          cnt= 2'b00;
          if(done)
          begin
            state_f <= stage_1;
            ctrl <= doStrDac;
          end
          else
            ctrl <= doDacPrc;
        end
        stage_1 :
```

```
        begin
          cnt= 2'b01;
          if(done)
          begin
//              state_f <= idle;       // Just for debugging proposes
//              ctrl <= doNothing;
            state_f <= stage_2;
                        ctrl <= doStrDac;
          end
          else
            ctrl <= doDacPrc;
        end
        stage_2 :
        begin
          cnt= 2'b10;
          if(done)
          begin
            state_f <= stage_3;
            ctrl <= doStrDac;
          end
          else
            ctrl <= doDacPrc;
        end

        stage_3 :
        begin
          cnt= 2'b11;
          if(done)
          begin
            state_f <= idle;
            ctrl <= doNothing;
          end
          else
            ctrl <= doDacPrc;
        end

        default :
          begin
            cnt= 2'b00;
            ctrl <= doNothing;
            state_f <= idle;
          end
      endcase
    end

  always @(posedge clk)
    begin
      if (rst)
        state_p <= idle;
      else
        state_p <= state_f;
    end

//  assign DATA = 12'h400;                    // DAC debug. 0.5 ->
    12'h800; 0.75 -> 12'hC00; 0.25 -> 12'h400;
// Interface with SPI_RW
//   - addr and data are parrallel interface
```

```
//   - din/dout are serial interface with ADC chip
spi_w_v0
  #(.FREQ_FPGA_kHz(FREQ_FPGA_kHz), .FREQ_SPI_x2_kHz(FREQ_SPI_x2_kHz
    ))   // DEVICE_FREQ_MHz= ((FREQ_SPI_x2_kHz/FREQ_FPGA_kHz)*
    FPGA_FREQ_MHz)/2
                                                  // DSG_DAS_1V1-
    DAC Frequency range: 0 - 20 MHz
  U1 (
  .iClk(clk),
  .iRst(rst),
  .iEna(ctrl[0]),
  .iStart(ctrl[1]),
  .iDevCh(cnt),
  .oDclk(sclk),
  .oDin(din),
  .cs_dac_a(cs_n[0]),
  .cs_dac_b(cs_n[1]),
  .oBusy(dac_busy),
  .iData(dac_data)
  );


endmodule
```

17 spi_w_v0.v

This is a demo of the module spi_w_v0.v and it is part of the Power Electronics Emulator project. This code is shared as a work-in-progress version due to the internal politics of our household Institute. The released version will be available upon request.

```
// +FHDR------------------------------------------------------------------------
// Copyright (c) 2020 INAOE All rights reserved
// INSTITUTO NACIONAL DE A S T R O F SICA , OPTICA Y ELECTRONICA
// C O O R D I N A C I N DE E L E C T R NICA
// GRUPO DE I N S T R U M E N T A C I N
// Confidential Proprietary
// ------------------------------------------------------------------------------
// FILE NAME  :   spi_rw_v0.v
// DEPARTMENT :   LABORATORIO DE SISTEMAS DIGITALES
// AUTHOR    :   Carlos Morales
// A U T H O R S EMAIL:  carlosj.morales@inaoep.mx
// ------------------------------------------------------------------------------
// RELEASE HISTORY
// VERSION DATE AUTHOR DESCRIPTION
//
// 1V0 2020-02-07 This module is used as read/write SPI interface.
//            Warning!: HW dependent. Designed for DSG_DAS_1V1 BRD
//            A SPI protocol selection is suggested for the next release
// ------------------------------------------------------------------------------
// PURPOSE : This module is used as read/write SPI interface.
// ------------------------------------------------------------------------------
//  PROJECT PINOUT ASSIGMENT
// -FHDR------------------------------------------------------------------------

module spi_w_v0

  #(  parameter   FREQ_FPGA_kHz = 50000,
    parameter   FREQ_SPI_x2_kHz   = 1250)
  (
    input iClk, iRst, iEna, iStart,
    input[1:0] iDevCh,
    output  oDclk, oDin,
    output reg cs_dac_a, cs_dac_b,
    output oBusy,
    input[11:0] iData
  );

```

```
  localparam
    C_max = (FREQ_FPGA_kHz/FREQ_SPI_x2_kHz) - 1,
    W_snd = 16;

  wire z, cs, rst_1;
  wire[2:0] S_CMD;
  wire[4:0] N_dclk;
  wire[5:0] ctrl_1;
  wire[W_snd-1:0] CMD;

  reg[2:0]  A;
  reg[18:0] MUX;

  assign rst_1    = ctrl_1[0] | iRst;
  assign N_dclk   = 5'd16;

  fsm_spi_v0                  U1(.clk(iClk), .rst(iRst), .ena(iEna)
    , .z(z), .send(iStart), .N_dclk(N_dclk), .dclk(oDclk), .cs(cs),
     .busy(oBusy), .ctrl(ctrl_1));
   timer_mod#(.N(28))          U2(.clk(iClk), .rst(iRst), .ena(iEna
    ), .Count_max(C_max), .z(z));
   reg_ps_nbits#(.N(W_snd))    U3(.clk(iClk), .rst(rst_1), .ena(
    ctrl_1[2]), .ld(ctrl_1[3]), .D(CMD), .q(oDin));

  // Bit assigment to match with ADC and DAC module stream
  // Warning!: HW dependent
  assign S_CMD    = {1'b1,iDevCh[1]};
  assign CMD      = {iDevCh[0],3'b011,iData};

  always @(S_CMD or cs)
    begin
      case(S_CMD)
        2'b10 : begin
            cs_dac_a <= cs;
            cs_dac_b <= 1'b1;
        end
        2'b11 : begin
                    cs_dac_a <= 1'b1;
                    cs_dac_b <= cs;
                end
        default : begin
                    cs_dac_a <= 1'b1;
                    cs_dac_b <= 1'b1;
                end
      endcase
    end

endmodule
```

18 fsm_spi_v0.v

This is a demo of the module fsm_spi_v0.v and it is part of the Power Electronics Emulator project. This code is shared as a work-in-progress version due to the internal politics of our household Institute. The released version will be available upon request.

```
//`timescale 10ns/1ps
module fsm_spi_v0(
    input clk, rst, ena, z,
          send,
    input [4:0] N_dclk,
    output dclk, cs, busy,
    output[5:0] ctrl
  );

  // ctrl[0] : rst general
  // ctrl[1] : ena timer
  // ctrl[2] : ena reg_ps
  // ctrl[3] : ld  reg_ps
  // ctrl[4] : ena reg_sp
  // ctrl[5] : rtv data

  localparam [8:0]
//    state_0 = 9'b110000000, // Espera i n s t r u c c i n de inicio
//    state_1 = 9'b010001110, // Carga datos a  e n v ar  y habilita timer
//    state_2 = 9'b000000110, // Recorre reg_ps
//    state_3 = 9'b000000010, // dclk = 0;
//    state_4 = 9'b000010010, // Captura dato de entrada
//    state_5 = 9'b001000010, // dclk = 1;
//    state_6 = 9'b010100001, // Libera datos recibidos y termina
//    state_7 = 9'b010000010;

    state_0 = 9'b110000000, // Espera i n s t r u c c i n de inicio
    state_1 = 9'b010001110, // Carga datos a  e n v ar  y habilita timer
    state_2 = 9'b000000110, // Recorre reg_ps
    state_3 = 9'b000000010, // dclk = 0;
    state_4 = 9'b000010010, // Captura dato de entrada
    state_5 = 9'b001000010, // dclk = 1;
    state_6 = 9'b010100000, // Libera datos recibidos y termina
    state_7 = 9'b010000011;

  wire   lim, rst_c;
  reg[4:0] Counter;
  reg[8:0] Qp, Qf;

  initial Qp = state_0;

  assign busy = Qp[8];
  assign cs = Qp[7];
  assign dclk = Qp[6];
  assign ctrl = Qp[5:0];

  assign rst_c = rst | Qp[0];
  assign lim   = (Counter == N_dclk) ? 1'b1 : 1'b0;
```

```verilog

  always @(Qp or send or z or lim)
    begin
      case(Qp)
        state_0 : // Espera  i n s t r u c c i n  de inicio
          if(send)
            Qf <= state_1;
          else
            Qf <= Qp;
        state_1 : // Carga datos a  e n v ar  y habilita timer
          Qf <= state_3;
        state_2 : // Recorre reg_ps
          Qf <= state_3;
        state_3 : // dclk = 0;
          if(z) begin
            if(lim)
              Qf <= state_6;
            else
              Qf <= state_4;
            end
          else
            Qf <= Qp;
        state_4 : // Captura dato de entrada
          Qf <= state_5;
        state_5 : // dclk = 1;
          if(z)
            Qf <= state_2;
          else
            Qf <= Qp;
//        state_6 : // Libera datos recibidos y se prepara para terminar
//          Qf <= state_7;
//        default : // Espera bandera para terminar
//          if(z)
//            Qf <= state_0;
//          else
//            Qf <= Qp;
        state_6 : // Libera datos recibidos y se prepara para terminar
          Qf <= state_7;
        state_7 : // Espera bandera para terminar
          if(z)
            Qf <= state_0;
          else
            Qf <= Qp;
        default :
          Qf <= state_0;

      endcase
    end

  always @(posedge clk)
    begin
      if(rst)
        Qp <= state_0;
      else if(ena)
        Qp <= Qf;
```

```
    end

  always @(posedge clk)
    begin
      if(rst_c)
        Counter <= {5{1'b0}};
      else if(Qp[4])
        Counter <= Counter + 1'b1;
    end

endmodule
```

19 timer_mod.v

This is a demo of the module timer_mod.v and it is part of the Power Electronics Emulator project. This code is shared as a work-in-progress version due to the internal politics of our household Institute. The released version will be available upon request.

```
module timer_mod
  #(parameter
    N = 4
  )(
    input  clk, rst, ena,
    input[N-1:0] Count_max,
    output z
  );

  wire[N-1:0] Count, Count_f;
  reg [N-1:0] Count_p;

  initial Count_p = 0;

  assign z = (Count_p=={N{1'b0}}) ? 1'b1 : 1'b0;

  assign Count   = (z)  ? Count_max : Count_p - 1'b1;
  assign Count_f = (rst)  ? Count_max : Count;

  always @(posedge clk)
    begin
      if(ena)
        Count_p <= Count_f;
    end

endmodule
```

20 reg_ps_nbit.v

This is a demo of the module reg_ps_nbit.v and it is part of the Power Electronics Emulator project. This code is shared as a work-in-progress version due to the internal politics of our household Institute. The released version will be available upon request.

```
//`timescale 10ns/1ps
module fsm_spi_v0(
    input clk, rst, ena, z,
          send,
    input [4:0] N_dclk,
    output dclk, cs, busy,
    output[5:0] ctrl
  );

  // ctrl[0] : rst general
  // ctrl[1] : ena timer
  // ctrl[2] : ena reg_ps
  // ctrl[3] : ld  reg_ps
  // ctrl[4] : ena reg_sp
  // ctrl[5] : rtv data

  localparam [8:0]
//    state_0 = 9'b110000000, // Espera i n s t r u c c i n de inicio
//    state_1 = 9'b010001110, // Carga datos a  e n v ar  y habilita timer
//    state_2 = 9'b000000110, // Recorre reg_ps
//    state_3 = 9'b000000010, // dclk = 0;
//    state_4 = 9'b000010010, // Captura dato de entrada
//    state_5 = 9'b001000010, // dclk = 1;
//    state_6 = 9'b010100001, // Libera datos recibidos y termina
//    state_7 = 9'b010000010;

    state_0 = 9'b110000000, // Espera i n s t r u c c i n de inicio
    state_1 = 9'b010001110, // Carga datos a  e n v ar  y habilita timer
    state_2 = 9'b000000110, // Recorre reg_ps
    state_3 = 9'b000000010, // dclk = 0;
    state_4 = 9'b000010010, // Captura dato de entrada
    state_5 = 9'b001000010, // dclk = 1;
    state_6 = 9'b010100000, // Libera datos recibidos y termina
    state_7 = 9'b010000011;

  wire   lim, rst_c;
  reg[4:0] Counter;
  reg[8:0] Qp, Qf;

  initial Qp = state_0;

  assign busy = Qp[8];
  assign cs = Qp[7];
  assign dclk = Qp[6];
  assign ctrl = Qp[5:0];

  assign rst_c = rst | Qp[0];
  assign lim   = (Counter == N_dclk) ? 1'b1 : 1'b0;
```

```

  always @(Qp or send or z or lim)
    begin
      case(Qp)
        state_0 : // Espera i n s t r u c c i n de inicio
          if(send)
            Qf <= state_1;
          else
            Qf <= Qp;
        state_1 : // Carga datos a  e n v ar  y habilita timer
          Qf <= state_3;
        state_2 : // Recorre reg_ps
          Qf <= state_3;
        state_3 : // dclk = 0;
          if(z) begin
            if(lim)
              Qf <= state_6;
            else
              Qf <= state_4;
            end
          else
            Qf <= Qp;
        state_4 : // Captura dato de entrada
          Qf <= state_5;
        state_5 : // dclk = 1;
          if(z)
            Qf <= state_2;
          else
            Qf <= Qp;
//        state_6 : // Libera datos recibidos y se prepara para terminar
//          Qf <= state_7;
//        default : // Espera bandera para terminar
//          if(z)
//            Qf <= state_0;
//          else
//            Qf <= Qp;
        state_6 : // Libera datos recibidos y se prepara para terminar
          Qf <= state_7;
        state_7 : // Espera bandera para terminar
          if(z)
            Qf <= state_0;
          else
            Qf <= Qp;
        default :
          Qf <= state_0;

      endcase
    end

  always @(posedge clk)
    begin
      if(rst)
        Qp <= state_0;
      else if(ena)
        Qp <= Qf;
```

```
    end

  always @(posedge clk)
    begin
      if(rst_c)
        Counter <= {5{1'b0}};
      else if(Qp[4])
        Counter <= Counter + 1'b1;
    end

endmodule
```

21 encoder.v

This is a demo of the module encoder.v and it is part of the Power Electronics Emulator project. This code is shared as a work-in-progress version due to the internal politics of our household Institute. The released version will be available upon request.

```
// +FHDR
    ------------------------------------------------------------------------

// Copyright (c) 2020 INAOE All rights reserved
// INSTITUTO NACIONAL DE ASTROF?SICA, OPTICA Y ELECTRONICA
// COORDINACI?N DE ELECTR?NICA
// GRUPO DE INSTRUMENTACI?N
// Confidential Proprietary
//
    -----------------------------------------------------------------------------

// FILE NAME  :   spi_rw_v0.v
// DEPARTMENT :   LABORATORIO DE SISTEMAS DIGITALES
// AUTHOR   :   Julio Hernandez
// AUTHOR?S EMAIL:  julio.hernandez@inaoep.mx
//
    -----------------------------------------------------------------------------

// RELEASE HISTORY
// VERSION DATE AUTHOR DESCRIPTION
//
// 1V0 2020-03-12 First test.
// 1V1 2020-04-02 The module is updated according to the system
    module approach
//
    -----------------------------------------------------------------------------

// PURPOSE  : This module is a dummy process.

// -FHDR
    ------------------------------------------------------------------------


module encoder
//#(  parameter   n = 32, m= 64, k=24, slit_factor= 65535,
    mechTheta_PU_factor= 2670176) // mechTheta_PU/256 //
//#(  parameter   n = 32, m= 64, k=24, slit_factor= 32767,
    mechTheta_PU_factor= 2670176) // mechTheta_PU/512 //
#(  parameter   n = 32, m= 64, k=24, slit_factor= 16383) //,
    mechTheta_PU_factor= 2670176) // mechTheta_PU/1024 //
//#(  parameter   n = 32, m= 64, k=24, slit_factor= 8191,
    mechTheta_PU_factor= 2670176) // mechTheta_PU/2048 //
//#(  parameter   n = 32, m= 64, k=24, slit_factor= 4095,
    mechTheta_PU_factor= 2670176) // mechTheta_PU/4096 //
(
  input clk, rst, en, //str,
  input signed [n-1:0] elecOmega_PU, mechTheta_PU,
    output reg enc_qA, enc_qB

);
```

```
//
    ----------------------------------------------------------------------------
// Wire & Reg local definition

  reg flg_elecOmega_PU_dir, flg_updateENC;
  reg [2:0] event_cnt;

  wire signed [n-1:0] mechTheta_slit_factor; //, mechTheta_PU;
  reg signed [n-1:0] mechTheta_slit_factor_1k;

//
    ----------------------------------------------------------------------------
// Top module assignations definition
  assign mechTheta_slit_factor = (mechTheta_PU) & slit_factor;

  always @(posedge clk)
    begin
      if (rst)
        event_cnt <= 0;
      else
        begin
          if (en)
          begin
            if (event_cnt > 3'd3)
               event_cnt <= 0;
                       else
                if (flg_updateENC)
                event_cnt <= event_cnt + 1'b1;
          end
          else
            event_cnt <= 0;
        end
    end

  always @(posedge clk)
                begin
                    if (rst)
                        flg_elecOmega_PU_dir <= 0;
                    else
                        begin
                            if (en)
                            begin
                                if (elecOmega_PU < 0)
                                    flg_elecOmega_PU_dir <= 1'b1;
                else
                  flg_elecOmega_PU_dir <= 0;
                            end
                            else
                                flg_elecOmega_PU_dir <= 0;
                        end


                end

```

```
always @(posedge clk)
       begin
         if (rst)
           flg_updateENC <= 0;
         else
           begin
             if (en)
             begin
               if (elecOmega_PU < 0)
        begin
        if (mechTheta_slit_factor > mechTheta_slit_factor_1k)
         flg_updateENC <= 1'b1;
        else
         flg_updateENC <= 0;
        end
       else
        begin
        if (mechTheta_slit_factor < mechTheta_slit_factor_1k)
         flg_updateENC <= 1'b1;
        else
         flg_updateENC <= 0;
        end

             end
             else
               flg_updateENC <= 0;
           end


       end

always @(posedge clk)
       begin
         if (rst)
           mechTheta_slit_factor_1k <= 0;
         else
           begin
             if (en)
       mechTheta_slit_factor_1k <= mechTheta_slit_factor;

             else
               mechTheta_slit_factor_1k <= 0;
           end


       end

always @(posedge clk)
       begin
         if (rst)
     begin
            enc_qA <= 0;
      enc_qB <= 0;
     end
         else
```

```
                        begin
                            if (en)
                            begin
                                if (flg_elecOmega_PU_dir)
                  begin
                                      if (event_cnt == 3'd0)
                      begin
                                    enc_qA <= 0;
                        enc_qB <= 1;
                      end
                    else
                                        if (event_cnt == 3'd1)
                        begin
                                      enc_qA <= 1;
                          enc_qB <= 1;
                        end
                      else
                                          if (event_cnt == 3'd2)
                          begin
                                        enc_qA <= 1;
                            enc_qB <= 0;
                          end
                        else
                                            if (event_cnt == 3'd3)
                            begin
                                          enc_qA <= 0;
                              enc_qB <= 0;
                            end

                  end
                else
                  begin
                                      if (event_cnt == 3'd0)
                      begin
                                    enc_qA <= 1;
                        enc_qB <= 0;
                      end
                    else
                                        if (event_cnt == 3'd1)
                        begin
                                      enc_qA <= 1;
                          enc_qB <= 1;
                        end
                      else
                                          if (event_cnt == 3'd2)
                          begin
                                        enc_qA <= 0;
                            enc_qB <= 1;
                          end
                        else
                                            if (event_cnt == 3'd3)
                            begin
                                          enc_qA <= 0;
                              enc_qB <= 0;
                            end

                  end
```

```
                               end
                               else
                  begin
                                 enc_qA <= 0;
                    enc_qB <= 0;
                  end
                           end


                  end

//   fix_sig_mult_nbit #(.n(n), .m(m), .k(k))
//   dut1
//   (
//     .clk(clk),
//     .rst(rst),
//     .en(en),
//     .str(str),
//     .mult_a(mechTheta),
//     .mult_b(mechTheta_PU_factor),
//     .mult_out(mechTheta_PU)
//   );



endmodule
```

22 signalPU.v

This is a demo of the module signalPU.v and it is part of the Power Electronics Emulator project. This code is shared as a work-in-progress version due to the internal politics of our household Institute. The released version will be available upon request.

```
// +FHDR ------------------------------------------------------------------------
// Copyright (c) 2020 INAOE All rights reserved
// INSTITUTO NACIONAL DE ASTROF?SICA, OPTICA Y ELECTRONICA
// COORDINACI?N DE ELECTR?NICA
// GRUPO DE INSTRUMENTACI?N
// Confidential Proprietary
// -----------------------------------------------------------------------------
// FILE NAME  :   spi_rw_v0.v
// DEPARTMENT :   LABORATORIO DE SISTEMAS DIGITALES
// AUTHOR   :   Julio Hernandez
// AUTHOR?S EMAIL:  julio.hernandez@inaoep.mx
// -----------------------------------------------------------------------------
// RELEASE HISTORY
// VERSION DATE AUTHOR DESCRIPTION
//
// 1V0 2020-03-12 First test.
// 1V1 2020-04-02 The module is updated according to the system module approach
// -----------------------------------------------------------------------------
// PURPOSE  : This module is a dummy process.

// -FHDR ------------------------------------------------------------------------


module signalPU
#(  parameter   n = 32, m= 64, k=24)

(
  input clk, rst, en, str,
  input signed [n-1:0] mult_a1, mult_b1, mult_a2, mult_b2, mult_a3, mult_b3,
    output signed [n-1:0] mult_out1, mult_out2, mult_out3

);

// -----------------------------------------------------------------------------
// Wire & Reg local definition


```

```
  fix_sig_mult_nbit #(.n(n), .m(m), .k(k))
  dut1
  (
    .clk(clk),
    .rst(rst),
    .en(en),
    .str(str),
    .mult_a(mult_a1),
    .mult_b(mult_b1),
    .mult_out(mult_out1)
  );

  fix_sig_mult_nbit #(.n(n), .m(m), .k(k))
    dut2
    (
        .clk(clk),
        .rst(rst),
        .en(en),
        .str(str),
        .mult_a(mult_a2),
        .mult_b(mult_b2),
        .mult_out(mult_out2)
    );

  fix_sig_mult_nbit #(.n(n), .m(m), .k(k))
    dut3
    (
        .clk(clk),
        .rst(rst),
        .en(en),
        .str(str),
        .mult_a(mult_a3),
        .mult_b(mult_b3),
        .mult_out(mult_out3)
    );



endmodule
```

23 integrator.v

This is a demo of the module integrator.v and it is part of the Power Electronics Emulator project. This code is shared as a work-in-progress version due to the internal politics of our household Institute. The released version will be available upon request.

```
// +FHDR
    ------------------------------------------------------------------------

// Copyright (c) 2020 INAOE All rights reserved
// INSTITUTO NACIONAL DE ASTROF?SICA, OPTICA Y ELECTRONICA
// COORDINACI?N DE ELECTR?NICA
// GRUPO DE INSTRUMENTACI?N
// Confidential Proprietary
//
    --------------------------------------------------------------------------------

// FILE NAME  :   spi_rw_v0.v
// DEPARTMENT :   LABORATORIO DE SISTEMAS DIGITALES
// AUTHOR   :   Julio Hernandez
// AUTHOR?S EMAIL:  julio.hernandez@inaoep.mx
//
    --------------------------------------------------------------------------------

// RELEASE HISTORY
// VERSION DATE AUTHOR DESCRIPTION
//
// 1V0 2020-03-12 First test.
// 1V1 2020-04-02 The module is updated according to the system
    module approach
//
    --------------------------------------------------------------------------------

// PURPOSE  : This module is a dummy process.
//          Using an up-counter, this module waits for the maximum
    count
//      Forward Euler method:
//      y(n) = y(n-1) + K*[t(n) - t(n-1)]*u(n-1)
//
//      Backward Euler method:
//      y(n) = y(n-1) + K*[t(n) - t(n-1)]*u(n)
//
//      Trapezoidal method:
//      y(n) = y(n-1) + K*[t(n)-t(n-1)]*[u(n)+u(n-1)]/2
//
// Note: Ts= t(n)-t(n-1). Consider a fixed Ts=
    0.00000095367431640625 (0.00000095367431640625*2^24 = 16)
// -FHDR
    ------------------------------------------------------------------------


module integrator
#(  parameter   n = 32, m= 64, k=24, Ts= 16)
(
  input clk, rst, str, en,
  output busy,
```

```
  input signed [n-1:0] u,
    output reg signed [n-1:0] y

);

// -----------------------------------------------------------------------------
// Top module local parameters definition
  localparam[1:0]
    idle  = 2'b10,  // Idle
    stg0  = 2'b00,  //
    stg1  = 2'b01;  //


  localparam [2:0]    // Control local parameters
  doNothing = 3'b000, // Idle
  doMult1   = 3'b001, // Calculate the uTs= u*Ts
  doAdder1  = 3'b010, // Calculate y_1n_uTs= y(n-1) + uTs
  doy_1n    = 3'b100; // Calculate y(n-1)= y_1n_uTs

// -----------------------------------------------------------------------------
// Top module local reg definition
  // ctrl : Run the Counter process
  reg [2:0] ctrl;

  reg[1:0] state_p, state_f;

// -----------------------------------------------------------------------------
// Wire & Reg local definition

  reg signed    [n-1:0] y_1n;
  wire signed   [n-1:0] uTs;
  wire signed   [n-1:0] y_1n_uTs;

// -----------------------------------------------------------------------------
// Top module initial conditions definition
  initial state_p = idle;
// -----------------------------------------------------------------------------
// Top module assignations definition
  assign busy = state_p[1];

  // state machine activation
  always @(state_p or str)
    begin
      state_f <= state_p;
      ctrl <= doNothing;
      case(state_p)
        idle :
```

```
        begin
          if(str)
          begin
            state_f <= stg0;
            ctrl <= doMult1;
          end
        end

        stg0 :
        begin
          state_f <= stg1;
          ctrl <= doAdder1;
        end

        stg1 :
        begin
          state_f <= idle;
          ctrl <= doy_1n;
        end

        default :
          begin
            ctrl <= doNothing;
            state_f <= idle;
          end
      endcase
    end

  always @(posedge clk)
    begin
      if (rst)
        state_p <= idle;
      else
        state_p <= state_f;
    end

  always @(posedge clk)
    begin
      if (rst)
        y_1n <= 0;
      else
        begin
          if (en)
          begin
            if (ctrl[2])
              y_1n <= y_1n_uTs;
          end
          else
            y_1n <= 0;
        end


    end

  always @(posedge clk)
    begin
      if (rst)
```

```
        y <= 0;
      else
        begin
          if (en)
          begin
            if (ctrl[2])
              y <= y_1n_uTs;
          end
          else
            y <= 0;
        end


    end

  fix_sig_mult_nbit #(.n(n), .m(m), .k(k))
  dut2
  (
    .clk(clk),
    .rst(rst),
    .en(en),
    .str(ctrl[0]),
    .mult_a(u),
    .mult_b(Ts),
    .mult_out(uTs)
  );

  addsub #( .n(n))
  dut1
  (
    .clk(clk),
    .rst(rst),
    .en(en),
    .str(ctrl[1]),
    .iVal_a( y_1n),
    .iVal_b( uTs),
    .oVal_z(y_1n_uTs)
  );




endmodule
```

24 addsubb.v

This is a demo of the module addsubb.v and it is part of the Power Electronics Emulator project. This code is shared as a work-in-progress version due to the internal politics of our household Institute. The released version will be available upon request.

```
// +FHDR ------------------------------------------------------------------------
// Copyright (c) 2020 INAOE All rights reserved
// INSTITUTO NACIONAL DE ASTROF?SICA, OPTICA Y ELECTRONICA
// COORDINACI?N DE ELECTR?NICA
// GRUPO DE INSTRUMENTACI?N
// Confidential Proprietary
// -----------------------------------------------------------------------------
// FILE NAME  :   addsub.v
// DEPARTMENT :   GRUPO DE SISTEMAS DIGITALES
// AUTHOR     :   Julio Hernandez
// AUTHOR?S EMAIL:  julio.hernandez@inaoep.mx
// -----------------------------------------------------------------------------
// RELEASE HISTORY
// VERSION DATE AUTHOR DESCRIPTION
//
// 1V0 200509 First test. This module is based on the adder example of intel web page.
//        For more information see intel.com
// -----------------------------------------------------------------------------
// PURPOSE  : This module generates a generic adder and determines the output in the
//        same clock cycle. This block has the form y= a+b.
// -FHDR ------------------------------------------------------------------------


module addsub
#(  parameter   n   = 8)
(
  input clk, rst, en, str,
  input signed [n-1:0] iVal_a,
  input signed [n-1:0] iVal_b,

  output reg signed [n-1:0] oVal_z
);

  initial oVal_z = 0;

  always @ (posedge clk)
  begin

```

```
    if(rst)
      oVal_z <= 0;
    else
      begin
        if (en)
          begin
            if (str)
              oVal_z <= iVal_a + iVal_b;
          end
        else
          begin
            oVal_z <= 0;
          end
      end

  end

endmodule
```

25 fix_sig_mult_nbit.v

This is a demo of the module fix_sig_mult_nbit.v and it is part of the Power Electronics Emulator project. This code is shared as a work-in-progress version due to the internal politics of our household Institute. The released version will be available upon request.

```
// +FHDR
    ------------------------------------------------------------------------

// Copyright (c) 2020 INAOE All rights reserved
// INSTITUTO NACIONAL DE ASTROF?SICA, OPTICA Y ELECTRONICA
// COORDINACI?N DE ELECTR?NICA
// GRUPO DE INSTRUMENTACI?N
// Confidential Proprietary
//
    -----------------------------------------------------------------------------

// FILE NAME  :   fix_sig_mult_nbit.v
// DEPARTMENT :   GRUPO DE SISTEMAS DIGITALES
// AUTHOR   :   Julio Hernandez
// AUTHOR?S EMAIL:  julio.hernandez@inaoep.mx
//
    -----------------------------------------------------------------------------

// RELEASE HISTORY
// VERSION DATE AUTHOR DESCRIPTION
//
// 1V0 200509 First test. This module is based on the adder example
     of intel web page.
//        For more information see intel.com
//
    -----------------------------------------------------------------------------

// PURPOSE  : This module generates a generic muliplier and
    determines the output in the
//        same clock cycle. This block has the form y= a*b.
// NOTE:    This module assumes that in_a, in_b and out_z have the
    same data width.
// -FHDR
    ------------------------------------------------------------------------



module fix_sig_mult_nbit
  #(  parameter n   = 8,
  parameter   m   = 16,
  parameter   k   = 4)

  (
  input clk, rst, en, str,
  input signed  [n-1:0] mult_a,
  input signed  [n-1:0] mult_b,

  output reg signed [n-1:0] mult_out
  );

```

```
  initial mult_out = 0;

  wire signed   [m-1:0] mult_aux;

  assign mult_aux = mult_a * mult_b;

  always@(posedge clk)
    begin
      if(rst)
        begin
          mult_out <= 0;
        end
      else
        begin
          if (en)
            begin
              if (str)
                begin
                  mult_out <= mult_aux[((n+k)-1):k];
                end
            end
          else
            begin
              mult_out <= 0;
            end
        end

    end

endmodule
```
